# Mainstreams of Horizontal Gene Exchange in Enterobacteria: Consideration of the Outbreak of Enterohemorrhagic *E. coli* O104:H4 in Germany in 2011

**DOI:** 10.1371/journal.pone.0025702

**Published:** 2011-10-14

**Authors:** Oliver Bezuidt, Rian Pierneef, Kingdom Mncube, Gipsi Lima-Mendez, Oleg N. Reva

**Affiliations:** 1 Bioinformatics and Computational Biology Unit, Department of Biochemistry, University of Pretoria, Pretoria, South Africa; 2 Laboratoire de Bioinformatique des Génomes et des Réseaux (BiGRe), Université Libre de Bruxelles, Bruxelles, Belgium; Royal Tropical Institute, The Netherlands

## Abstract

**Background:**

*Escherichia coli* O104:H4 caused a severe outbreak in Europe in 2011. The strain TY-2482 sequenced from this outbreak allowed the discovery of its closest relatives but failed to resolve ways in which it originated and evolved. On account of the previous statement, may we expect similar upcoming outbreaks to occur recurrently or spontaneously in the future? The inability to answer these questions shows limitations of the current comparative and evolutionary genomics methods.

**Principal Findings:**

The study revealed oscillations of gene exchange in enterobacteria, which originated from marine γ-Proteobacteria. These mobile genetic elements have become recombination hotspots and effective ‘vehicles’ ensuring a wide distribution of successful combinations of fitness and virulence genes among enterobacteria. Two remarkable peculiarities of the strain TY-2482 and its relatives were observed: i) retaining the genetic primitiveness by these strains as they somehow avoided the main fluxes of horizontal gene transfer which effectively penetrated other enetrobacteria; ii) acquisition of antibiotic resistance genes in a plasmid genomic island of β-Proteobacteria origin which ontologically is unrelated to the predominant genomic islands of enterobacteria.

**Conclusions:**

Oscillations of horizontal gene exchange activity were reported which result from a counterbalance between the acquired resistance of bacteria towards existing mobile vectors and the generation of new vectors in the environmental microflora. We hypothesized that TY-2482 may originate from a genetically primitive lineage of *E. coli* that has evolved in confined geographical areas and brought by human migration or cattle trade onto an intersection of several independent streams of horizontal gene exchange. Development of a system for monitoring the new and most active gene exchange events was proposed.

## Introduction

**Figure 1:**
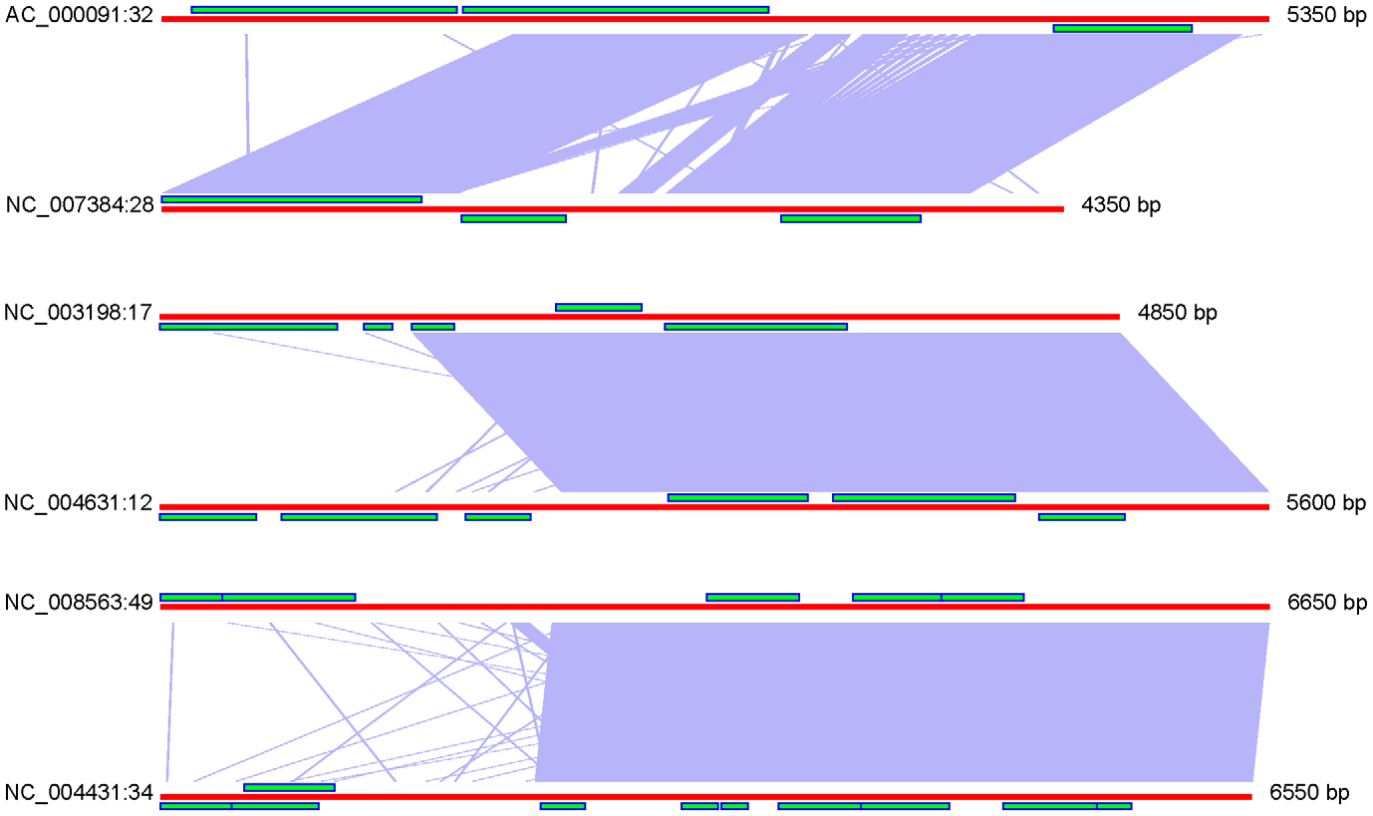
Comparison of DNA sequences of three pairs of homologous GIs found in different enterobacterial genomes. Despite DNA similarity detected by bl2seq, the prediction of coding genes shown as green bars is not consistent. More information about these GIs is available at http://anjie.bi.up.ac.za/geidb/geidb-home.php.

DNA fragments encoding enzymes, transcriptional regulators and virulence factors are fluxing through bacterial taxonomic walls by horizontal gene transfer (HGT). These elements often endow environmental and clinical strains of bacteria with new properties, including an enhanced virulence [1]. Lateral genetic exchange, particularly of drug tolerance genes has been recognized for a long time; however, our understanding of this phenomenon is limited. Ontology and phylogeny of laterally transferred genetic elements are difficult to investigate, let alone the predictions of their insertion sites in hosts chromosomes. DNA and protein sequence similarity comparison by blast is generally used to track the origins of genomic islands (GIs) but this approach has many limitations. Horizontally transferred genes are highly mutable and similarities in their DNA sequences vanish very quickly [2], [3]. Protein sequence similarity reflects mostly a functional conservation rather than phylogenetic relations between GIs. Moreover, the majority of genes found in GIs are hypothetical and in many instances falsely predicted. Consider a general situation with the annotation of GIs as illustrated in Fig. 1. Even a clearly visible DNA similarity between GIs found in different genomes does not ensure any consistency in gene prediction performed by different authors and annotation methods. Moreover, multiple genes which are of great importance for mobility of plasmids and phages quickly become a wreck after integration into chromosomes due to multiple mutations and fragmentations [2], [3].

An outbreak of the lethal *Escherichia coli* in Europe in 2011 highlighted the shortcoming of our knowledge on the basic principles of evolutionary trends of new pathogens. The outbreak first occurred in Germany in May 2, 2011 where a rare enterohemorrhagic strain *Escherichia coli* O104∶H4 caused haemolytic-uremic syndrome. The infection spread fast through many other European countries and sickened thousands of people. The level of lethality associated with the production of Shiga toxin by the strain and its resistance against many antibiotics was significant. A number of isolates (TY-2482, LB226692, 01-09591, GOS1 and GOS2) from this outbreak have been sequenced and annotated [4]–[6]. Based on the unique combination of genomic features these strains were suggested to represent a new pathotype Entero-Aggregative-Haemorrhagic *E. coli* (EAHEC) [4]. In this work TY-2482 was used for the analysis. The presence of several new virulence determinants in this strain which are absent in the closest relative strain *E. coli* 55989, – a causative agent of the previous outbreak in 2002 in central Africa, – suggested the involvement of HGT in its evolution; however, the origin of this new deadly pathogen remained obscure until now.

The study of genome oligonucleotide usage (OU) signatures has a long history dating back to early publications by Karlin et al. [7]–[9], who focused mainly on dinucleotide compositional biases and their evolutionary implications. Statistical approaches of OU comparison were further advanced by Deschavanne et al. [10], who applied chaos game algorithms; and by Pride et al. [11], who extended the analysis to tetranucleotides using Markov Chain Model simulations. Later, a number of practical tools based on OU statistics have become publicly available, particularly: for phylogenetic comparison of bacterial genomes [12], [13]; identification of horizontally transferred genomic islands [14]–[22] and assignment of unknown genomic sequences [23], [24]. These approaches exploited the notion that genomic OU composition was less variable within genomes rather than between them, regardless of which genomic locus had been taken into consideration [25].

The exchange of genetic material was found to have occurred in different domains of life: Archaea, Bacteria, and Eukarya [26]. HGT, defined as a mechanism that promotes the transfer of foreign genomic segments between lineages, was found to be relatively common in prokaryotes and less common in higher-order organisms. The transfer of operational genes is a continual process and is far more important in prokaryotic diversity of different sources [27], [28]. For HGT to be successful, the acquisition of foreign DNA segments must be counterbalanced by DNA loss. Acquired DNA providing functions that are beneficial to the host may be maintained, while DNA providing less beneficial functions is to be lost [29]–[31]. Mobile genetic elements possess genes that contribute to bacterial speciation and adaptation to different niches, but also carry with them factors that contribute to the bacteria's fitness traits, secondary metabolism, antibiotic resistance and symbiotic interactions [3], [32]–[34].

DNA compositional comparisons between lineages have uncovered that genes acquired by the different HGT mechanisms [35]–[37] display features that are distinct from those of their recipient genomes [1], [14]. Genes acquired by HGT can often display atypical sequence characteristics and a restricted phylogenetic distribution among related strains, thereby producing a scattered phylogenetic distribution [28], [36]. Bacterial species are variable in their overall GC content but the genes in genomes of particular species are fairly uniform with respect to their base composition patterns and frequencies of oligonucleotides [28]. The phylogenetic aspect of similarity in base composition among closely related species arises from their common origin. Similarity is also influenced by genome specific mutational pressures that act upon their genes to promote the maintenance of composition stability. Core genes in a given organism exhibit homogeneous OU content and codon usage, while foreign genes display atypical characteristic features shared with their mobilomes (phages and conjugative plasmids) or previous host organisms for the genetic segments which were mobilized and integrated by mobilomes [38]. Compositional specificity of GIs allows their identification in chromosomal sequences as previously described [18].

Virulence determinants such as toxins, adhesins, polysaccharide capsule synthesis proteins and iron uptake system are not encoded by the core genome but by accessory pathogenicity islands (PAIs) [39]. The effects of acquisition of virulence factors on a bacterial organism depend on its genetic background, which includes the core genome organisation and presence/absence of other virulence factors [40]. It has been supposed that the extra-intestinal virulence of *E. coli* may be a coincidental by-product of commensalism [41]. Other authors suggested that the development of vaccines specific for extra-intestinal infections will be extremely difficult as many other targets may likely be present in commensal strains [42] due to the random gene exchange. The same PAI from *E. coli* may be found in *Shigella* and *Salmonella*, and many other homologous GIs have been reported to be present in even more distant organisms such as *Yersinia* and *Bordetella* [39], [43]. To summarize, the present-day model of the evolution of pathogenic bacteria from commensals or environmental organisms considers this process very much as a gene dice followed by the selection of unique combinations of factors and genetic determinants rendering the pathogenicity. To study the hidden layers of gene exchange between enterobacteria and to elucidate the role in which HGT may play in the process of pathogenicity development we applied three innovative approaches: clustering of GIs by OU pattern similarity; stratigraphic analysis of inserts of horizontally acquired genomic elements and identification of donor-recipient relations between microorganisms in terms of gene exchange. The applicability of these methods was tested on the analysis of TY-2482 genome.

## Results

### Genomic island identification

Each genome may be characterized by a unique pattern of frequencies of oligonucleotides [11], [14], [23]. Foreign DNA inserts retain OU patterns of the genomes of origin. Comparison of OU patterns of genomic fragments against the entire genome OU pattern reveals areas with alternative DNA compositions. An algorithm for the identification of horizontally transferred genomic elements by superimposition of OU statistical parameters has been introduced in our previous publications [18], [22]. The four parameters which are implemented in the algorithm are as follows: D – distance between local and global OU patterns measured as a percentage of the maximum possible distance; RV – relative variance of oligonucleotide frequency distribution normalized by frequencies of nucleotides in a DNA fragment; GRV – in contrast to the previous parameter, variance was normalized by nucleotide frequencies calculated for a complete genome; and PS (pattern skew) – the distance (D) between two strands of the same DNA molecule. Horizontally transferred GIs are characterized by a significant pattern deviation (large D), significant increase in GRV associated with decreased RV and a moderately increased PS [18]. Exploitation of all these parameter allows the discrimination of the putative mobile genomic elements from other genomic loci with alternative DNA compositions, namely: multiple tandem repeats, clusters of genes for ribosomal proteins and ribosomal RNA [22].

To facilitate a large scale analysis of bacterial genomes, a Python utility SeqWord Sniffer has been developed. It is available for download from www.bi.up.ac.za/SeqWord/sniffer/. SW Sniffer predictions obtained from the parameters set for D > = 1.5 sigma deviation and GRV/RV > = 1.5 were compared to three IslandViewer tools for GI identification, – IslandPick, SIGI-HMM and IslandPath, – exploiting different sequence and composition based approaches for prediction of GIs [44]. The rate of false negative predictions was determined by testing the capacities of different programs to predict known PAIs from PAI DB [45], [46]. An approximate rate of false positive predictions was estimated by counting the total number of GIs predicted by one program and not confirmed by others. Confirmation means that coordinates of a GI predicted by one program at least partly overlap with coordinates predicted by another program. Rates of the false negative and unconfirmed predictions are shown in Tables S1 and S2, respectively. Table 1 summarizes this statistics. SW Sniffer was chosen for this study as it outperformed other tested programs in terms of prediction of PAIs, but at the expense of the increased rate of unconfirmed predictions (Table 1). However, not all unconfirmed GIs are false positive as far as 37 out of 51 known PAIs fall into this category, i.e. they were predicted only by one program (Table S1). In this work we first searched for GIs in a set of 637 bacterial genomes representing different taxonomic classes. More stringent parameters were set: sigma D > = 2; GRV/RV > = 2 and PS < = 55%. The PS parameter was set at 55% to set GIs apart from clusters of ribosomal RNA genes which are characterized by extremely high PS values. Consequently, sequences predicted to comprise of putative GIs were searched by nucleotide blast against a local database of 16S rRNA to further filter out false predictions. Detailed information about the 2,622 GIs predicted from the above mentioned 637 bacterial genomes is available on-line in GEI-DB (http://anjie.bi.up.ac.za/geidb/geidb-home.php). GIs stored in the GEI-DB database were used as a training set for this study. Table S3 lists 1,254 GIs of the training set which most likely are true positive as they share DNA and composition similarity. Only 55% of them were confirmed jointly by IslandViewer algorithms (Table S1). If to allow 45% of unconfirmed GIs as an acceptable level, the rate of false positive predictions by SW Sniffer with the parameters set by default is below 5%. The meaning of a false positive prediction is different for different programs depending on the used algorithm. For SW Sniffer the false positive prediction means a selected genomic fragment with an alternative DNA composition that is not attributed to a lateral acquisition but to a random variation of local OU patterns in the core genome sequence.

**Table 1:** Comparison of different programs of GI identification.

Program	False negative rate	Rate of unconfirmed predictions
SeqWord Sniffer*	12%	50%
IslandPick	94%	38%
SIGI-HMM	41%	28%
IslandPath	69%	42%

*Settings for SeqWord Sniffer were: D > = 1.5 sigma deviation per genome; GRV/RV > = 1.5 and PS < = 55%. These are the settings by default.

The SeqWord Sniffer with the parameters set by default (D > = 1.5 sigmas; GRV/RV > = 1.5) was further used to identify GIs in a set of 1,237 sequenced bacterial chromosomes and plasmids including the draft sequence of *E. coli* TY-2482. As a result, a total of 11,870 putative GIs were predicted (http://www.bi.up.ac.za/SeqWord/sniffer/gidb/index.php). These were used in this work as a reference database to search for GIs of other organisms sharing homology in OU pattern, gene and DNA sequences with GIs of enterobacteria.

**Figure 2:**
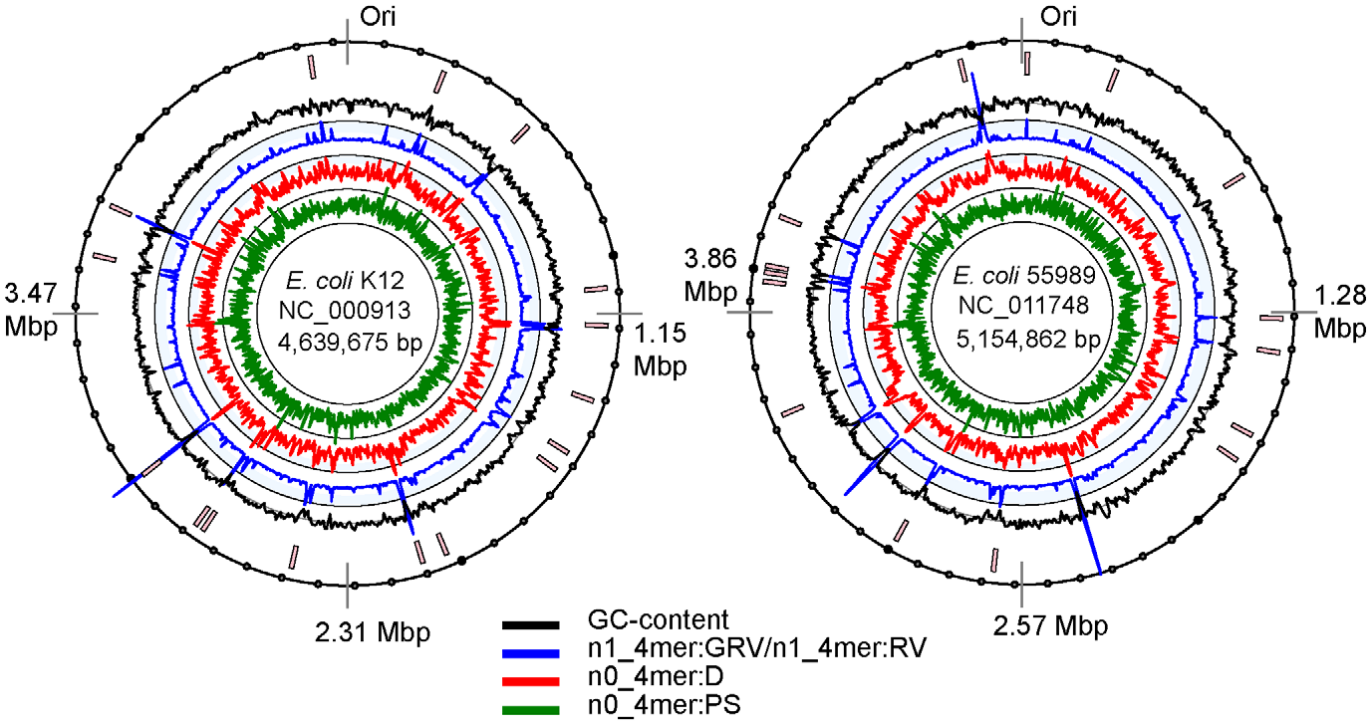
Distribution of GIs in the genomes of commensal *E. coli* K12 and enterohemorrhagic *E. coli* 55989 as predicted by SeqWord Sniffer with parameters set as: D > = 1.5 sigmas; GRV/RV > = 1.5.

Fig. 2 depicts positions of GIs predicted in the commensal *E. coli* K12 and enterohemorrhagic *E. coli* 55989 strains, a causative agent of the outbreak in central Africa in 2002, which is now considered as an ancestor of the newly isolated *E. coli* TY-2482. It was observed that the chromosomes of *E. coli* contain approximately the same number of GIs found in more or less similar positions (Fig. 2). It corroborates with a previously reported observation that in *E. coli* gene acquisition and loss takes place at precisely the same locations known as integration hotspots [42]. Thus, a general overview of availability of GIs may not aid much in understanding why some strains are pathogenic and others are not.

A complete genome sequence of *E. coli* TY-2482, which is now available as a draft comprising multiple contigs was also searched for GIs by SW Sniffer. Seventeen GIs were predicted and found to be similar to those in *E. coli* 55989 except for one, which most likely is associated with a plasmid.

### Clustering of genomic islands

**Figure 3:**
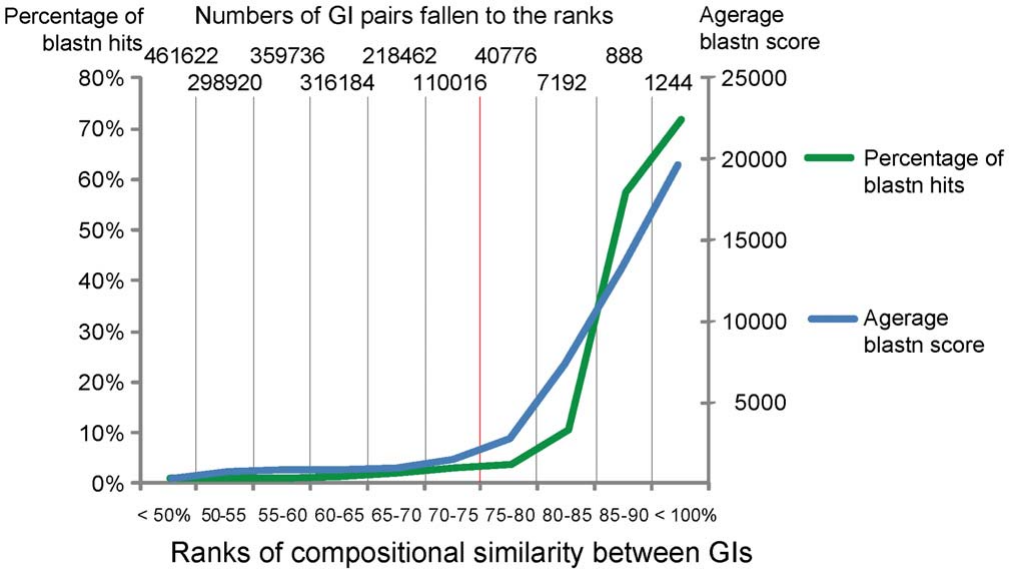
Percentage of blastn hits and average scores calculated for pairs of GIs ranked by compositional similarity values. The threshold value used for clustering of GIs is depicted by a vertical red line.

**Figure 4:**
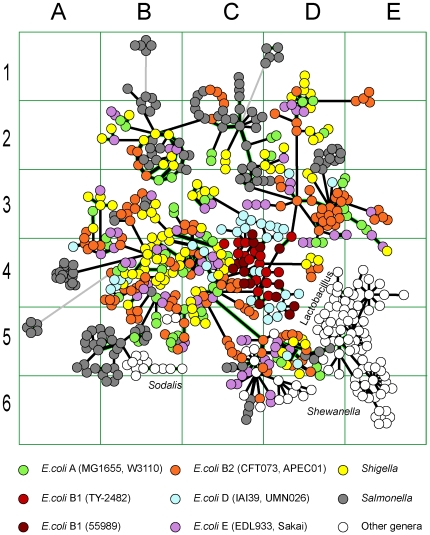
Clusters of GIs sharing OU pattern similarity. Each node corresponds to one GI. Two nodes of GIs that share 75% or more compositional similarity are linked by a black edge. Grey edges represent similarity below 75%. Links between nodes of GIs which were confirmed by blastn to share similarity in sequence are highlighted in green.

Groups of clusters of GIs were created basing on OU pattern similarity. Compositional similarity was measured as 100% – D. It was assumed that the sharing of compositional similarity by GIs may indicate their common provenance. For homologous GIs the level of sequence similarity is expected to be higher than that of a randomly selected pair of GIs. To validate this hypothesis pairs of GIs from the training set were searched for similarity by OU patterns and bl2seq. Then the pairs of GIs were ranked by the compositional similarity values as shown in Fig. 3. For each rank the numbers of GIs producing significant blastn hits (scored above 100) were counted and then average blastn scores for each rank were calculated. Similarity of randomly generated DNA sequences is expected to be around 50%. It was found that the pairs of GIs showing similarity between 50% and 70% were almost equally random, but a further increase in compositional similarity between GIs is associated with a steep increase in percentage of pairs of GIs showing sequence similarity. The compositional similarity above 90% is largely attributed to GI duplicates. Hence a pattern similarity index of 75% was chosen as an optimal threshold for clustering of related GIs, which may or may not share identical DNA sequences. In total 1,305 clusters were created; however, 1,158 of the total clusters were singletons. The biggest cluster consisted of 1,119 GIs. The outermost elements of this cluster may not share similarity in either composition or sequence, but they are linked by intermediate elements. GIs (designated as nodes) which shared 75% compositional similarity or more were connected by edges. An in-house Python script with Graphviz incorporation and a graph pruning criterion implementation was used to create these clusters. The pruning method was implemented as follows: if three nodes in a graph are interlinked, the edge representing the smallest similarity percentage gets pruned. The script consequently determines sequence similarity between linked GIs by bl2seq algorithm and generates a graphical output shown in Fig. 4. The graph in Fig. 4 is only a part of the biggest cluster that contains the GIs of enterobacteria. More information about each GI is available through an interactive web-page at http://www.bi.up.ac.za/SeqWord/maps/enterobact.html. It was shown in the study that the *Escherichia*'s GIs are very similar to GIs of *Shigella*, *Salmonella* and several other more distant genera. These GIs are grouped into several sub-clusters, many of which are polyphyletic, meaning that they comprise GIs found in organisms belonging to different genera. GIs of *E. coli* of the phenotypic groups A, B2 and E are versatile and share the same sub-clusters with the GIs from *Shigella* and very often with those found in *Salmonella*. On the contrary, GIs of *E. coli* strains TY-2482 and 55989 (group B1) are clustered all together in cells C4-D4 in Fig. 4. The GIs of *E. coli* in group D are moderately variable.

### Stratigraphic analysis of genomic islands

**Figure 5:**
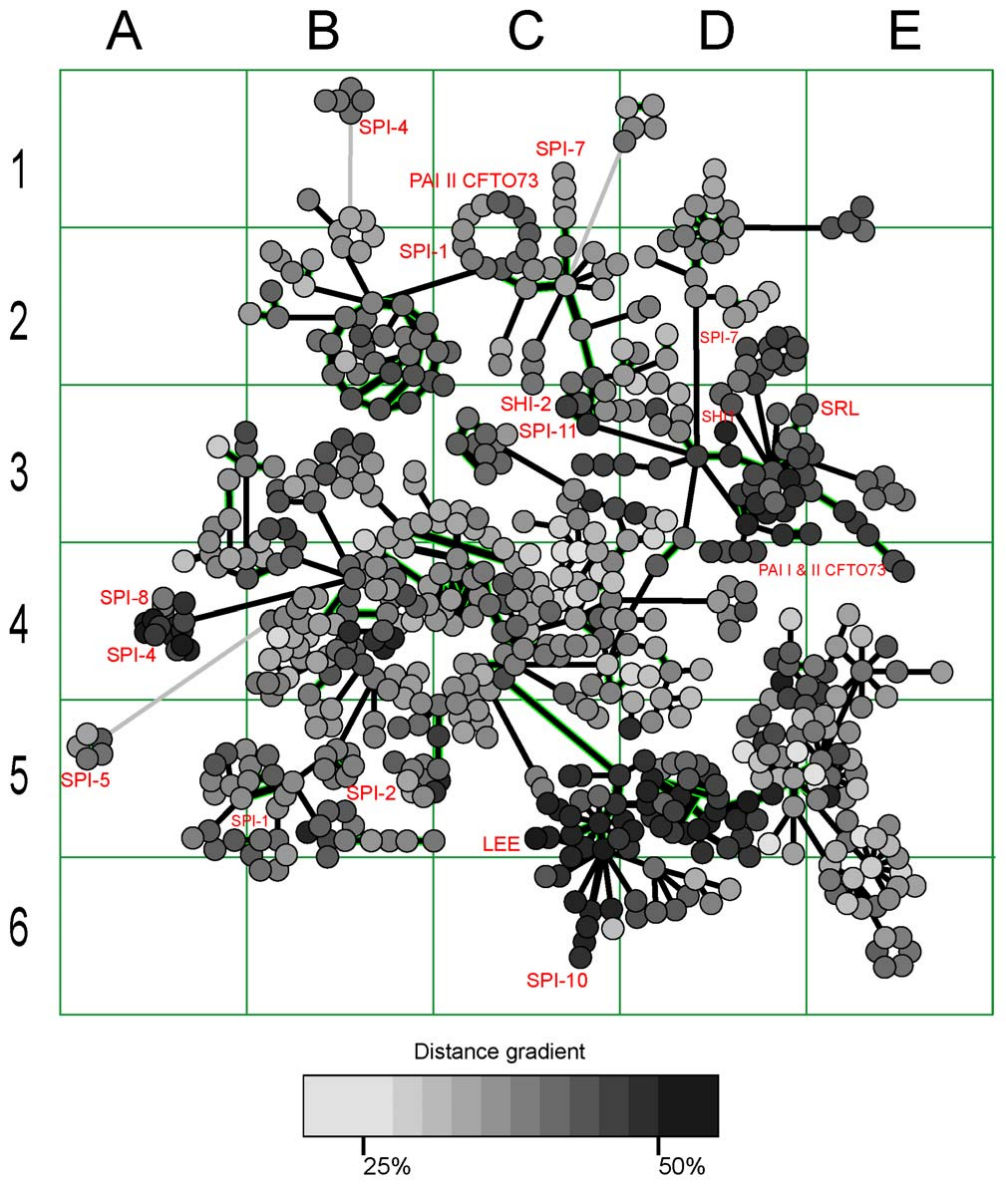
Stratigraphic analysis of enterobacterial GI inserts. Layout of GIs is the same as in Fig. 4. Gradient colours depict divergences of GI patterns from the complete genome OU patterns of their host organisms. The older inserts are depicted by lighter colours, as their OU patterns are much closer to their hosts. Known pathogenicity islands from PAI DB are mapped on the plot.

Inserts of foreign DNA undergo a process of amelioration, which influences their OU patterns to start to reflect the OU pattern of their host chromosomes overtime [47], [48]. Horizontally acquired GIs which are used by the host organism evolve much faster to optimize the transcription and translation of their constituent genes [49]. Selfish genes and non-coding sequences do not get affected by amelioration as much as the functional genes; however their compositions also change overtime. We hypothesized that the comparison of GIs of the same origin which are distributed in organisms that share similar genomic OU patterns would result in different D-values relative to the time of their acquisition. In Fig. 5 the differences in D-values are depicted by grey colour gradients. The darker colour gradients depict GIs which have been acquired recently, while the lighter colours depict ancient acquisitions. Known PAIs from PAI DB [45], [46] were mapped also by their names.

GIs of enterobacteria were acquired not simultaneously but sequentially in different spans of time. The central part of the graph (cell C4) contains the most ancient inserts. By comparing the graphs in Fig. 4 and 5 one may conclude that the genomes of *E. coli* B1 contain predominantly old GIs inherited from an ancestor which is also common for *Shigella*. The only relatively recent acquisition is GI #12 of *E. coli* 55989 (in Fig. 2 counted clockwise from the chromosomal origin of replication) and its corresponding island in *E. coli* TY-2482 which encode a secreted autotranspoter toxin. This toxin is an important virulence factor for uropathogenic *E. coli* [50] and may explain to some extend the pathogenicity of the strains TY-2482 and 55989. It however cannot be brought to conclusion that this toxin had a major influence on the latest outbreak caused by TY-2482. Fig. 4 illustrates that this GI is not a recent acquisition as it has been present in more ancient *E. coli* strains. The most recently acquired GIs of enterobacteria are grouped in cells C5-D6, and these comprise LEE and SPI-10 PAIs. Other recent acquisitions are in cells D3-E3 (SHI-1, SHI-7, SRL, PAI I and PAI II) and A4 (SPI-4, SPI-8).

### Donor-recipient relations

GIs of recent acquisitions are known to constitute OU patterns of former hosts which allow the possibility to re-trace their donor organisms. The donor-recipient relations should not be oversimplified as an act whereby pieces of DNA are randomly donated from one organism to another. Although naked DNA fragments may be transformed directly into the chromosome of an environmental organism [51] that was reported particularly for *Yersinia* [52], the latter seem not to be the most effective way of DNA exchange. Phages and plasmids may have a major contribution towards HGT [2], [31], [43]. Vectors such as conjugative plasmids contain hundreds of functional genes which may be transferred to a new host in a single evolutionary event [37]. In many instances plasmids are seen to comprise phage integrases [2], [43]. These allow them to actively integrate genetic cassettes into chromosomes of unrelated organisms [53] and mobilize random genomic fragments that they excise from organisms which they sequentially infect. Plasmids undergo the same amelioration process as horizontally transferred GIs. They acquire traits of their most predominant host organism and consequently start to reflect their OU composition. Donor-recipient relationships between microorganisms may be considered as sharing the same pool of mobilomes which are predominant in one organism. Such a mobilome is in turn dispersed sporadically among other phylogenetically diverse organisms that dwell in the same habitat. It was hypothesised that the genetic material is more likely to be transferred from a predominant owner of the mobilome towards rarely infected organisms, and that comparing OU patterns of GIs to their hosts would result in the possibility to establish donor-recipient relations between different organisms and their constituent GIs.

**Figure 6:**
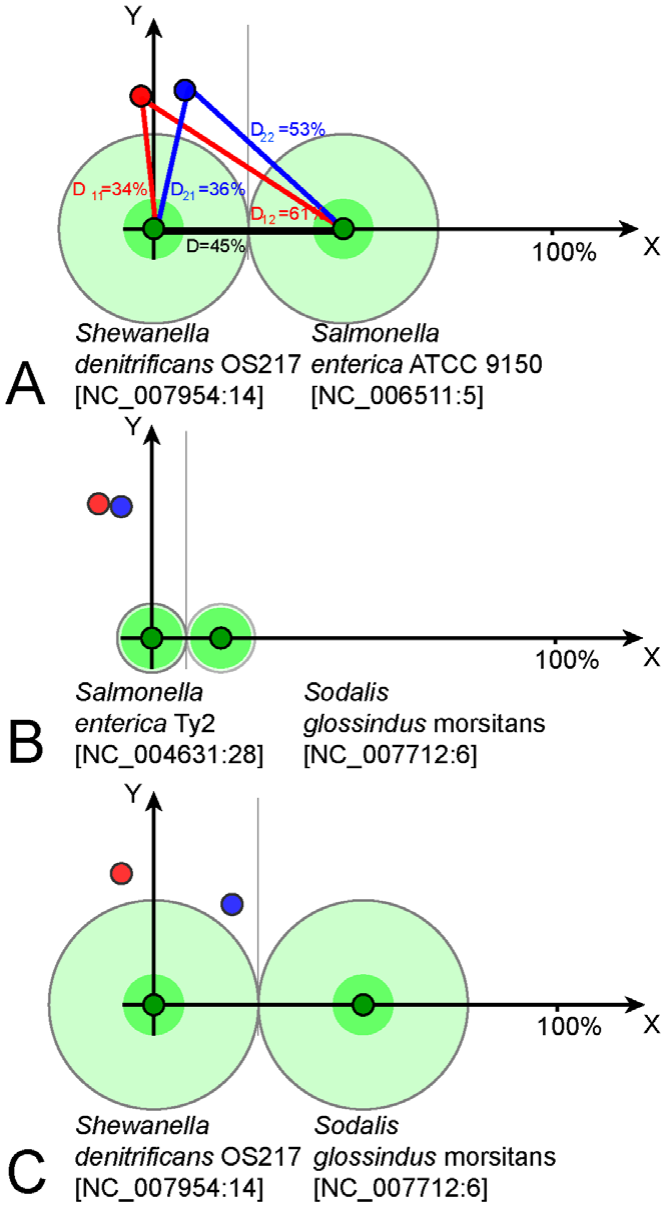
Donor recipient relationships determined for GIs from *Salmonella*, *Shewanella* and *Sodalis*. X axis shows the distance between host chromosomes depicted by dark green circles. Shaded green areas around circles outline intergenomic variability of local OU patterns. Shaded light green areas around genome circles and an intermediate grey vertical line depict the half-distance between chromosomal OU patterns. GIs of the organisms on the left and right are shown as red and blue circles, respectively. Y values for the GIs are based on the distance values calculated between OU patterns of these GIs and their host chromosomes as explained in ‘Materials and methods’. GI IDs are as in GEI-DB. A) Comparison of GIs NC_007954:14 and NC_006511:5 from *S. denitrificans* OS217 (3177400..3199999) and *S. enterica* ATCC 9150 (858550..886299). Distance values used in the calculations are shown. B) Comparison of GIs NC_004631:28 and NC_007712:6 from *S. enterica* Ty2 (2847050.. 2866799) and *S. glossindus* morsitans (1186850.. 1242349). C) Comparison of GIs NC_007954:14 and NC_007712:6 from *S. denitrificans* OS217 and *S. glossindus* morsitans.

For instance, the GIs of *Shewanella denitrificans* OS217 [NC_007954:14] and *Salmonella enterica* ATCC 9150 [NC_006511:5] were used to consider donor-recipient relations. These GIs share similarity in both sequence and composition (in Fig. 4 they are represented by two linked nodes in the cell D5) but the OU patterns of the host chromosomes differ. Distances calculated for the chromosomes and GIs of these organisms are shown in Fig. 6A. Pattern distances for both GIs showed to be closer to the *Shewanella* chromosome. This organism was therefore designated to be the donor of GI [NC_006511:5] harboured by *Salmonella*. Donor-recipient relations may be disclosed when genomic OU patterns are substantially dissimilar just as illustrated with *Shewanella* and *Salmonella*. In a pair of homologous GIs from *Salmonella* and *Sodalis* (Fig. 6B; these GIs in Fig. 4 are in the cell B5) donor-recipient relation was hard to determine, as the pattern distances for both islands deviate from either chromosomes. However, it may be assumed that the GI movement was most likely from *Salmonella* to *Sodalis* rather than the other way round (Fig. 6C); however, a number of intermediate owners of this mobile element may be expected between *Shewanella* and *Sodalis*.

### Categories of genes distributed by horizontal transfer

Protein coding sequences located in 2,622 GIs of the training set were clustered using Markov Clustering Algorithm (MCL). The MCL preformed sufficiently and it consequently clustered the coding sequences into favourable classes. The top 24 clusters each comprising more than 50 genes from different GIs were annotated and categorized into functional groups (Table 2).

**Table 2:** Annotation of genes of the top 24 MCL clusters.

Cluster #	Gene annotation	Functional group	Number of genes
1	ABC-transporters, ATP-binding proteins	Transport	252
2	Transposases	Selfish phage and plasmid related	213
3	Histidine kinase sensor response regulators	Translation and transcription regulation	162
4	Glycosyl transferases	Polysaccharide and O-antigen biosynthesis	153
5	IS1, iso-IS1	Selfish phage and plasmid related	153
6	Glucose epimerases and dehydratases	Polysaccharide and O-antigen biosynthesis	129
7	Glycosyl transferases	Polysaccharide and O-antigen biosynthesis	127
8	IS600 transposases	Selfish phage and plasmid related	93
9	Fimbrial proteins	Membrane and adhesive proteins	88
10	IS2 transposases and integrases	Selfish phage and plasmid related	85
11	Fimbrial periplasmic chaperons	Membrane and adhesive proteins	78
12	GGDEF diguanylate cyclases	Translation and transcription regulation	77
13	Outer membrane usher proteins	Membrane and adhesive proteins	75
14	Integrases	Selfish phage and plasmid related	73
15	Aminotransferases	Polysaccharide and O-antigen biosynthesis	71
16	Dehydrogenase reductases	Enzymes of unknown specificity	67
17	Acetyle transferases	Polysaccharide and O-antigen biosynthesis	65
18	RHS family proteins	RHS family proteins	64
19	Resolvases and recombinases	Selfish phage and plasmid related	63
20	Thymidylyl and uridylyl transferases	Polysaccharide and O-antigen biosynthesis	63
21	IstB transposases	Selfish phage and plasmid related	60
22	Transcription regulators	Translation and transcription regulation	53
23	IS1 ORF1	Selfish phage and plasmid related	52
24	Transcription regulators	Translation and transcription regulation	51

GIs often comprise one to several genes of the same cluster. Chi-square test showed that the frequency of co-occurrences of genes from different clusters is on the level of a random combination except for the clusters 4, 6, 7 and 20 (all are of the polysaccharide and O-antigen biosynthesis functional group) showing a tendency for co-occurrence in GIs; and the clusters 11 and 13 which almost always form a pair of a fimbrial periplasmic chaperon and outer membrane usher protein in GIs.

**Figure 7:**
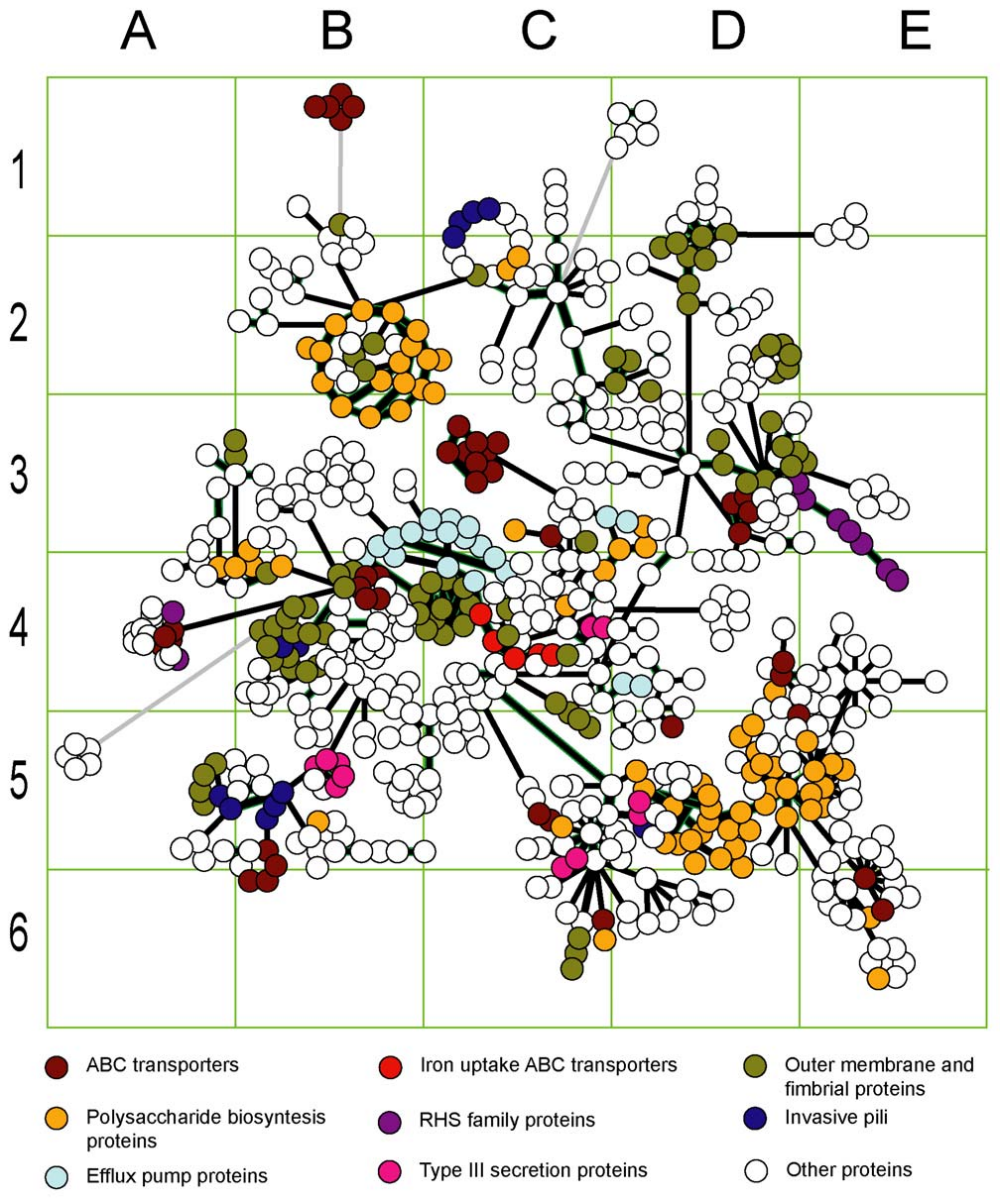
The illustration of distribution patterns of genes of different functional groups of the GIs which were identified in enterobacteria. Layout of GIs is the same as in Figs. 4 and 5.

Distribution of genes of different clusters and functional groups in enterobacterial GIs is shown in Fig. 7. Groups of ABC-transporters and RHS family proteins are represented by the clusters 1 and 18 (Table 2), respectively. The group ‘Outer membrane and fimbrial proteins’ is a combination of the clusters 9, 11 and 13; and the group ‘Polysaccharide biosynthesis proteins’ is a combination of the clusters 4, 6, 7, 15, 17 and 20. Phage and plasmid associated transposases, integrases, resolvases, helicases and IS-elements are most widely distributed in all GIs, illustrating the role of phages and plasmids in HGT. Classes of selfish mobility genes are not depicted in Fig. 7 as they are present almost in every GI. Transcriptional and translational regulators are very important in virulence development. Acquisition or loss of GIs often has complex effects on the host bacterium including regulatory effects on many core genes [54]. GGDEF diguanylate cyclases may have a profound effect on the bacterial behavioural response towards environmental stimuli [55]. However, since the discovery of the role of each individual regulator protein in bacterial pathogenicity requires a substantial experimental study, such could not be afforded in this work; we therefore did not consider this group of genes, which are overrepresented in GIs. In addition to the groups defined in Table 2, three more groups of genes which are specific for the enterobacterial PAIs were added: efflux pump proteins, type III secretion proteins and invasive pili.

Within the ABC-transporter class the majority of the transporters were found to be for amino acids and oligopeptides. ABC-transporters are substrate specific with the oligopeptide transporters multi-specific, controlling nearly all di- and tripeptides. ABC-transporters are the second most abundant protein-coding genes in all sequenced genomes following transposases [56]. It is not clear whether these genes are frequent in GIs because of general abundance, or they are abundant in bacterial genomes owing to horizontal exchange and adaptiveness. The latter explanation looks plausible because these genes are not distributed randomly among GIs but create functional modules that are required by organism in relation to the substrates with which they occur. Specificity for the type of transporters pertains to the added strain on energy resources and limitations of cell surface of an organism. The exchange for amino acid transporters has further effects on development, growth and signalling.

A separate group of transporters in cell C4 (Fig. 7) comprises iron uptake and transport proteins. A well developed iron uptake system is a prerequisite for the virulent and commensal bacteria [57], [58]. All these GIs are old inserts acquired by the *E. coli* ancestor.

Outer membrane and fimbrial proteins are important virulence factors [39], [59]. The prevalence of genes encoding outer membrane proteins in chromosomal hot spots and plasmids was reported by Nogueira et al. [60]. These authors associated mobile outer membrane proteins with cooperative trait determinants important in shaping the microbial social behaviour of both pathogenic and commensal bacteria. Fimbrial biosynthesis is performed through a chaperone/usher dependant pathway whereby an operon encodes at a minimum three different proteins: a fimbrial subunit, a chaperone and an usher. These fimbrial operons represent hypervariable DNA regions and are frequently involved in HGT as observed in enterobacteria. Outer membrane and fimbrial proteins are a prerequisite for initial adhesion to host epithelium. The route to infection within the host includes a variety of different habitats that require fimbriae for adhesion and colonization.

Polysaccharide biosynthesis contributes to biofilm formation and O-antigen variability of the cell surface. Genes involved in O-antigen and polysaccharide biosynthesis occur as clusters on chromosomes with evidence of interspecies transfer of the entire cluster among *Shigella*, *Salmonella* and *Escherichia* (cells D5–E5, B2, C2 and B4 in Fig. 7). The role of O-antigen variability in conferring virulence and pathogenicity of enterobacteria is broadly discussed in literature [30], [61]–[63].

Recombination or retrotransposon hotspot (RHS) protein family is a source of large repetitions and mediator for chromosomal rearrangements. The production of a cell surface protein with a macromolecular binding function through the RHS-core ORF has been suggested for *E. coli* and proposed that this is critical to the organism with the RHS distribution, which is correlated to the *E. coli* population structure [64]. The role of the RHS protein family appears to be in association with ligand-binding and cell-surface rather than chromosomal rearrangements [65], [66]. RHS proteins constitute several relatively recent HGT in *Escherichia* (cells E3–E4) and *Salmonella* (cell A4 in Fig. 7).

Type III secretion proteins, invasive pili and efflux pumps are the most notorious virulence factors of enterobacteria [67]–[70]. Despite an obvious sequence similarity of type III secretion proteins present in different GIs, DNA compositional analysis showed that these virulence factors evolved independently several times by gene recombination. Type III secretion system in the SPI-2 PAI of *Salmonella* (cell B5) is well characterized [68], [71]. This is a relatively old gene insert in contrast to the type III secretion proteins of the PAI LEE in *Escherichia* which are of recent horizontal gene exchange activity (C5–D5 in Fig. 7). Much older inserts of type III secretion proteins were found in *E. coli* GIs shown in Fig. 7 in the cell C4.

Genes encoding the invasive pili are present in 3 separate clusters in cells B5, B4 and C1. Most likely they evolved independently by gene rearrangements.

Efflux pump proteins are involved in drug resistance [72]. There are many dissimilar genes encoding efflux pump proteins of different families that are distributed among the GIs of enterobacteria, and not all of them are depicted in Fig. 7. Cells B3–C4 represent a group of conserved GIs which comprise a pair of efflux proteins denoted as multidrug resistant proteins K and Y that are associated with a conserved two component sensor transcriptional regulator. All these GIs are old inserts which are harboured by *E. coli* of different phenotypic groups. A significant sequence conservation of these genes shows that these genes still are important for survival of *E. coli*.

Many GIs exist as functional modules, which provide host organisms with specific functionality. The functional relatedness between GIs was statistically proven by calculating SIG values, as previously described [73]. Functionally related GIs are represented in the GEI-DB (http://anjie.bi.up.ac.za/geidb/geidb-home.php) as MCL neighbours. The analysis of functional modules of genes has been proposed recently as a tool for reconstructing the evolution and phylogenetic links between prophages [74]. The current study showed that this approach should be used cautiously when the evolution of PAIs is under consideration. OU pattern comparison (Fig. 7) demonstrated that the GIs of the same functional module may re-appear many times independently, probably at each oscillation of a new vector. The evolution of phages should be separated from the evolution of functional gene modules even if they are the ‘passenger compartments’ of these phages. The phage evolution is driven by optimization of self-reproducibility; while the appearance and evolution of horizontally transferred genetic modules should be considered in terms of the evolution/ecology concept of the host microorganism [43], [60].

### Genomic islands of the pathogenicity plasmids of the EAHEC strains

**Figure 8:**
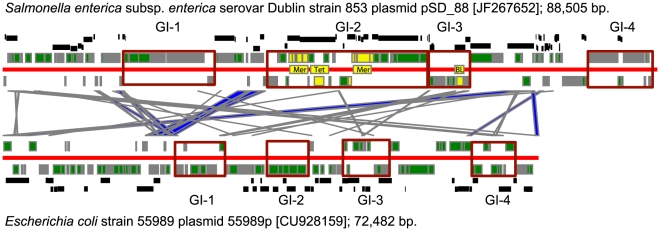
A graphical representation of the bl2seq comparative analysis conducted for the sequences of pathogenicity plasmids pSD_88 and 55989p. Protein coding genes are shown by green bars, hypothetical genes by grey bars and drug resistance operons are depicted by yellow bars and labels: Mer for mercury resistance operon; Tet for tetracycline resistance gene and BL for beta-lactamase. The red horizontal lines separate plasmid genes by their direction of transcription. Blast hits between two plasmids are depicted by blue connecting stripes. Predicted GIs in plasmid sequences are framed and named respectively. Corresponding positions of the contigs of TY-2482 which were mapped against plasmid sequences by blastn are depicted by black bars.

The genome of enterohemorrhagic *E. coli* 55989 strain comprises a large plasmid 55989p of 74,482 bp which most likely contributes towards its virulence [4]. Many contigs of the new isolate TY-2482 show sequence similarity to both this plasmid and the multidrug resistance plasmid pSD_88 from *S. enterica* ssp. *enetrica* Dublin (Fig. 8). The latter observation suggests that TY-2482 most likely contains a hybrid pathogenicity plasmid established from both 55989p and pSD_88. These two plasmids are thought to have ascended from a common ancestor as they both possess the same fragments of housekeeping genes (Fig. 8). Both plasmids contain at least 4 horizontally transferred GIs whose OU patterns are dissimilar to both each other and the plasmid core sequences. The only similarity found in plasmids 55989p and pSD_88 was between their core sequences. On the contrary, the contigs of TY-2482 partially overlap with both plasmids core sequences and GIs: GI 2, 3 and 4 of pSD_88 and GI 2, 3 and 4 of 55989p. The exchange of mobile elements from these plasmids was facilitated by the presence of more than 20 transposases and IS-elements in their sequences.

**Figure 9:**
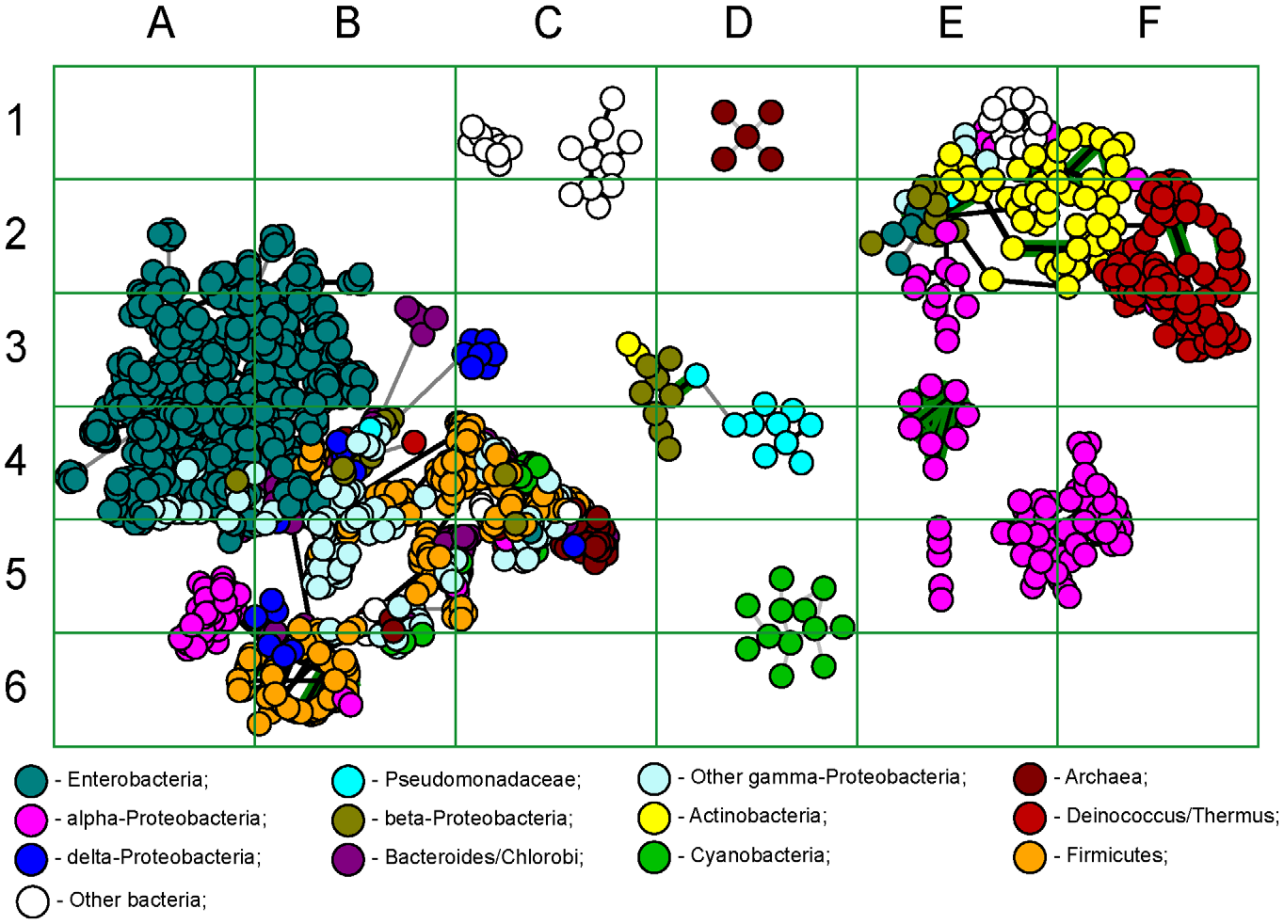
Overview of clusters of GIs from different bacterial classes.

The virulence of the strain TY-2482 is very likely facilitated by the presence of GI-2 of pSD_88 which comprises genes encoding drug resistance proteins (a broad range beta-lactomase and tetracycline efflux protein TetA) and mercury detoxication proteins. In contrast to other GIs of the plasmids pSD_88 and 55989p, GI-2 of pSD_88 shows no compositional similarity to the GIs of enterobacteria which were discussed above. Fig. 9 is an overview of clusters of GIs from different bacterial classes. The major cluster of enterobacterial GIs (Fig. 4) is shown in cells A2–B4. Newly found drug resistance GIs fell into another quite distant cluster shown in cells E1–F3. The central and oldest elements of this cluster are mycobacterial GIs which spread to *Deinococcus* / *Thermus* and *Acidobacteria*. More recent homologous inserts were found in β-Proteobacteria and these are the GIs which show the greatest compositional and gene content similarity to the plasmid associated GIs from *Salmonella* and *Escherichia* (the cell E2 in Fig. 9). Many of these GIs contain mercury resistance operons, which may be inherited from ancestral mycobacterial plasmids such as the mercury resistance plasmid isolated from *Mycobacterium abscessus* [75].

An interactive map of clusters of GIs shown in Fig. 9 is available for viewing, zooming and stratigraphic coloring at www.bi.up.ac.za/SeqWord/maps/map.html.

**Figure 10:**
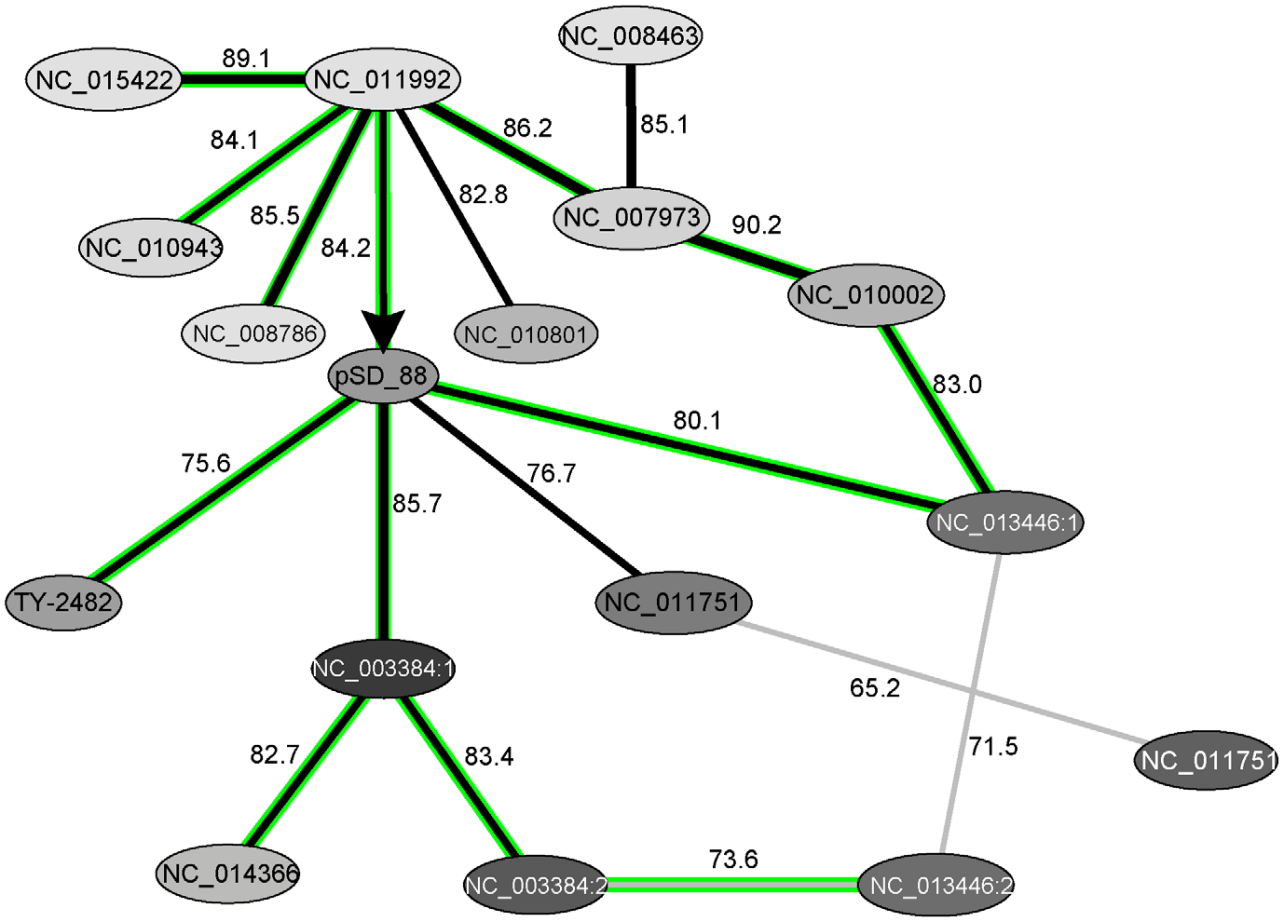
Stratigraphic analysis and OU pattern similarity relations between GIs showing similarity to the mercury resistance GI of the plasmid pSD_88. Each node corresponds to one GI named as in Table 2. Values for OU pattern similarity shared between nodes are shown above the edges. Gradient colours depict divergences of GIs patterns from the complete genome patterns of their host organisms as in Fig. 5. Putative direction of GI transfer NC_011992 to pSD_88 is depicted by an arrow (see also Fig. 11).

A nucleotide blast search of GI-2 of pSD_88 through the database of predicted GIs discovered similar DNA fragments in several other organisms of different classes (Table 3). GIs of enterobacteria which were identified by blast to be similar to GI-2 of *S. enterica* ssp. *enetrica* Dublin plasmid pSD_88 were found to be in possession of large mercury resistance operons accompanied by the genes that encode the broad range beta-lactamase and tetracycline efflux proteins [76]. Nucleotide blast search also retrieved similar heavy metal resistance operons in GIs of several γ- and β-Proteobacteria. OU pattern comparison revealed that many of them are dissimilar in DNA composition and they probably have originated from different sources. GIs of γ-Proteobacteria are recent acquisitions. Their OU patterns are remarkably distant from their host's chromosomal OU patterns. Homologous GIs in β-Proteobacteria are older inserts. Possible donor-recipient interactions between these GIs and their hosts are visualized in Fig. 10.

**Figure 11:**
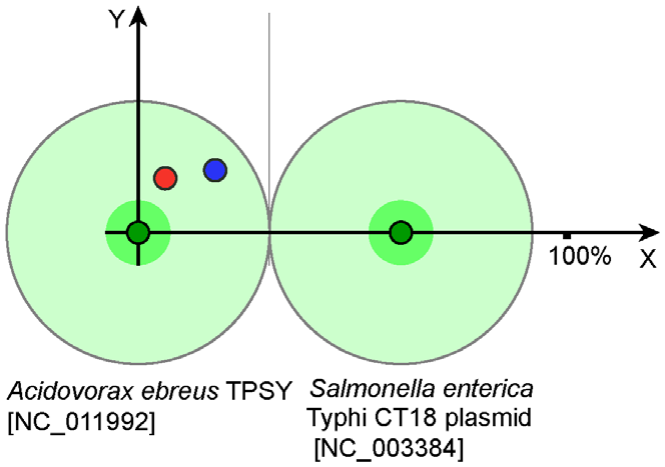
Donor-recipient relationship determined between GIs from *A. ebreus* TPSY and *S. enterica* Typhi CT18. Meanings of the graph elements were explained in Fig. 6.

**Table 3:** Blast results of GIs of other bacterial genomes which showed a significant sequence similarity to GI-2 of *S. enterica* ssp. *enetrica* Dublin plasmid pSD_88.

Genomic island	Annotation*	Score	S%†	D%‡
*Escherichia coli* TY-2482 [contigs 00033_1, 00499_1 and 00546_1]	mer, βl, tet	9501	76	32
*Salmonella enterica* ssp. *enterica* Typhi CT18 plasmid, NC_003384:1 [157158..177822]	mer, βl, tet	4301	86	42
*Comamonas testosteroni* CNB-2, NC_013446:1 [2861000..2881851]		3811	80	36
*Nitrosomonas europaea* ATCC 19718, NC_004757 [905417..925311]	mer, tet	3313	39	36
*Acinetobacter baumannii* ACICU, NC_010611 [243356..264198]		2961	57	61
*Acidovorax ebreus* TPSY, NC_011992 [2277714..2295906]	mer, tet	2584	84	23
*Shewanella frigidimarina* NCIMB 400, NC_008345 [4109193..4146599]	mer	2469	17	53
*Nitrosomonas eutropha* C91, NC_008344:2 [1089057..1108455]	mer	2311	27	32
*Salmonella enterica* subsp. *enterica* Typhi CT18 plasmid, NC_003384:2 [107500..125897]	mer	2250	83	39
*Delftia acidovorans* SPH-1, NC_010002 [2932123..2951187]		2106	82	30
*Marinobacter aquaeole*i VT8, NC_008740 [1414926..1435099]		2063	53	44
*Shigella dysenteriae* Sd197, NC_007606 [3752925..3771840]		1804	41	33
*Cupriavidus metallidurans* CH34, NC_007973 [3238831..3258471]	tet	1790	83	27
*Comamonas testosteroni* CNB-2, NC_013446:2 [2776491..2801174]		1658	70	37
Gamma proteobacterium HdN1, NC_014366 [586254..605651]		1538	82	29
*Corynebacterium urealyticum* DSM 7109, NC_010545 [1781393..1801974]		1485	79	29
*Salmonella enterica* ssp. *enterica* Heidelberg SL476, NC_011083 [1525960..1544455]	βl	1383	48	37
*Alicycliphilus denitrificans* K601, NC_015422 [3514585..3535258]	mer	1306	82	22
*Nitrosomonas eutropha* C91, NC_008344:1 [230541..250543]	mer	1294	23	34

Remarks:

*mer – mercury resistance operon; βl – beta-lactamase; tet – tetracycline efflux protein.

† S% – compositional similarity of GIs to OU pattern of GI-2 of *S. enterica* Dublin plasmid pSD_88.

‡ D% – distance between GIs' OU patterns and OU patterns of the host chromosomes.

The stratigraphic analysis shown in Fig. 10 demonstrates the acquisition of these metal resistance genes by enterobacteria. According to the analysis, enterobacteria acquired these genes from β-Proteobacteria, *Acidovorax ebreus* being the most likely donor (Fig. 11). *A. ebreus* TPSY and many other β-Proteobacteria possessing the mercury resistance operon inhabit environmental areas which are contaminated with heavy metals [77]. Pathogenic bacteria utilize mercury resistance genes for drug and disinfectant resistance [78],[79]. A large part of the plasmid pSD_88 with neighbouring beta-lactamase encoding genes was identified in the genome of uncultured γ-Proteobacteria HdN1 [NC_014366] that demonstrates that this GI may be easily integrated into the chromosome.

## Discussion

Horizontal gene exchange plays an important role in the dispersion of virulence factors and emergence of new pathogens. Genes encoding toxins and drug resistance proteins are often located in plasmids and highly variable genomic fragments which presumably are of lateral origin [1], [2], [16], [31], [39], [43], [45], [72], [76], [80]. HGT sometimes is considered to be a random and time-constant process of casual gene exchange events which may happen at any time between any organisms. The approaches of genome linguistics introduced in this paper have uncovered two important features of HGT: (i) the direction of GI distribution and (ii) its relative timing. The stratigraphic analysis conducted on enterobacteria (Fig. 5) indicated that the GIs which they possess are results of acquisitions in different time frames. In Fig. 5 the majority of the GIs appeared to be old acquisitions and several clusters showed to be populated predominantly by new acquisitions. In this work we did not separate prophages from transposons and integrated plasmids. It is known that in enterobacteria prophages are substantially accountable for the exchange of virulence determinants [2]. But the same authors reported significant technical difficulties in distinguishing prophages from other mobile elements due to the lack of reliable genetic markers. Pallen and Wren [43] considered enterobacterial PAIs as a separate class of mobile element clearly distinguishable from prophages and plasmid inserts, however, it remained unclear whether PAIs may be transfered between genomes independently without the assistance of plasmids or phages or not.

In bacterial genomes prophages adjoin inserts of other mobile elements in genome recombination hotspots (see Fig. 2). It is very difficult to separate two adjacent GIs even by a manual inspection of the genetic content. Many GIs are in fact mosaic chimeras of several independently evolved mobile genetic elements which are either composed of a single replicon as reported in lambiod phages possessing passenger compartments of foreign genes [43]; or simply co-occur on the host chromosome. To avoid speculations, in this work we focused on the analysis of the inserts which showed compositional similarity to other predicted GIs without attempting to distinguish between different types of mobile genetic elements.

Phylogenetic relationships between *E. coli* and *Shigella* species are rather confusing. A complete genome comparison study conducted by Touchon et al. [42] confirmed the existence of four well separated phylogenetic groups of *E. coli* referred to as: A, B1, B2 and E. These have previously been defined based on multi-locus enzyme electrophoresis and various DNA markers [81]–[83]. The additional group D according to Touchon et al. is paraphyletic [42]. The authors also illustrated that the *Shigella* and *E. coli* groups A, B1 and E which occupy the same cluster share a more recent common ancestor in contrast to *E. coli* of groups B2 and D. Our analysis revealed that the OU compositions of GIs of *Shigella* and *E. coli* of groups A, E and B2 are much more variable as compared to those in the *E. coli* strains of group B1which are the causative agents of the recent enterohemorrhagic outbreaks. Strains 55989, IAI1 and the new isolate TY-2482 appear to be genetically ‘primitive’ organisms, as they all are in possession of the oldest GIs inserts which probably have been inherited from a common ancestor of *Escherichia* and *Shigella*. A centric phylogenetic position of O104:H4 strains in relation to other groups of *E. coli* was also demonstrated by other authors in a minimum-spanning tree on the basis of allelic profiles of core genes obtained from another sequenced outbreak strain EHEC 01-09591 and the reference *E. coli* genomes [6]. The general assumption is that enterohemorrhagic strains are natural inhabitants of cattle intestines and may potentially cause infections to humans upon exposure [84]–[86]. Inhabitation of cattle intestines does not explain how genomes of the outbreak microorganisms were closely confined from the mainstreams of horizontal gene exchange, nighter does it explain why the widespread bovine *E. coli* do not cause outbreaks among humans on a daily basis if that is the cause. Even if the outbreak strains and O104:H4 cattle *E. coli* are closely related organisms, the relation between them is however not straightforward. A plausible explanation to the outbreak phenomenon may be that these strains were confined in areas with very limited or difficult access until recently. Indeed, *E. coli* strains isolated from faeces of healthy humans in industrialized countries showed a distinction from those isolated in remote tropical islands [87]. Genetic naivety of these organisms makes them more vulnerable to the effects of newly acquired virulence factors, which in strains 55989 and TY-2482 are associated predominantly with plasmids, except for one chromosomal insert containing the gene for secreted autotranspoter toxin and, probably, genes for Shiga toxin subunits. Contig 00415_1 of *E. coli* TY-2482 is 98% similar to the fragment of *stx2* Shiga toxin gene of *E. coli* O157:H7 Thai-12. It correlates with the reported synthesis of the Shiga toxin by the outbreak strains [6]. Only the short and probably non-functional 564 bp fragment of the 4,426 bp long sequence of stx2 AB was found to be present in the strain 55989. This DNA fragment is located 10 kbp downstream of the GI #8 (Fig. 2). Contig 00415_1 is not associated with either of the predicted GIs of TY-2482. However, it has to be noted that the prediction of GIs from randomly concatenated contigs is rough and cannot ensure the detection of all inserts.

There were several sequential fluxes of global gene exchange reflected in Fig. 4 and 5 in clusters A3–B3, B4, B2, D3 and C5–D5. These events affected genomes of *Shigella* and *E. coli* of groups A and B2, but somehow passed round *E. coli* B1. The former organisms are thought to have utilized their acquired genes well for pathogenicity and/or commensalism; and probably gained immunity to further inserts of these mobilomes. The GIs of these organisms, be it pathogens or commensals, share the same clusters with each other and the GIs of *Salmonella*. However it does not necessarily mean that these organisms have arbitrarily exchanged genes with one another. Alternative scenarios may be that they all were infected from the same source, or that *Salmonella* acted as a donor of GIs to *Escherichia*, or vice versa. The OU patterns of these organisms are so similar to each other, making it almost impossible to give preference to either scenario. Interestingly, all the GIs of *E. coli*, *Shigella* and *Salmonella* show compositional similarity to the OU patterns of *Shewanella*. Homology between GIs of these organisms was confirmed by nucleotide blast. For instance, the blast analysis revealed a homologous cluster of polysaccharide biosynthesis genes shared by GIs of *Salmonella enterica* ATCC 9150 (NC_006511:5) and *Shewanella denitrificans* OS217 (NC_007954:14). These islands were further analysed for similarity in OU composition and *Salmonella enterica* ATCC 9150 was found to have acquired its GI from the *Shewanella* lineage (Fig. 6A). Many of the well studied GIs of *E. coli* and *Salmonella* shown in Fig. 5: cells D5–E6 including LEE and SPI-10 PAIs are recent acquisitions and share a high degree of similarity in composition and sequence with their counterparts of environmental *Shewanella*. However, the virulence genes harboured by these PAIs are not of *Shewanella* origin but own enetrobacterial virulence determinants. A global search by OU pattern similarity through the database of putative GIs showed that the root of transmission for the entire set of GIs most likely is *Vibrio*, particularly the plasmids of *V. fischeri* (data not shown). GIs harboured by these organisms proved to be the oldest inserts, which thus supports the fact that most mobilomes originate from marine γ-Proteobacteria. *Shewanella* may be either the best transmitter of these mobilomes to other organisms, or simply the closest recipient. Further studies have to be done to elucidate the intrinsic donor-recipient relations between these microorganisms.

The role played by the environmental micro-flora towards the development of pathogenic microorganisms should be re-considered. Our current understanding for this process is limited to the fact that pathogenic or commensal bacteria are highly likely to acquire new virulence factors from the environment. Thus, it is generally accepted that antibiotic resistance genes which are widely spread in clinical isolates of pathogens have been generated from native genes of environmental bacteria encoding factors that aid to withstand highly toxic and polluting reagents or self-producing antibiotics [80], [88]. This study showed that the environmental microorganisms may also provide pathogens with newly generated vectors (plasmids, phages, transposons, IS-elements and integrons), which accelerate genomic rearrangements and virulence factor exchange. The efficacy of a vector is decreasing in time as bacteria acquire immunity towards it. The mechanisms through which bacteria acquire this immunity may vary from development of sophisticated anti-phage CRISPR systems to a simple fact that the remnants of the vector which may be not functional any more block the specific insertion sites in bacterial chromosome [89], [90]. HGT frequency decreases with the loss of the vector efficacy until a new vector is generated in the environmental microflora that is able to evade the acquired immunity. Appearance of a new active vector accelerates HGT that could be an explanation of the disease outbreaks that occur periodically. The vector spread oscillation is illustrated in Fig. 5 by gradient colour changes depicted in clusters of enterobacterial GIs by dark grey in cells D5-E6 and A4 to light grey in C4–D4 and intermediate shades in D3, B5 and B2. Old vectors continue their spread when new genetically naïve strains of microorganisms appear. The latter is most probably an explanation of what happened with the *E. coli* strains of group D (Fig. 4). The GIs of strains IAI39 and UMNO26 are mostly ancient inserts, only a few of their newly acquired GIs show similarity to various clusters of enterobacterial GIs. Horizontal gene exchange has driven these two phylogenetically unrelated strains [42] to share a similar phenotype and genetic markers, and as a result they were joined into group D of *E. coli*. It is quite possible that even the most primitive strains 55989 and TY-2482 of group B1 will undergo the same evolutionary process.

In this study we illustrated that the exchange of genetic materials between bacteria is not a random event but a strictly directed process with chains of donor and recipient organisms. Of all the enetrobacterial GIs analysed in our study there was not a single case of transmission which appeared to have taken place from enterobacteria to *Shewanella*. However, there are several cases where enterobacteria showed to have transmitted GIs enriched with virulence determinants to various other organisms. *Sodalis glossinidius,* a maternally transmitted intracellular endosymbiont of the tsetse flies [91] serves as one of the organisms possessing GIs which have been acquired from *Salmonella*. It was also found to comprise a type III secretion system identical to the one of *S. enterica* SPI-1 PAI [91]. A total of 41 GIs were identified in *S. glossinidius* morsitans [NC_007712] by SW Sniffer (http://anjie.bi.up.ac.za/geidb/geidb-home.php). It is rather unusual for an intracellular symbiont to possess such a number of GIs. Twelve of the 41 GIs share similarity in composition and sequence with each other and the GIs of *Salmonella*. Fig. 6B illustrates that some of the twelve (cell B5 in Fig. 5) GIs of *Sodalis* are of *Salmonella* origin. These GIs are not duplicates of the same inserts but form a clear time series of independent acquisitions depicting another example of genetic vector oscillation and implying that this intracellular organism regularly interacts with the environmental micro-flora. Acquisition of pathogenicity determinants by this organism may have influenced the spread of virulence factors and invasion of new host cells and tissues. Probably the *E. coli* strains of group B2 evolved following the same scheme upon the acquisition of virulence determinants which allowed them to access extra-intestinal tissues to cause systemic infections in humans and animals [39], .

*E. coli* strains are potential donors of PAIs to other environmental microorganisms. Few of their related inserts were found to be present in *Erwinia,* a plant pathogen which can cause septicaemia and nosocomial infections in immunologically compromised humans [92], [93]. Additional studies have to be conducted to outline bacterial species which potentially have inherited pathogenic determinants from enterobacteria.

*E. coli* TY-2482, the causative agent of a deadly outbreak in Europe in 2011, possesses a plasmid associated PAI with factors such as broad range beta-lactamase, tetracycline efflux pump and a cluster of mercury resistance genes. Ontologically these factors are not related to either of the prevalent GIs of enterobacteria shown in Fig. 4. However, this PAI is not completely novel, as there are a number of papers reporting similar inserts in rapidly evolving plasmids which are common in drug resistant strains of *Salmonella* [75], [78], [79]. OU pattern analysis indicated that there are at least two GIs in TY-2482 arising from the same origin which are also present in the genome of *E. coli* UMNO26. Although these islands were also reported to be present in *E. coli* UMNO26, they did not seem to harbour antibiotic resistance genes which are possessed by TY-2482 (Fig. 10). In contrast to gradual oscillation of genomic inserts in the genomes of enterobacteria observed in Fig. 5, these new GIs have been acquired rapidly from distant organisms (see Fig. 9, 10 and 11). Transience of the evolution and acquisition of these GIs is in consistence with the observation of a drastic rise in mercury resistant bacteria in coastal environment in India [94]. The authors reported that in 5 years from 1997 to 2003 the number of microorganisms isolated from sea water which tolerated 10 ppm of HgCl_2_ increased from none to 75% of CFU or even 95% in the polluted zones of Mumbai. It was concluded that this sharp rise in mercury tolerance could be linked to the general ocean pollution by human industrial activity. It however is plausible that the activation of old dormant vectors for horizontal gene exchange which for a long time were confined in isolated eco-niches of naturally polluted areas broke through their borders. When the concentration of heavy metal ions in ocean water reached some threshold level, the possession of heavy metal resistance plasmids became an absolute benefit for environmental microorganisms and thus these plasmids spread rapidly among bacteria and reached enterobacteria, which adopted the enormous potential of genes aiding in withstanding chemical pollutions to combat a broad range of antibiotics. But the increased tolerance to antibiotics may be the only first and most obvious problem that mankind has been faced with. These GIs have established a new channel of horizontal gene exchange for which all human commensals are naïve and hence highly vulnerable. The genetic vectors emanating from this new source may interfere with the conventional oscillation of the vectors which originated from marine γ-Proteobacteria that may cause an unpredictable cumulative impulse in the evolvement of new pathogens in the future.

There is a pressing need to create a system that will allow the monitoring of distributions of horizontally transferred GIs, to help understand and prepare for the emergence of new pathogens. An important notion from this work is that the origin of GIs cannot be determined solely by the traditional sequence similarity methods, i.e. by blast. Sequence similarity is based on functional conservations of the genes whereas the compositional similarity reflects the preferences of the replication/reparation system of the organism and its specific codon adaptation. OU pattern comparison has many limitations by itself such as OU pattern convergence between unrelated organisms and fading of OU patterns of GIs in time; thus this approach gains much more credibility when confirmed by sequence similarity comparison. Hence the combination of sequence similarity and genome linguistic approaches is synergetic in terms of discovering the donor-recipient interactions between microorganisms and timing their GI acquisitions. The analysis of the data in Table 3 showed that the heavy metal resistance genetic cassettes which are widely distributed among environmental bacteria have been acquired recently by many new microorganisms, probably resulting from general ocean pollution. The increased pollution has triggered several parallel currents of HGT. For instance, *A. baumannii* ACICU [NC_010611] has acquired its GI recently from an unknown α-Proteobacteria. The GI in *N. europaea* C91 [NC_008344:1] shows a strong compositional similarity to Firmicutes GIs harboured by *Clostridium thermocellum*, *Desulfitobacterium hafniense* and *Carboxydothermus hydrogenoformans*; however, neither of them was found to be a donor of these GIs. The GIs in *N. europaea* C91 [NC_008344:2] and *N. europaea* ATCC 19718 [NC_004757], as well as the recent inserts of metal resistance gene cassettes in *S. frigimarina* NCIMB400 [NC_008345] showed no significant compositional similarity to each other, nor to the available sequenced bacterial genomes. Each channel of HGT appears to have its own range of recipient species, which therefore may result in different consequences in terms of evolvement of new human pathogens. The effect may be even more profound if several independent fluxes of gene exchange vectors interfere in the same organism. Our further work will aim at summarizing the ontological data for newly identified GIs and highlighting the most active mainstreams of gene exchange in a scale of the whole bacterial world.

## Materials and Methods

### Source of genome sequences

Sequences of bacterial genomes were downloaded from SeqRef database. Draft sequence of TY-2482 was obtained from the FTP site: ftp://ftp.genomics.org.cn/pub/Ecoli_TY-2482.

### OU pattern statistics

In this work DNA sequences were compared by OU patterns. OU pattern was denoted as a matrix of deviations *Δ_w_* of observed from expected counts for all possible words of length *N*. Tetranucleotide usage patterns were used in this work, thus *N* = 256. Oligonucleotides or words are distributed in sequences logarithmically and the deviations of their frequencies from expectations may be found as follows: 
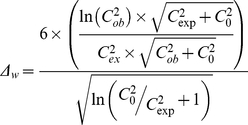
(1)


where *C_ob_* is the observed count of a word *w*; *C_exp_* is its expected count and *C_0_* is a null hypothesis expectation estimated from the assumption of an equal distribution of words in the sequence: (*C_0_ = L_seq_×4^-N^*).

Expected counts of words *C_e_* were calculated by taking into account the frequencies of constituent mononucleotides in the sequence by 0-order Markov model [14].

The distance *D* between two patterns was calculated as the sum of absolute distances between ranks of identical words (*w*, in a total *4^N^* different words) after ordering of words by *Δ_w_* values (equation 1) in patterns *i* and *j* as follows: 
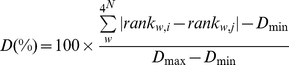
(2)


Compositional sequence similarity was denoted as 100% – D.

PS is a particular case of D where patterns *i* and *j* are calculated for the same DNA but for direct and reversed strands, respectively. *D_max_ = 4^N^×(4^N^ – 1)/2* and *D_min_ = 0* when calculating a D, or, in the case of PS calculation, *D_min_ = 0* if *N* is an odd number, or *D_min_ = 4^N^ – 2^N^* if *N* is an even number due to the presence of palindromic words. Normalization of D-values by *D_max_* ensures that the distances between two sequences are comparable regardless of the word length.

Relative variance of an OU pattern was calculated by the following equation: 
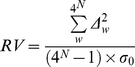
(3)


where *N* is word length; *Δ^2^_w_* is the square of a word *w* count deviation (see equation 1); and *σ_0_* is the expected standard deviation of the word distribution in a randomly generated sequence which depends on the sequence length (*L_seq_*) and the word length (*N*): 
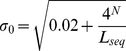
(4)


GRV is a particular case of RV when expected counts of words are calculated based on the frequencies of nucleotides estimated for the complete genome [14], [18], [22].

### Inferring donor-recipient relations

To predict donor-recipient relations between bacterial genomes, GIs of the organisms were projected onto a two-dimensional plot as follows: 
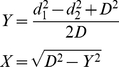
(5)


where d_1_ is the distance between GI and the host chromosome (equation 1); d_2_ – distance between GI and the counterpart chromosome; and D – distance between chromosomal OU patterns (all these distances were denoted in Fig. 6). Note, that plotting of two GIs from two different genomes by equation 5 makes sense only if the similarity between GIs is high (>75% in this study), or at least if two GIs may be linked by intermediate nodes.

### Graphical representation of GI clusters

Graphs generated in this study were calculated by the *circo* algorithms of Graphviz distributed under the terms of the Eclipse Public Licence [http://www.graphviz.org/].

### Sequence similarity comparison

Local blast was performed by using NCBI blastall.exe and bl2seq.exe utilities.

### Markov Clustering Algorithm

Protein coding sequences located in GIs were clustered using Markov Clustering Algorithm (MCL) introduced by van Dongen as a graph-based, deterministic, partitional algorithm incorporating hard clustering, where flow or movement in a graph is simulated by algebraic methods [95]. MCL was used with an inflation parameter set to 1.8. Similarity scores for creating clusters were obtained with an initial all-against-all blastp and an e-value cut-off of 0.0001. The MCL requires a degree of relatedness for clustering, thus bit-scores were deemed a favourable indication of similarity. The MCL preformed sufficiently and it consequently clustered the coding sequences into clearly defined classes. Co-occurrence of genes from different clusters in the same GIs was checked by Chi-square test [96]. SIG values of the functional similarity between predicted gene modules was calculated as described previously [73].

### Programming and availability

All in-house programs were written in Python 2.5. SeqWord Sniffer, databases of predicted GIs and interactive maps of GI clusters are available at the SeqWord project web-site www.bi.up.ac.za/SeqWord/. At the time of writing this paper the interactive maps were implemented only as SVG graphs, which are compatible and tested only on Mozilla Firefox 6.0. A JPG converter for the users of other browsers is under development.

## Supporting Information

Table S1 **Detection of known pathogenicity genomic islands from PAI DB by different programs.**
(PDF)

Table S2 **Numbers of genomic islands identified by different programs.**
(PDF)

Table S3 **Confirmation of the SW Sniffer true positive predictions by IslandViewer programs.**
(PDF)
