# Supplementary material for: Mainstreams of Horizontal Gene Exchange in Enterobacteria: Consideration of the Outbreak of Enterohemorrhagic E. coli O104:H4 in Germany in 2011
Source: PLoS One. 2011 Oct 14;6(10):e25702. doi: 10.1371/journal.pone.0025702 (PMC3195076; doi:10.1371/journal.pone.0025702)
Supplement: Table S1 — Detection of known pathogenicity genomic islands from PAI DB by different programs. (PDF) [file pone.0025702.s001.pdf]

Tabel S1. Detection of known pathogenicity genomic islands from PAI DB by different programs

Genomes	#	PAI Name	Start	Stop	IslandPick	SIGI-HMM	IslandPath	SW Sniffer
NC_004668, Enterococcus faecalis V583	1	Not named	445282	582786	0	1	1	1
NC_004431, Escherichia coli CFT073	1	PAI I CFT073	3406225	3450866	0	1	1	1
	2	PAI II CFT073	4913367	4971660	0	0	1	1
NC_002655, Escherichia coli O157:H7 EDL933	1	LEE	4649752	4692545	0	1	1	1
NC_002695, Escherichia coli O157:H7 Sakai	1	LEE	4580769	4623562	0	1	1	1
NC_006570, Francisella tularensis subsp. tularensis Schu 4	1	FPI	1374701	1408279	0	0	0	1
	2	FPI	1768045	1801623	0	0	0	1
NC_004917, Helicobacter hepaticus ATCC 51449	1	HHGI1	223218	294244	0	0	0	1
NC_000915, Helicobacter pylori 26695	1	cag PAI	547328	585350	0	0	0	1
NC_000921, Helicobacter pylori J99	1	cag PAI	510500	548694	0	0	0	1
NC_003112, Neisseria meningitidis MC58	1	IHT-A	75694	109777	0	0	0	1
	2	IHT-C	1827738	1860290	0	1	1	1
NC_004578, Pseudomonas syringae pv. tomato DC3000	1	Hrp PAI	1502395	1551861	0	0	0	0
NC_006905, Salmonella enterica subsp. enterica serovar Choleraesuis str. SC-B67	1	SPI-5	1155936	1161624	0	0	0	0
	2	SPI-11	1350481	1366166	0	1	0	1
	3	SPI-2	1497670	1539498	0	1	0	1
	4	SPI-12	2354604	2365678	0	1	0	1
	5	SPI-1	2960260	3003747	0	1	0	1
	6	SPI-3	3890879	3903697	0	0	1	0
	7	SPI-4	4411902	4438599	0	1	0	1
NC_003198, Salmonella enterica subsp. enterica serovar Typhi CT18	1	SPI-6	302092	360757	0	1	0	1
	2	SPI-5	1085068	1092563	0	0	0	1
	3	SPI-2	1624920	1666524	0	1	0	1
	4	SPI-9	2743495	2759190	0	0	0	0
	5	SPI-1	2858736	2900586	0	1	0	1
	6	SPI-8	3132530	3139414	0	1	0	1
	7	SPI-3	3883613	3900553	0	0	0	1
	8	SPI-4	4322993	4346383	0	1	0	1
	9	SPI-7	4409511	4543148	1	1	1	1
	10	SPI-10	4683605	4716538	1	1	0	1
NC_004631, Salmonella enterica subsp. enterica serovar Typhi Ty2	1	SPI-2	1314607	1356216	0	1	0	1
	2	SPI-1	2844593	2886443	0	1	0	1
	3	SPI-7	4394302	4526050	1	1	1	1
NC_003197, Salmonella typhimurium LT2	1	SPI-5	1175321	1184389	0	0	0	1
	2	SPI-2	1461740	1501800	0	1	0	1
	3	SPI-1	3005842	3050120	0	1	0	1
	4	SPI-3	3948576	3965191	0	0	0	0
	5	SPI-4	4477849	4501259	0	1	0	1
NC_002505, Vibrio cholerae O1 biovar eltor str. N16961	1	VSP-I	175343	189380	0	1	1	1
	2	VSP-II	523156	530602	0	1	1	1
	3	VPI	873242	914124	0	1	1	1
	4	VPI-2	1895692	1952861	0	1	1	1
NC_003919, Xanthomonas axonopodis pv. citri str. 306	1	Hrp PAI	462225	488334	0	0	0	1
NC_007086, Xanthomonas campestris pv. campestris str. 8004	1	Hrp PAI	3596348	3619441	0	1	0	1
NC_003902, Xanthomonas campestris pv. campestris str. ATCC 33913	1	Hrp PAI	1424335	1447429	0	1	0	1
NC_007508, Xanthomonas campestris pv. vesicatoria str. 85-10	1	Hrp PAI	459479	494176	0	1	0	1
NC_006834, Xanthomonas oryzae pv. oryzae KACC10331	1	Hrp PAI	58273	90451	0	0	1	0
NC_003143, Yersinia pestis CO92	1	HPI	2134947	2171362	0	0	1	1
NC_004088, Yersinia pestis KIM	1	HPI	2645436	2681339	0	0	1	1
NC_005810, Yersinia pestis biovar Medievalis str. 91001	1	HPI	1799736	1828106	0	0	0	1
NC_006155, Yersinia pseudotuberculosis IP32953	1	HPI	1913940	1949837	0	0	0	1
**Rate of false negatives**					94.12%	41.18%	68.63%	11.76%
