# Supplementary material for: Mainstreams of Horizontal Gene Exchange in Enterobacteria: Consideration of the Outbreak of Enterohemorrhagic E. coli O104:H4 in Germany in 2011
Source: PLoS One. 2011 Oct 14;6(10):e25702. doi: 10.1371/journal.pone.0025702 (PMC3195076; doi:10.1371/journal.pone.0025702)
Supplement: Table S2 — Numbers of genomic islands identified by different programs. (PDF) [file pone.0025702.s002.pdf]

Table S2. Numbers of genomic islands identified by different programs

#	Genome	In total				Unconfirmed predictions*			
		IslandPick	SIGI-HMM	IslandPath	SW Sniffer	IslandPick	SIGI-HMM	IslandPath	SW Sniffer
1	Acidovorax avenae subsp. citrulli AAC00-1 [NC_008752]	0	35	18	32	0	10	5	9
2	Acidovorax sp. JS42 [NC_008782]	25	34	11	27	7	7	1	9
3	Acinetobacter baumannii ATCC 17978 [NC_009085]	4	4	4	8	1	3	2	6
4	Anaeromyxobacter dehalogenans 2CP-C [NC_007760]	7	18	2	21	3	4	0	11
5	Arthrobacter sp. FB24 chr. 1 [NC_008541]	0	12	3	16	0	2	1	8
6	Azoarcus sp. EbN1 [NC_006513]	0	22	24	15	0	2	11	4
7	Bacillus anthracis str. 'Ames Ancestor' [NC_007530]	3	0	5	38	0	0	3	34
8	Bacillus anthracis str. Ames [NC_003997]	3	0	5	35	0	0	3	31
9	Bacillus anthracis str. Sterne [NC_005945]	3	0	5	41	0	0	3	37
10	Bacillus cereus ATCC 10987 [NC_003909]	3	4	10	30	1	0	5	26
11	Bacillus cereus ATCC 14579 [NC_004722]	5	2	9	32	0	2	6	29
12	Bacillus cereus E33L [NC_006274]	0	1	5	33	0	0	4	33
13	Bacillus licheniformis ATCC 14580 [NC_006322]	0	3	8	27	0	0	0	16
14	Bacillus thuringiensis serovar konkukian str. 97-27 [NC_005957]	0	1	7	34	0	1	7	34
15	Bacillus thuringiensis str. Al Hakam [NC_008600]	2	2	5	29	0	1	2	27
16	Bordetella bronchiseptica RB50 [NC_002927]	0	25	6	16	0	8	0	5
17	Bordetella parapertussis 12822 [NC_002928]	0	11	2	8	0	4	1	4
18	Bordetella pertussis Tohama I [NC_002929]	0	11	18	7	0	4	12	3
19	Borrelia afzelii PKo [NC_008277]	0	8	0	4	0	8	0	4
20	Borrelia burgdorferi B31 [NC_001318]	0	3	0	0	0	3	0	0
21	Borrelia garinii PBi chr. linear [NC_006156]	0	6	0	6	0	6	0	6
22	Borrelia turicatae 91E135 [NC_008710]	0	24	0	10	0	21	0	8
23	Bradyrhizobium japonicum USDA 110 [NC_004463]	0	71	36	27	0	12	8	1
24	Burkholderia cenocepacia AU 1054 chr. 1 [NC_008060]	10	10	10	13	7	1	1	3
25	Burkholderia cenocepacia AU 1054 chr. 2 [NC_008061]	1	14	9	11	0	1	1	1
26	Burkholderia cenocepacia AU 1054 chr. 3 [NC_008062]	0	1	3	3	0	0	3	2
27	Burkholderia cenocepacia HI2424 chr. 1 [NC_008542]	2	12	9	10	0	1	2	3
28	Burkholderia cenocepacia HI2424 chr. 2 [NC_008543]	1	16	8	13	1	2	1	2
29	Burkholderia cenocepacia HI2424 chr. 3 [NC_008544]	1	2	3	5	0	0	2	3
30	Burkholderia cepacia AMMD chr. 1 [NC_008390]	1	15	5	14	0	0	0	3
31	Burkholderia cepacia AMMD chr. 2 [NC_008391]	1	13	2	12	0	3	1	2
32	Burkholderia cepacia AMMD chr. 3 [NC_008392]	7	8	2	4	6	3	2	2
33	Burkholderia mallei ATCC 23344 chr. 1 [NC_006348]	0	2	16	5	0	0	13	1
34	Burkholderia mallei ATCC 23344 chr. 2 [NC_006349]	0	3	17	5	0	1	14	2
35	Burkholderia mallei NCTC 10229 chr. I [NC_008835]	0	3	14	5	0	0	9	1
36	Burkholderia mallei NCTC 10229 chr. II [NC_008836]	0	4	18	4	0	0	15	1
37	Burkholderia mallei NCTC 10247 chr. I [NC_009079]	0	2	15	6	0	0	10	2
38	Burkholderia mallei NCTC 10247 chr. II [NC_009080]	1	4	19	4	0	0	15	2
39	Burkholderia mallei SAVP1 chr. I [NC_008784]	0	1	10	3	0	0	9	2
40	Burkholderia mallei SAVP1 chr. II [NC_008785]	0	4	20	5	0	0	17	2
41	Burkholderia pseudomallei 1106a chr. I [NC_009076]	2	17	10	16	0	2	1	3
42	Burkholderia pseudomallei 1106a chr. II [NC_009078]	4	11	8	10	2	1	3	3
43	Burkholderia pseudomallei 1710b chr. I [NC_007434]	7	25	9	18	0	4	0	3
44	Burkholderia pseudomallei 1710b chr. II [NC_007435]	5	13	6	11	1	5	1	4
45	Burkholderia pseudomallei 668 chr. I [NC_009074]	3	14	12	15	1	1	2	3
46	Burkholderia pseudomallei 668 chr. II [NC_009075]	1	10	7	9	1	2	2	3
47	Burkholderia pseudomallei K96243 chr. 1 [NC_006350]	2	19	10	15	1	3	0	3
48	Burkholderia pseudomallei K96243 chr. 2 [NC_006351]	3	6	4	11	1	1	0	6
49	Burkholderia sp. 383 chr. 1 [NC_007510]	5	12	2	14	1	1	0	4
50	Burkholderia sp. 383 chr. 2 [NC_007511]	0	11	2	12	0	2	0	3
51	Burkholderia sp. 383 chr. 3 [NC_007509]	0	13	0	9	0	5	0	3
52	Burkholderia thailandensis E264 chr. I [NC_007651]	5	12	14	13	0	0	1	0
53	Burkholderia thailandensis E264 chr. II [NC_007650]	8	0	9	8	6	0	4	3
54	Burkholderia xenovorans LB400 chr. 1 [NC_007951]	0	38	18	22	0	6	2	4
55	Burkholderia xenovorans LB400 chr. 2 [NC_007952]	0	15	11	14	0	2	4	3
56	Burkholderia xenovorans LB400 chr. 3 [NC_007953]	0	4	3	9	0	0	2	5
57	Campylobacter fetus subsp. fetus 82-40 [NC_008599]	0	1	2	8	0	0	2	7
58	Campylobacter jejuni RM1221 [NC_003912]	5	0	3	8	0	0	0	6
59	Candidatus Protochlamydia amoebophila UWE25 [NC_005861]	0	1	6	5	0	0	5	3
60	Caulobacter crescentus CB15 [NC_002696]	0	14	7	4	0	10	5	1
61	Clostridium acetobutylicum ATCC 824 [NC_003030]	0	2	6	23	0	1	5	23
62	Clostridium difficile 630 [NC_009089]	0	14	7	22	0	6	0	13
63	Ehrlichia canis str. Jake [NC_007354]	0	20	0	10	0	17	0	7
64	Ehrlichia chaffeensis str. Arkansas [NC_007799]	0	13	0	12	0	12	0	11
65	Ehrlichia ruminantium str. Gardel [NC_006831]	0	11	0	16	0	10	0	15
66	Ehrlichia ruminantium str. Welgevonden [NC_005295]	0	12	0	0	0	12	0	0
67	Ehrlichia ruminantium str. Welgevonden [NC_006832]	0	10	0	15	0	10	0	15
68	Erythrobacter litoralis HTCC2594 [NC_007722]	0	11	8	6	0	3	2	1
69	Francisella tularensis subsp. holarctica [NC_007880]	0	0	9	15	0	0	7	13
70	Frankia alni ACN14a [NC_008278]	0	13	11	38	0	6	1	23
71	Frankia sp. CcI3 [NC_007777]	0	14	25	25	0	3	12	6
72	Geobacillus kaustophilus HTA426 [NC_006510]	4	31	16	28	0	1	0	6
73	Gramella forsetii KT0803 [NC_008571]	0	3	8	15	0	1	6	14
74	Haemophilus ducreyi 35000HP [NC_002940]	0	3	4	10	0	2	4	9
75	Haemophilus influenzae 86-028NP [NC_007146]	3	4	3	7	2	0	1	6
76	Haemophilus somnus 129PT [NC_008309]	2	0	5	16	1	0	3	15
77	Halobacterium sp. NRC-1 [NC_002607]	0	8	6	0	0	2	2	0
78	Halorhodospira halophila SL1 [NC_008789]	0	11	6	12	0	0	1	5
79	Helicobacter acinonychis str. Sheeba [NC_008229]	6	0	4	3	3	0	3	1
80	Hyphomonas neptunium ATCC 15444 [NC_008358]	0	11	3	9	0	2	1	3
81	Lactobacillus johnsonii NCC 533 [NC_005362]	8	0	4	7	4	0	3	5
82	Lactococcus lactis subsp. cremoris MG1363 [NC_009004]	3	6	16	10	0	1	9	6
83	Lactococcus lactis subsp. cremoris SK11 [NC_008527]	3	1	13	11	1	0	11	7
84	Lactococcus lactis subsp. lactis Il1403 [NC_002662]	1	1	11	9	0	0	9	7
85	Legionella pneumophila str. Lens [NC_006369]	1	1	5	7	1	0	5	6
86	Legionella pneumophila subsp. pneumophila str. Philadelphia 1 [NC_002942]	4	1	3	7	3	0	2	6

87 Leifsonia xyli subsp. xyli str. CTCB07 [NC_006087]	0	19	11	18	0	3	1	5
88 Leptospira interrogans serovar Copenhageni str. Fiocruz L1-130 chr. I [NC_005823]	0	0	10	8	0	0	7	5
89 Leptospira interrogans serovar Lai str. 56601 chr. I [NC_004342]	0	0	15	9	0	0	23	4
90 Magnetospirillum magneticum AMB-1 [NC_007626]	0	48	15	20	0	13	3	0
91 Maricaulis maris MCS10 [NC_008347]	0	8	6	7	0	1	0	2
92 Mesorhizobium loti MAFF303099 [NC_002678]	0	40	17	17	0	16	5	1
93 Methylobacillus flagellatus KT [NC_007947]	0	9	3	13	0	5	2	11
94 Mycobacterium avium 104 [NC_008595]	6	26	13	21	1	6	6	6
95 Mycobacterium avium subsp. paratuberculosis K-10 [NC_002944]	3	16	9	12	0	6	6	3
96 Mycobacterium bovis AF2122/97 [NC_002945]	0	4	7	12	0	1	4	6
97 Mycobacterium bovis BCG str. Pasteur 1173P2 [NC_008769]	0	4	6	11	0	1	3	5
98 Mycobacterium smegmatis str. MC2 155 [NC_008596]	0	33	17	14	0	13	7	3
99 Mycobacterium sp. JLS [NC_009077]	0	29	16	16	0	6	4	2
100 Mycobacterium sp. KMS [NC_008705]	0	27	19	17	0	7	5	3
101 Mycobacterium sp. MCS [NC_008146]	0	23	20	14	0	7	8	3
102 Mycobacterium ulcerans Agy99 [NC_008611]	1	10	23	4	0	6	18	2
103 Mycobacterium vanbaalenii PYR-1 [NC_008726]	0	29	20	18	0	7	8	4
104 Myxococcus xanthus DK 1622 [NC_008095]	0	0	19	48	0	0	7	34
105 Neisseria meningitidis Z2491 [NC_003116]	1	6	2	15	1	1	0	9
106 Nitrobacter hamburgensis X14 [NC_007964]	0	18	15	7	0	10	9	3
107 Nitrobacter winogradskyi Nb-255 [NC_007406]	0	4	25	4	0	0	20	1
108 Nocardia farcinica IFM 10152 [NC_006361]	0	0	12	23	0	0	5	17
109 Nocardioides sp. JS614 [NC_008699]	0	24	14	20	0	4	0	7
110 Nostoc sp. PCC 7120 [NC_003272]	18	1	9	14	14	1	5	9
111 Novosphingobium aromaticivorans DSM 12444 [NC_007794]	0	9	5	7	0	3	2	2
112 Paracoccus denitrificans PD1222 chr. 1 [NC_008686]	0	22	5	11	0	6	0	0
113 Paracoccus denitrificans PD1222 chr. 2 [NC_008687]	0	8	5	7	0	3	1	3
114 Photobacterium profundum SS9 chr. 2 [NC_006371]	0	17	9	5	0	14	6	2
115 Polaromonas sp. JS666 [NC_007948]	0	23	10	13	0	11	4	4
116 Pyrobaculum islandicum DSM 4184 [NC_008701]	0	0	8	15	0	0	3	11
117 Pyrococcus furiosus DSM 3638 [NC_003413]	0	4	4	18	0	0	0	14
118 Ralstonia eutropha H16 chr. 1 [NC_008313]	0	35	4	14	0	21	0	2
119 Ralstonia eutropha H16 chr. 2 [NC_008314]	0	24	3	13	0	11	0	3
120 Ralstonia eutropha JMP134 chr. 1 [NC_007347]	0	19	4	12	0	8	1	3
121 Ralstonia eutropha JMP134 chr. 2 [NC_007348]	0	9	1	8	0	3	0	3
122 Ralstonia metallidurans CH34 chr. 2 [NC_007974]	0	11	3	12	0	0	0	7
123 Ralstonia solanacearum GMI1000 [NC_003295]	0	29	8	19	0	6	0	3
124 Rhodobacter sphaeroides 2.4.1 chr. 1 [NC_007493]	0	5	7	6	0	2	2	1
125 Rhodobacter sphaeroides 2.4.1 chr. 2 [NC_007494]	0	4	3	6	0	0	0	1
126 Rhodobacter sphaeroides ATCC 17029 chr. 1 [NC_009049]	0	5	4	5	0	0	0	0
127 Rhodobacter sphaeroides ATCC 17029 chr. 2 [NC_009050]	0	6	4	5	0	0	1	2
128 Rhodococcus sp. RHA1 [NC_008268]	0	8	25	20	0	4	11	11
129 Rhodopseudomonas palustris BisA53 [NC_008435]	0	19	9	9	0	2	3	3
130 Rhodopseudomonas palustris BisB18 [NC_007925]	0	13	9	10	0	5	4	2
131 Rhodopseudomonas palustris BisB5 [NC_007958]	12	10	7	7	3	1	1	1
132 Rhodopseudomonas palustris CGA009 [NC_005296]	0	9	6	5	0	5	5	3
133 Rhodopseudomonas palustris HaA2 [NC_007778]	20	16	7	7	12	4	1	1
134 Rhodospirillum rubrum ATCC 11170 [NC_007643]	0	9	4	10	0	4	2	4
135 Rubrobacter xylanophilus DSM 9941 [NC_008148]	0	11	5	13	0	0	1	5
136 Saccharopolyspora erythraea NRRL 2338 [NC_009142]	0	18	18	33	0	1	3	17
137 Silicibacter pomeroyi DSS-3 [NC_003911]	0	13	7	14	0	0	0	4
138 Sinorhizobium meliloti 1021 [NC_003047]	7	6	5	7	2	0	2	2
139 Sphingopyxis alaskensis RB2256 [NC_008048]	0	17	8	2	0	8	2	1
140 Staphylococcus aureus RF122 [NC_007622]	0	1	5	19	0	0	3	17
141 Staphylococcus aureus subsp. aureus MRSA252 [NC_002952]	0	3	10	19	0	2	6	15
142 Staphylococcus aureus subsp. aureus Mu50 [NC_002758]	1	4	7	19	0	1	2	16
143 Staphylococcus aureus subsp. aureus N315 [NC_002745]	0	4	8	18	0	2	4	16
144 Staphylococcus epidermidis ATCC 12228 [NC_004461]	0	3	7	19	0	1	5	16
145 Staphylococcus haemolyticus JCSC1435 [NC_007168]	0	0	10	19	0	0	8	17
146 Streptococcus agalactiae 2603V/R [NC_004116]	1	10	4	10	0	5	1	7
147 Streptomyces avermitilis MA-4680 [NC_003155]	0	11	20	37	0	2	7	24
148 Streptomyces coelicolor A3(2) [NC_003888]	0	29	17	44	0	4	3	21
149 Sulfolobus solfataricus P2 [NC_002754]	12	6	24	27	7	0	10	19
150 Sulfolobus tokodaii str. 7 [NC_003106]	0	14	7	25	0	6	2	18
151 Symbiobacterium thermophilum IAM 14863 [NC_006177]	0	10	13	17	0	0	1	9
152 Synechococcus sp. CC9311 [NC_008319]	0	15	1	26	0	0	0	14
153 Synechococcus sp. CC9605 [NC_007516]	0	29	6	26	0	2	1	4
154 Synechococcus sp. CC9902 [NC_007513]	0	11	1	16	0	1	0	7
155 Synechococcus sp. WH 8102 [NC_005070]	0	23	4	19	0	3	0	4
156 Thermosynechococcus elongatus BP-1 [NC_004113]	0	2	12	5	0	1	10	2
157 Thiobacillus denitrificans ATCC 25259 [NC_007404]	0	20	10	13	0	3	1	2
158 Thiomicrospira crunogena XCL-2 [NC_007520]	0	3	7	7	0	0	4	5
159 Verminephrobacter eiseniae EF01-2 [NC_008786]	0	48	15	36	0	13	6	7
160 Vibrio cholerae O1 biovar eltor str. N16961 chr. II [NC_002506]	0	7	5	0	0	1	0	0
161 Vibrio parahaemolyticus RIMD 2210633 chr. I [NC_004603]	8	16	7	16	3	6	1	6
162 Xanthomonas axonopodis pv. citri str. 306 [NC_003919]	6	24	15	22	2	2	2	7
163 Xanthomonas campestris pv. campestris str. 8004 [NC_007086]	6	34	9	23	1	6	0	3
164 Xanthomonas campestris pv. campestris str. ATCC 33913 [NC_003902]	4	36	10	27	1	5	0	5
165 Xanthomonas campestris pv. vesicatoria str. 85-10 [NC_007508]	6	31	24	29	1	5	2	7
166 Xanthomonas oryzae pv. oryzae KACC10331 [NC_006834]	11	8	23	9	6	1	15	3
167 Xanthomonas oryzae pv. oryzae MAFF 311018 [NC_007705]	11	10	27	8	6	3	21	3
168 Xylella fastidiosa 9a5c [NC_002488]	3	21	2	0	0	16	0	0
169 Xylella fastidiosa Temecula1 [NC_004556]	0	15	0	13	0	12	0	10
**Total number of predicted and unconfirmed Gis**	**302**	**2053**	**1509**	**2425**	**115**	**583**	**636**	**1216**
**Percentage of unconfirmed Gis**					**38.08%**	**28.40%**	**42.15%**	**50.14%**

* Unconfirmed prediction means that the GI was detected only by one of four programs
