# Supplementary material for: Mainstreams of Horizontal Gene Exchange in Enterobacteria: Consideration of the Outbreak of Enterohemorrhagic E. coli O104:H4 in Germany in 2011
Source: PLoS One. 2011 Oct 14;6(10):e25702. doi: 10.1371/journal.pone.0025702 (PMC3195076; doi:10.1371/journal.pone.0025702)
Supplement: Table S3 — Confirmation of the SW Sniffer true positive predictions by IslandViewer programs. (PDF) [file pone.0025702.s003.pdf]

Table S3. Confirmation of the SW Sniffer true positive predictions by IslandViewer programs.

Genomic Islands predicted by SW Sniffer: NC_accession|Strain|[coordinates]	IslandPick	SIGI-HMM	IslandPath	Jointly
NC_000854:9|Aeropyrum pernix K1 chromosome I [727300-748849]	0	1	0	1
NC_000868:1|Pyrococcus abyssi GE5 chromosome I [1141250-1150799]	0	0	0	0
NC_000868:2|Pyrococcus abyssi GE5 chromosome I [1614650-1627399]	0	0	0	0
NC_000907:1|Haemophilus influenzae Rd KW20 [917150-926699]	0	0	0	0
NC_000913:11|Escherichia coli K12-MG1655 chromosome I [1086600-1095199]	0	1	0	1
NC_000913:12|Escherichia coli K12-MG1655 chromosome I [1098600-1104999]	0	0	0	0
NC_000913:14|Escherichia coli K12-MG1655 chromosome I [1205450-1222299]	0	1	1	1
NC_000913:15|Escherichia coli K12-MG1655 chromosome I [1416850-1424699]	0	1	1	1
NC_000913:16|Escherichia coli K12-MG1655 chromosome I [1428000-1434049]	0	0	0	0
NC_000913:17|Escherichia coli K12-MG1655 chromosome I [1523800-1530999]	0	0	0	0
NC_000913:19|Escherichia coli K12-MG1655 chromosome I [1571950-1588149]	0	1	0	1
NC_000913:20|Escherichia coli K12-MG1655 chromosome I [1629150-1643899]	0	1	0	1
NC_000913:22|Escherichia coli K12-MG1655 chromosome I [2030850-2043349]	0	0	0	0
NC_000913:23|Escherichia coli K12-MG1655 chromosome I [2048800-2057749]	0	1	0	1
NC_000913:24|Escherichia coli K12-MG1655 chromosome I [2096550-2110049]	0	1	1	1
NC_000913:25|Escherichia coli K12-MG1655 chromosome I [2183050-2190049]	0	1	0	1
NC_000913:26|Escherichia coli K12-MG1655 chromosome I [2378800-2386449]	0	1	0	1
NC_000913:27|Escherichia coli K12-MG1655 chromosome I [2458800-2470399]	0	1	1	1
NC_000913:28|Escherichia coli K12-MG1655 chromosome I [2476350-2492049]	0	1	0	1
NC_000913:31|Escherichia coli K12-MG1655 chromosome I [2776900-2786499]	0	1	0	1
NC_000913:32|Escherichia coli K12-MG1655 chromosome I [2876350-2883849]	0	1	0	1
NC_000913:33|Escherichia coli K12-MG1655 chromosome I [2981400-2995799]	0	0	0	0
NC_000913:35|Escherichia coli K12-MG1655 chromosome I [3261200-3267549]	0	0	0	0
NC_000913:37|Escherichia coli K12-MG1655 chromosome I [3626650-3634149]	0	0	0	0
NC_000913:40|Escherichia coli K12-MG1655 chromosome I [3790250-3804849]	0	1	0	1
NC_000913:41|Escherichia coli K12-MG1655 chromosome I [4471550-4477549]	0	1	0	1
NC_000913:42|Escherichia coli K12-MG1655 chromosome I [4497150-4505199]	0	1	0	1
NC_000913:43|Escherichia coli K12-MG1655 chromosome I [4531900-4542599]	0	1	0	1
NC_000913:44|Escherichia coli K12-MG1655 chromosome I [4569800-4577849]	0	1	0	1
NC_000913:9|Escherichia coli K12-MG1655 chromosome I [732150-737849]	0	0	0	0
NC_000915:1|Helicobacter pylori 26695 chromosome I [1052950-1064599]	0	0	0	0
NC_000916:2|Methanothermobacter thermautotrophicus Delta H chromosome I [255800-260899]	0	0	0	0
NC_000916:3|Methanothermobacter thermautotrophicus Delta H chromosome I [259850-263899]	0	0	0	0
NC_000916:4|Methanothermobacter thermautotrophicus Delta H chromosome I [264850-278299]	0	1	0	1
NC_000916:8|Methanothermobacter thermautotrophicus Delta H chromosome I [831900-841799]	0	0	0	0
NC_000917:1|Archaeoglobus fulgidus DSM4304 chromosome I [43700-51099]	0	0	0	0
NC_000917:6|Archaeoglobus fulgidus DSM4304 chromosome I [534550-556249]	0	1	0	1
NC_000917:7|Archaeoglobus fulgidus DSM4304 chromosome I [797600-804399]	0	0	0	0
NC_000918:1|Aquifex aeolicus VF5 chromosome I [249450-263299]	0	1	0	1
NC_000918:2|Aquifex aeolicus VF5 chromosome I [340650-352449]	0	0	0	0
NC_000918:5|Aquifex aeolicus VF5 chromosome I [761200-766299]	0	0	0	0
NC_000921:1|Helicobacter pylori J99 chromosome I [1041450-1058499]	1	0	0	1
NC_000961:1|Pyrococcus horikoshii OT3 chromosome I [381050-385849]	0	0	0	0
NC_000962:5|Mycobacterium tuberculosis H37Rv chromosome I [1688850-1698099]	0	0	0	0
NC_000962:6|Mycobacterium tuberculosis H37Rv chromosome I [2610900-2614949]	0	0	0	0
NC_000962:8|Mycobacterium tuberculosis H37Rv chromosome I [3120950-3130549]	0	0	0	0
NC_000964:10|Bacillus subtilis 168 chromosome I [2052000-2072849]	0	1	0	1
NC_000964:11|Bacillus subtilis 168 chromosome I [2148850-2249149]	0	1	1	1
NC_000964:12|Bacillus subtilis 168 chromosome I [2250550-2284399]	0	1	0	1
NC_000964:13|Bacillus subtilis 168 chromosome I [2652400-2659749]	0	0	1	1
NC_000964:14|Bacillus subtilis 168 chromosome I [2706650-2726699]	0	0	0	0
NC_000964:15|Bacillus subtilis 168 chromosome I [2727300-2735799]	0	0	0	0
NC_000964:16|Bacillus subtilis 168 chromosome I [3664350-3673149]	0	1	0	1
NC_000964:17|Bacillus subtilis 168 chromosome I [3673200-3682349]	0	0	0	0
NC_000964:18|Bacillus subtilis 168 chromosome I [4120600-4133299]	0	1	1	1
NC_000964:19|Bacillus subtilis 168 chromosome I [4168350-4175549]	0	0	0	0
NC_000964:4|Bacillus subtilis 168 chromosome I [737400-745299]	0	0	1	1
NC_000964:5|Bacillus subtilis 168 chromosome I [1123300-1128849]	0	1	0	1
NC_000964:7|Bacillus subtilis 168 chromosome I [1477600-1481349]	0	0	0	0
NC_000964:8|Bacillus subtilis 168 chromosome I [1876550-1888099]	0	0	0	0
NC_000964:9|Bacillus subtilis 168 chromosome I [1896100-1904299]	0	0	0	0
NC_002570:1|Bacillus halodurans C-125 chromosome I [3194100-3200949]	0	0	0	0
NC_002570:2|Bacillus halodurans C-125 chromosome I [3830200-3840049]	0	1	0	1
NC_002570:3|Bacillus halodurans C-125 chromosome I [4122000-4144299]	0	1	1	1
NC_002608:3|Halobacterium sp. NRC-1 plasmid pNRC200 [106850-110499]	0	0	0	0
NC_002655:1|Escherichia coli O157-H7 EDL933 chromosome I [15150-24999]	0	1	0	1
NC_002655:11|Escherichia coli O157-H7 EDL933 chromosome I [1071750-1077749]	0	1	0	1
NC_002655:13|Escherichia coli O157-H7 EDL933 chromosome I [1336300-1343799]	0	1	0	1
NC_002655:14|Escherichia coli O157-H7 EDL933 chromosome I [1415700-1426949]	0	0	0	0
NC_002655:15|Escherichia coli O157-H7 EDL933 chromosome I [1427950-1433399]	0	0	0	0
NC_002655:16|Escherichia coli O157-H7 EDL933 chromosome I [1451350-1463399]	1	1	1	1
NC_002655:17|Escherichia coli O157-H7 EDL933 chromosome I [1467350-1473549]	1	1	0	1
NC_002655:18|Escherichia coli O157-H7 EDL933 chromosome I [1500450-1513149]	0	1	0	1
NC_002655:19|Escherichia coli O157-H7 EDL933 chromosome I [1659300-1671749]	0	1	1	1
NC_002655:20|Escherichia coli O157-H7 EDL933 chromosome I [1701800-1709649]	0	1	0	1
NC_002655:24|Escherichia coli O157-H7 EDL933 chromosome I [1977400-1990399]	0	1	0	1
NC_002655:25|Escherichia coli O157-H7 EDL933 chromosome I [2017700-2024649]	0	0	0	0
NC_002655:27|Escherichia coli O157-H7 EDL933 chromosome I [2145000-2153799]	0	1	0	1
NC_002655:3|Escherichia coli O157-H7 EDL933 chromosome I [303700-317399]	0	1	1	1
NC_002655:31|Escherichia coli O157-H7 EDL933 chromosome I [2732600-2743699]	0	1	1	1
NC_002655:32|Escherichia coli O157-H7 EDL933 chromosome I [2842700-2858849]	0	1	0	1
NC_002655:33|Escherichia coli O157-H7 EDL933 chromosome I [2927800-2938199]	0	1	0	1
NC_002655:34|Escherichia coli O157-H7 EDL933 chromosome I [3262500-3271549]	0	1	0	1
NC_002655:35|Escherichia coli O157-H7 EDL933 chromosome I [3277100-3292249]	0	1	0	1
NC_002655:36|Escherichia coli O157-H7 EDL933 chromosome I [3542900-3549799]	0	0	1	1

NC_002655:37|Escherichia coli O157-H7 EDL933 chromosome I [3553850-3567449]	1	1	1	1
NC_002655:38|Escherichia coli O157-H7 EDL933 chromosome I [3568350-3577949]	0	1	1	1
NC_002655:39|Escherichia coli O157-H7 EDL933 chromosome I [3772500-3804399]	0	1	0	1
NC_002655:40|Escherichia coli O157-H7 EDL933 chromosome I [3923300-3935649]	0	1	0	1
NC_002655:41|Escherichia coli O157-H7 EDL933 chromosome I [4070650-4076199]	0	0	0	0
NC_002655:42|Escherichia coli O157-H7 EDL933 chromosome I [4430500-4436499]	0	0	0	0
NC_002655:43|Escherichia coli O157-H7 EDL933 chromosome I [4574050-4580049]	0	0	0	0
NC_002655:44|Escherichia coli O157-H7 EDL933 chromosome I [4610000-4616799]	0	1	0	1
NC_002655:45|Escherichia coli O157-H7 EDL933 chromosome I [4656800-4694799]	0	1	0	1
NC_002655:46|Escherichia coli O157-H7 EDL933 chromosome I [4752050-4761649]	0	1	0	1
NC_002655:47|Escherichia coli O157-H7 EDL933 chromosome I [4863600-4867999]	0	0	0	0
NC_002655:48|Escherichia coli O157-H7 EDL933 chromosome I [5359050-5365199]	0	1	0	1
NC_002655:49|Escherichia coli O157-H7 EDL933 chromosome I [5386100-5398899]	0	1	0	1
NC_002655:50|Escherichia coli O157-H7 EDL933 chromosome I [5419600-5428949]	0	1	0	1
NC_002655:6|Escherichia coli O157-H7 EDL933 chromosome I [366800-371349]	0	1	0	1
NC_002655:7|Escherichia coli O157-H7 EDL933 chromosome I [811000-816549]	0	0	0	0
NC_002655:8|Escherichia coli O157-H7 EDL933 chromosome I [897450-903449]	0	0	0	0
NC_002663:2|Pasteurella multocida PM70 chromosome I [688100-692749]	0	0	0	0
NC_002663:4|Pasteurella multocida PM70 chromosome I [911850-923349]	0	0	0	0
NC_002663:5|Pasteurella multocida PM70 chromosome I [1180550-1188149]	0	0	0	0
NC_002663:6|Pasteurella multocida PM70 chromosome I [1337400-1343399]	0	0	0	0
NC_002663:7|Pasteurella multocida PM70 chromosome I [2180050-2185249]	0	0	0	0
NC_002695:11|Escherichia coli O157-H7-Sakai chromosome I [1331800-1341599]	0	0	0	0
NC_002695:12|Escherichia coli O157-H7-Sakai chromosome I [1343100-1350099]	0	1	0	1
NC_002695:13|Escherichia coli O157-H7-Sakai chromosome I [1367900-1378649]	0	1	1	1
NC_002695:14|Escherichia coli O157-H7-Sakai chromosome I [1383600-1389749]	1	1	0	1
NC_002695:15|Escherichia coli O157-H7-Sakai chromosome I [1416100-1429349]	1	1	0	1
NC_002695:16|Escherichia coli O157-H7-Sakai chromosome I [1575250-1588199]	0	1	0	1
NC_002695:17|Escherichia coli O157-H7-Sakai chromosome I [1617350-1625699]	0	1	1	1
NC_002695:2|Escherichia coli O157-H7-Sakai chromosome I [237450-243249]	0	1	0	1
NC_002695:22|Escherichia coli O157-H7-Sakai chromosome I [1931900-1938649]	0	1	0	1
NC_002695:25|Escherichia coli O157-H7-Sakai chromosome I [2096250-2113649]	0	1	0	1
NC_002695:27|Escherichia coli O157-H7-Sakai chromosome I [2657350-2668499]	1	1	0	1
NC_002695:28|Escherichia coli O157-H7-Sakai chromosome I [2772500-2788649]	0	1	0	1
NC_002695:29|Escherichia coli O157-H7-Sakai chromosome I [2857650-2867949]	0	1	0	1
NC_002695:30|Escherichia coli O157-H7-Sakai chromosome I [3192550-3202049]	0	1	0	1
NC_002695:31|Escherichia coli O157-H7-Sakai chromosome I [3207250-3222749]	0	1	0	1
NC_002695:32|Escherichia coli O157-H7-Sakai chromosome I [3473100-3479549]	0	0	1	1
NC_002695:33|Escherichia coli O157-H7-Sakai chromosome I [3486050-3500149]	1	1	1	1
NC_002695:34|Escherichia coli O157-H7-Sakai chromosome I [3501550-3509949]	0	1	0	1
NC_002695:35|Escherichia coli O157-H7-Sakai chromosome I [3705200-3736649]	0	1	0	1
NC_002695:36|Escherichia coli O157-H7-Sakai chromosome I [3856350-3868349]	0	1	0	1
NC_002695:37|Escherichia coli O157-H7-Sakai chromosome I [4003050-4008999]	0	0	0	0
NC_002695:38|Escherichia coli O157-H7-Sakai chromosome I [4363200-4369549]	0	0	0	0
NC_002695:39|Escherichia coli O157-H7-Sakai chromosome I [4384000-4389799]	0	0	0	0
NC_002695:40|Escherichia coli O157-H7-Sakai chromosome I [4541000-4548049]	0	1	0	1
NC_002695:41|Escherichia coli O157-H7-Sakai chromosome I [4587750-4625299]	0	1	0	1
NC_002695:42|Escherichia coli O157-H7-Sakai chromosome I [4683150-4693099]	0	1	0	1
NC_002695:44|Escherichia coli O157-H7-Sakai chromosome I [5329050-5335199]	0	1	0	1
NC_002695:45|Escherichia coli O157-H7-Sakai chromosome I [5356600-5368899]	0	1	0	1
NC_002695:46|Escherichia coli O157-H7-Sakai chromosome I [5389600-5398949]	0	1	0	1
NC_002695:8|Escherichia coli O157-H7-Sakai chromosome I [896100-901799]	0	0	0	0
NC_002696:1|Caulobacter crescentus CB15 chromosome I [2990900-2996899]	0	0	0	0
NC_002737:5|Streptococcus pyogenes M1 GAS chromosome I [557200-565049]	0	0	0	0
NC_002755:5|Mycobacterium tuberculosis CDC1551 chromosome I [1687550-1698499]	0	1	0	1
NC_002755:7|Mycobacterium tuberculosis CDC1551 chromosome I [2606750-2611299]	0	0	0	0
NC_002755:9|Mycobacterium tuberculosis CDC1551 chromosome I [3115400-3124799]	0	0	0	0
NC_002932:10|Chlorobium tepidum TLS chromosome I [1271450-1279799]	0	0	0	0
NC_002932:14|Chlorobium tepidum TLS chromosome I [1959300-1966999]	0	0	0	0
NC_002932:3|Chlorobium tepidum TLS chromosome I [225250-233899]	0	0	0	0
NC_002932:4|Chlorobium tepidum TLS chromosome I [299400-307899]	0	0	0	0
NC_002932:6|Chlorobium tepidum TLS chromosome I [634650-646649]	0	0	0	0
NC_002932:8|Chlorobium tepidum TLS chromosome I [1046050-1051999]	0	1	0	1
NC_002936:1|Dehalococcoides ethenogenes 195 chromosome I [73250-85049]	0	1	1	1
NC_002936:2|Dehalococcoides ethenogenes 195 chromosome I [291150-306449]	1	0	0	1
NC_002936:3|Dehalococcoides ethenogenes 195 chromosome I [801050-811299]	0	1	0	1
NC_002936:4|Dehalococcoides ethenogenes 195 chromosome I [1322250-1338849]	0	1	1	1
NC_002936:5|Dehalococcoides ethenogenes 195 chromosome I [1397000-1409899]	0	1	0	1
NC_002946:11|Neisseria gonorrhoeae FA1090 chromosome I [1024400-1033399]	0	1	0	1
NC_002946:12|Neisseria gonorrhoeae FA1090 chromosome I [1082300-1087149]	0	0	0	0
NC_002946:14|Neisseria gonorrhoeae FA1090 chromosome I [1346350-1354499]	0	0	0	0
NC_002946:15|Neisseria gonorrhoeae FA1090 chromosome I [1558650-1569799]	0	0	0	0
NC_002946:20|Neisseria gonorrhoeae FA1090 chromosome I [1938650-1950349]	0	0	0	0
NC_002946:3|Neisseria gonorrhoeae FA1090 chromosome I [225200-231149]	0	0	0	0
NC_002946:5|Neisseria gonorrhoeae FA1090 chromosome I [352600-357949]	0	0	0	0
NC_002947:25|Pseudomonas putida KT2440 chromosome I [5037650-5052449]	0	1	1	1
NC_002950:1|Porphyromonas gingivalis W83 chromosome I [126150-142399]	0	1	0	1
NC_002950:11|Porphyromonas gingivalis W83 chromosome I [1578800-1583399]	0	1	0	1
NC_002950:12|Porphyromonas gingivalis W83 chromosome I [1584650-1595699]	0	1	0	1
NC_002950:13|Porphyromonas gingivalis W83 chromosome I [1603150-1612799]	0	0	1	1
NC_002950:5|Porphyromonas gingivalis W83 chromosome I [883100-902249]	0	1	0	1
NC_002950:8|Porphyromonas gingivalis W83 chromosome I [1275300-1283299]	0	1	1	1
NC_002950:9|Porphyromonas gingivalis W83 chromosome I [1516250-1529899]	0	1	1	1
NC_002971:1|Coxiella burnetii RSA493 chromosome I [149000-155849]	0	1	0	1
NC_002971:3|Coxiella burnetii RSA493 chromosome I [624250-638399]	0	1	0	1
NC_002971:4|Coxiella burnetii RSA493 chromosome I [780550-790099]	0	0	0	0
NC_002971:5|Coxiella burnetii RSA493 chromosome I [863700-868149]	0	0	0	0
NC_002971:6|Coxiella burnetii RSA493 chromosome I [958000-965899]	0	0	0	0
NC_002971:7|Coxiella burnetii RSA493 chromosome I [1550850-1557499]	0	1	0	1

NC_002973:1|Listeria monocytogenes 4bF2365 chromosome I [473150-485649]	0	1	0	1
NC_002973:2|Listeria monocytogenes 4bF2365 chromosome I [1126200-1135999]	0	0	0	0
NC_002973:3|Listeria monocytogenes 4bF2365 chromosome I [2748500-2753349]	0	0	0	0
NC_002978:1|Wolbachia endosymbiont-of-Drosophila-melanogaster chromosome I [625550-631549]	0	0	0	0
NC_003062:4|Agrobacterium tumefaciens C58 chromosome circular [2139250-2141649]	0	0	0	0
NC_003062:5|Agrobacterium tumefaciens C58 chromosome circular [2309750-2317799]	0	0	0	0
NC_003062:6|Agrobacterium tumefaciens C58 chromosome circular [2491350-2498149]	0	0	0	0
NC_003063:1|Agrobacterium tumefaciens C58 chromosome linear [308600-324999]	0	0	0	0
NC_003063:4|Agrobacterium tumefaciens C58 chromosome linear [1132200-1145899]	0	0	0	0
NC_003063:5|Agrobacterium tumefaciens C58 chromosome linear [1362700-1375399]	0	0	0	0
NC_003064:1|Agrobacterium tumefaciens C58 plasmid AT [26900-44199]	0	0	0	0
NC_003065:1|Agrobacterium tumefaciens C58 plasmid Ti [32350-56499]	0	0	0	0
NC_003106:1|Sulfolobus tokodaii 7 chromosome I [56000-65249]	0	0	0	0
NC_003112:1|Neisseria meningitidis MC58 chromosome I [71050-81649]	0	0	0	0
NC_003112:10|Neisseria meningitidis MC58 chromosome I [879250-884499]	0	0	0	0
NC_003112:11|Neisseria meningitidis MC58 chromosome I [1428650-1446399]	0	1	1	1
NC_003112:12|Neisseria meningitidis MC58 chromosome I [1598000-1606899]	0	0	0	0
NC_003112:14|Neisseria meningitidis MC58 chromosome I [1826050-1860549]	0	1	1	1
NC_003112:15|Neisseria meningitidis MC58 chromosome I [1866500-1872299]	0	0	0	0
NC_003112:17|Neisseria meningitidis MC58 chromosome I [2219900-2234349]	0	1	0	1
NC_003112:2|Neisseria meningitidis MC58 chromosome I [101100-112299]	0	0	0	0
NC_003112:4|Neisseria meningitidis MC58 chromosome I [368550-376049]	0	1	0	1
NC_003112:6|Neisseria meningitidis MC58 chromosome I [523900-543249]	0	0	0	0
NC_003112:8|Neisseria meningitidis MC58 chromosome I [674500-686699]	0	1	0	1
NC_003116:10|Neisseria meningitidis Z2491 [759000-773449]	0	0	1	1
NC_003116:11|Neisseria meningitidis Z2491 [1022750-1033099]	0	0	0	0
NC_003116:12|Neisseria meningitidis Z2491 [1393350-1399099]	0	0	0	0
NC_003116:13|Neisseria meningitidis Z2491 [1522850-1531299]	0	1	1	1
NC_003116:14|Neisseria meningitidis Z2491 [1735000-1742799]	0	0	0	0
NC_003116:15|Neisseria meningitidis Z2491 [2066000-2074299]	0	1	0	1
NC_003116:4|Neisseria meningitidis Z2491 [177600-186949]	0	0	0	0
NC_003116:5|Neisseria meningitidis Z2491 [294900-306949]	0	0	0	0
NC_003116:8|Neisseria meningitidis Z2491 [665050-674449]	0	0	0	0
NC_003116:9|Neisseria meningitidis Z2491 [673050-690249]	0	0	0	0
NC_003197:1|Salmonella typhimurium LT2 chromosome I [31050-41749]	0	0	0	0
NC_003197:14|Salmonella typhimurium LT2 chromosome I [1172400-1180599]	0	0	0	0
NC_003197:15|Salmonella typhimurium LT2 chromosome I [1213250-1222899]	0	1	0	1
NC_003197:16|Salmonella typhimurium LT2 chromosome I [1322950-1337449]	0	1	0	1
NC_003197:17|Salmonella typhimurium LT2 chromosome I [1405150-1413549]	0	0	0	0
NC_003197:18|Salmonella typhimurium LT2 chromosome I [1473850-1502249]	0	1	0	1
NC_003197:19|Salmonella typhimurium LT2 chromosome I [1620050-1633149]	0	1	0	1
NC_003197:2|Salmonella typhimurium LT2 chromosome I [335300-345449]	0	1	0	1
NC_003197:21|Salmonella typhimurium LT2 chromosome I [1716450-1729599]	0	1	0	1
NC_003197:22|Salmonella typhimurium LT2 chromosome I [1758650-1767949]	0	0	0	0
NC_003197:23|Salmonella typhimurium LT2 chromosome I [1948950-1965349]	0	1	0	1
NC_003197:24|Salmonella typhimurium LT2 chromosome I [2157200-2178449]	0	1	0	1
NC_003197:26|Salmonella typhimurium LT2 chromosome I [2329400-2338499]	0	1	0	1
NC_003197:3|Salmonella typhimurium LT2 chromosome I [343250-350199]	0	1	0	1
NC_003197:31|Salmonella typhimurium LT2 chromosome I [2898200-2913849]	1	1	1	1
NC_003197:32|Salmonella typhimurium LT2 chromosome I [2922500-2930999]	0	0	0	0
NC_003197:33|Salmonella typhimurium LT2 chromosome I [3008450-3028949]	0	1	0	1
NC_003197:34|Salmonella typhimurium LT2 chromosome I [3039450-3048649]	0	1	0	1
NC_003197:36|Salmonella typhimurium LT2 chromosome I [3906800-3917849]	0	1	0	1
NC_003197:4|Salmonella typhimurium LT2 chromosome I [380750-391049]	0	1	0	1
NC_003197:40|Salmonella typhimurium LT2 chromosome I [4474200-4484899]	0	1	0	1
NC_003197:41|Salmonella typhimurium LT2 chromosome I [4493900-4502649]	0	0	0	0
NC_003197:42|Salmonella typhimurium LT2 chromosome I [4555550-4564249]	0	1	1	1
NC_003197:43|Salmonella typhimurium LT2 chromosome I [4752400-4760799]	0	0	0	0
NC_003197:6|Salmonella typhimurium LT2 chromosome I [608700-618099]	0	1	0	1
NC_003197:7|Salmonella typhimurium LT2 chromosome I [780000-793999]	0	1	1	1
NC_003197:9|Salmonella typhimurium LT2 chromosome I [923300-933349]	0	1	0	1
NC_003198:1|Salmonella enterica subsp. enterica serovar Typhi CT18 chromosome I [31350-40949]	0	0	0	0
NC_003198:11|Salmonella enterica subsp. enterica serovar Typhi CT18 chromosome I [1081850-1090849]	0	0	0	0
NC_003198:12|Salmonella enterica subsp. enterica serovar Typhi CT18 chromosome I [1123500-1133049]	0	1	0	1
NC_003198:14|Salmonella enterica subsp. enterica serovar Typhi CT18 chromosome I [1342600-1353299]	0	0	0	0
NC_003198:16|Salmonella enterica subsp. enterica serovar Typhi CT18 chromosome I [1382900-1390699]	0	0	0	0
NC_003198:17|Salmonella enterica subsp. enterica serovar Typhi CT18 chromosome I [1452500-1457349]	0	0	0	0
NC_003198:18|Salmonella enterica subsp. enterica serovar Typhi CT18 chromosome I [1465150-1478449]	0	1	0	1
NC_003198:19|Salmonella enterica subsp. enterica serovar Typhi CT18 chromosome I [1622850-1650099]	0	1	0	1
NC_003198:21|Salmonella enterica subsp. enterica serovar Typhi CT18 chromosome I [1774100-1791899]	0	1	1	1
NC_003198:22|Salmonella enterica subsp. enterica serovar Typhi CT18 chromosome I [1871000-1891049]	0	1	0	1
NC_003198:23|Salmonella enterica subsp. enterica serovar Typhi CT18 chromosome I [1913250-1926849]	0	1	1	1
NC_003198:24|Salmonella enterica subsp. enterica serovar Typhi CT18 chromosome I [2113950-2136899]	0	1	0	1
NC_003198:25|Salmonella enterica subsp. enterica serovar Typhi CT18 chromosome I [2185800-2197999]	0	1	0	1
NC_003198:27|Salmonella enterica subsp. enterica serovar Typhi CT18 chromosome I [2458300-2466499]	0	0	0	0
NC_003198:28|Salmonella enterica subsp. enterica serovar Typhi CT18 chromosome I [2775550-2784049]	0	0	0	0
NC_003198:29|Salmonella enterica subsp. enterica serovar Typhi CT18 chromosome I [2861200-2880949]	0	1	0	1
NC_003198:3|Salmonella enterica subsp. enterica serovar Typhi CT18 chromosome I [345250-351699]	0	1	0	1
NC_003198:30|Salmonella enterica subsp. enterica serovar Typhi CT18 chromosome I [2891700-2899099]	0	0	0	0
NC_003198:33|Salmonella enterica subsp. enterica serovar Typhi CT18 chromosome I [3130450-3140999]	0	1	0	1
NC_003198:37|Salmonella enterica subsp. enterica serovar Typhi CT18 chromosome I [3928650-3940349]	0	1	0	1
NC_003198:39|Salmonella enterica subsp. enterica serovar Typhi CT18 chromosome I [4320250-4330349]	0	1	0	1
NC_003198:4|Salmonella enterica subsp. enterica serovar Typhi CT18 chromosome I [350500-360399]	0	1	0	1
NC_003198:40|Salmonella enterica subsp. enterica serovar Typhi CT18 chromosome I [4338700-4347799]	0	0	0	0
NC_003198:41|Salmonella enterica subsp. enterica serovar Typhi CT18 chromosome I [4400500-4409099]	0	1	1	1
NC_003198:43|Salmonella enterica subsp. enterica serovar Typhi CT18 chromosome I [4453100-4464249]	1	1	0	1
NC_003198:44|Salmonella enterica subsp. enterica serovar Typhi CT18 chromosome I [4494850-4499699]	0	0	0	0
NC_003198:45|Salmonella enterica subsp. enterica serovar Typhi CT18 chromosome I [4518750-4534799]	1	1	1	1
NC_003198:47|Salmonella enterica subsp. enterica serovar Typhi CT18 chromosome I [4693000-4703449]	0	1	0	1

NC_003198:7|Salmonella enterica subsp. enterica serovar Typhi CT18 chromosome I [601350-610049]	0	1	0	1
NC_003198:8|Salmonella enterica subsp. enterica serovar Typhi CT18 chromosome I [756350-769199]	0	1	1	1
NC_003210:1|Listeria monocytogenes EGD-e chromosome I [331400-336799]	0	0	0	0
NC_003210:2|Listeria monocytogenes EGD-e chromosome I [1151350-1159249]	0	0	0	0
NC_003212:1|Listeria innocua Clip11262 chromosome I [498500-504299]	0	0	0	0
NC_003228:1|Bacteroides fragilis NCTC 9343 chromosome I [897550-909099]	0	1	0	1
NC_003228:2|Bacteroides fragilis NCTC 9343 chromosome I [1798900-1803149]	0	0	0	0
NC_003228:3|Bacteroides fragilis NCTC 9343 chromosome I [3260900-3266249]	0	1	0	1
NC_003228:4|Bacteroides fragilis NCTC 9343 chromosome I [3380900-3386699]	0	1	0	1
NC_003228:5|Bacteroides fragilis NCTC 9343 chromosome I [4081150-4087449]	0	1	0	1
NC_003304:4|Agrobacterium tumefaciens C58 chromosome circular [2139350-2144949]	0	0	0	0
NC_003304:5|Agrobacterium tumefaciens C58 chromosome circular [2309850-2315299]	0	0	0	0
NC_003304:6|Agrobacterium tumefaciens C58 chromosome circular [2491450-2498249]	0	0	0	0
NC_003305:2|Agrobacterium tumefaciens C58 linear chromosome [696950-711449]	0	0	0	0
NC_003305:3|Agrobacterium tumefaciens C58 linear chromosome [926250-939199]	0	0	0	0
NC_003305:6|Agrobacterium tumefaciens C58 linear chromosome [1747150-1762599]	0	0	0	0
NC_003306:1|Agrobacterium tumefaciens str. C58 (U. Washington) plasmid AT [26600-43899]	0	0	0	0
NC_003308:1|Agrobacterium tumefaciens str. C58 (U. Washington) plasmid Ti [850-22999]	0	0	0	0
NC_003317:4|Brucella melitensis 16M chromosome I [932650-941099]	0	1	1	1
NC_003317:5|Brucella melitensis 16M chromosome I [1032550-1049499]	0	1	1	1
NC_003317:6|Brucella melitensis 16M chromosome I [1204750-1210549]	0	0	1	1
NC_003317:7|Brucella melitensis 16M chromosome I [1266100-1281349]	0	1	0	1
NC_003317:8|Brucella melitensis 16M chromosome I [1447200-1468399]	0	1	1	1
NC_003317:9|Brucella melitensis 16M chromosome I [1707800-1715849]	0	1	0	1
NC_003318:10|Brucella melitensis 16M chromosome II [1134250-1139299]	0	0	0	0
NC_003318:2|Brucella melitensis 16M chromosome II [254800-259049]	0	0	0	0
NC_003318:4|Brucella melitensis 16M chromosome II [678000-686149]	0	0	0	0
NC_003318:5|Brucella melitensis 16M chromosome II [745050-753299]	0	0	1	1
NC_003318:6|Brucella melitensis 16M chromosome II [839900-844249]	0	0	0	0
NC_003318:7|Brucella melitensis 16M chromosome II [941600-951299]	0	0	0	0
NC_003552:1|Methanosarcina acetivorans C2A chromosome I [1250500-1258999]	0	1	1	1
NC_003552:10|Methanosarcina acetivorans C2A chromosome I [3225900-3234349]	0	0	0	0
NC_003552:11|Methanosarcina acetivorans C2A chromosome I [3358550-3362299]	0	0	0	0
NC_003552:12|Methanosarcina acetivorans C2A chromosome I [4118800-4122899]	0	0	0	0
NC_003552:13|Methanosarcina acetivorans C2A chromosome I [4323250-4326599]	0	1	0	1
NC_003552:14|Methanosarcina acetivorans C2A chromosome I [4641650-4658449]	0	1	0	1
NC_003552:15|Methanosarcina acetivorans C2A chromosome I [4719850-4724699]	0	0	0	0
NC_003552:2|Methanosarcina acetivorans C2A chromosome I [1403150-1409949]	0	1	0	1
NC_003552:3|Methanosarcina acetivorans C2A chromosome I [1831400-1835049]	0	0	0	0
NC_003552:4|Methanosarcina acetivorans C2A chromosome I [1844650-1854299]	0	1	0	1
NC_003552:5|Methanosarcina acetivorans C2A chromosome I [2318750-2336149]	0	1	0	1
NC_003552:6|Methanosarcina acetivorans C2A chromosome I [2510050-2517099]	0	1	0	1
NC_003552:7|Methanosarcina acetivorans C2A chromosome I [2639650-2645849]	0	0	0	0
NC_003552:8|Methanosarcina acetivorans C2A chromosome I [2682650-2693299]	0	0	1	1
NC_003552:9|Methanosarcina acetivorans C2A chromosome I [2955950-2960199]	0	0	0	0
NC_003869:5|Thermoanaerobacter tengcongensis MB4T chromosome I [1652350-1660099]	0	0	0	0
NC_003901:1|Methanosarcina mazei Go1 chromosome I [37900-40499]	0	0	0	0
NC_003901:10|Methanosarcina mazei Go1 chromosome I [3208250-3218049]	0	1	0	1
NC_003901:11|Methanosarcina mazei Go1 chromosome I [3297600-3305899]	0	0	0	0
NC_003901:12|Methanosarcina mazei Go1 chromosome I [3553750-3557999]	0	0	0	0
NC_003901:13|Methanosarcina mazei Go1 chromosome I [3671850-3677399]	0	0	1	1
NC_003901:14|Methanosarcina mazei Go1 chromosome I [3838350-3842549]	0	0	0	0
NC_003901:15|Methanosarcina mazei Go1 chromosome I [3846250-3849799]	0	0	0	0
NC_003901:2|Methanosarcina mazei Go1 chromosome I [780150-785249]	0	1	0	1
NC_003901:3|Methanosarcina mazei Go1 chromosome I [1031500-1036299]	0	1	0	1
NC_003901:5|Methanosarcina mazei Go1 chromosome I [1389100-1396999]	0	1	0	1
NC_003901:6|Methanosarcina mazei Go1 chromosome I [1951450-1962049]	0	1	0	1
NC_003901:7|Methanosarcina mazei Go1 chromosome I [2416000-2421349]	0	0	0	0
NC_003901:8|Methanosarcina mazei Go1 chromosome I [2504100-2519299]	0	1	0	1
NC_003901:9|Methanosarcina mazei Go1 chromosome I [2627500-2636749]	0	0	0	0
NC_003910:1|Colwellia psychrerythraea 34H chromosome I [493800-502199]	0	0	0	0
NC_003910:2|Colwellia psychrerythraea 34H chromosome I [2183150-2189099]	0	0	0	0
NC_004129:11|Pseudomonas fluorescens Pf-5 chromosome I [6265000-6276999]	0	0	0	0
NC_004193:1|Oceanobacillus iheyensis HTE831 chromosome I [2972050-2978299]	0	0	0	0
NC_004310:1|Brucella suis 1330 chromosome I [264950-272949]	0	1	0	1
NC_004310:2|Brucella suis 1330 chromosome I [512200-535549]	0	1	1	1
NC_004310:3|Brucella suis 1330 chromosome I [710750-719599]	0	1	0	1
NC_004310:4|Brucella suis 1330 chromosome I [771500-778099]	0	1	0	1
NC_004310:5|Brucella suis 1330 chromosome I [935400-941749]	0	0	1	1
NC_004310:6|Brucella suis 1330 chromosome I [942500-952899]	0	1	0	1
NC_004310:7|Brucella suis 1330 chromosome I [1047250-1054349]	0	1	0	1
NC_004310:8|Brucella suis 1330 chromosome I [1543900-1546699]	0	0	0	0
NC_004311:1|Brucella suis 1330 chromosome II [128450-133499]	0	0	0	0
NC_004311:3|Brucella suis 1330 chromosome II [317000-324649]	0	0	0	0
NC_004311:4|Brucella suis 1330 chromosome II [441700-447049]	0	0	0	0
NC_004311:5|Brucella suis 1330 chromosome II [536000-542049]	0	0	1	1
NC_004311:6|Brucella suis 1330 chromosome II [602850-608699]	0	0	0	0
NC_004337:1|Shigella flexneri 2a-301 chromosome I [313750-330349]	0	1	1	1
NC_004337:10|Shigella flexneri 2a-301 chromosome I [1756200-1765499]	0	0	0	0
NC_004337:12|Shigella flexneri 2a-301 chromosome I [2004750-2012199]	0	0	1	1
NC_004337:13|Shigella flexneri 2a-301 chromosome I [2035400-2048049]	0	1	0	1
NC_004337:15|Shigella flexneri 2a-301 chromosome I [2102850-2118699]	0	1	0	1
NC_004337:19|Shigella flexneri 2a-301 chromosome I [2481450-2493299]	0	1	0	1
NC_004337:2|Shigella flexneri 2a-301 chromosome I [371950-379449]	0	1	0	1
NC_004337:24|Shigella flexneri 2a-301 chromosome I [3076600-3085249]	0	1	0	1
NC_004337:26|Shigella flexneri 2a-301 chromosome I [3254600-3260999]	0	0	0	0
NC_004337:27|Shigella flexneri 2a-301 chromosome I [3593000-3604749]	0	1	1	1
NC_004337:28|Shigella flexneri 2a-301 chromosome I [3624500-3635699]	0	0	0	0
NC_004337:29|Shigella flexneri 2a-301 chromosome I [3764500-3774499]	0	1	0	1

NC_004337:33|Shigella flexneri 2a-301 chromosome I [3908750-3916649]	0	0	0	0
NC_004337:34|Shigella flexneri 2a-301 chromosome I [4377300-4388949]	0	1	0	1
NC_004337:6|Shigella flexneri 2a-301 chromosome I [1073650-1082449]	0	0	0	0
NC_004337:7|Shigella flexneri 2a-301 chromosome I [1195850-1202899]	0	0	0	0
NC_004347:1|Shewanella oneidensis MR-1 chromosome I [723250-729149]	0	1	0	1
NC_004347:12|Shewanella oneidensis MR-1 chromosome I [4449500-4469549]	0	1	0	1
NC_004347:2|Shewanella oneidensis MR-1 chromosome I [794700-801149]	0	0	0	0
NC_004347:6|Shewanella oneidensis MR-1 chromosome I [3200950-3208899]	0	1	0	1
NC_004347:7|Shewanella oneidensis MR-1 chromosome I [3310750-3326849]	0	1	0	1
NC_004431:10|Escherichia coli CFT073 chromosome I [410150-414249]	0	0	0	0
NC_004431:13|Escherichia coli CFT073 chromosome I [1122900-1134849]	0	0	0	0
NC_004431:14|Escherichia coli CFT073 chromosome I [1170500-1196499]	1	1	0	1
NC_004431:15|Escherichia coli CFT073 chromosome I [1213500-1223799]	0	1	0	1
NC_004431:16|Escherichia coli CFT073 chromosome I [1407250-1412099]	0	1	0	1
NC_004431:17|Escherichia coli CFT073 chromosome I [1416000-1423049]	0	0	0	0
NC_004431:18|Escherichia coli CFT073 chromosome I [1447900-1463299]	0	1	0	1
NC_004431:19|Escherichia coli CFT073 chromosome I [1648700-1654249]	0	0	0	0
NC_004431:22|Escherichia coli CFT073 chromosome I [1795500-1803199]	0	1	0	1
NC_004431:23|Escherichia coli CFT073 chromosome I [2158850-2163049]	0	0	0	0
NC_004431:24|Escherichia coli CFT073 chromosome I [2184450-2216749]	0	1	0	1
NC_004431:27|Escherichia coli CFT073 chromosome I [2318650-2333749]	0	1	0	1
NC_004431:28|Escherichia coli CFT073 chromosome I [2388300-2401549]	0	1	0	1
NC_004431:29|Escherichia coli CFT073 chromosome I [2451250-2459349]	0	1	0	1
NC_004431:30|Escherichia coli CFT073 chromosome I [2476700-2482999]	0	1	0	1
NC_004431:31|Escherichia coli CFT073 chromosome I [2752200-2761199]	0	0	1	1
NC_004431:32|Escherichia coli CFT073 chromosome I [2761150-2776849]	0	1	0	1
NC_004431:34|Escherichia coli CFT073 chromosome I [3045550-3052099]	0	0	0	0
NC_004431:36|Escherichia coli CFT073 chromosome I [3248900-3254899]	0	0	0	0
NC_004431:37|Escherichia coli CFT073 chromosome I [3411600-3425099]	0	1	0	1
NC_004431:4|Escherichia coli CFT073 chromosome I [282950-304499]	1	1	0	1
NC_004431:40|Escherichia coli CFT073 chromosome I [3469850-3478249]	1	0	0	1
NC_004431:41|Escherichia coli CFT073 chromosome I [3516200-3529949]	0	1	0	1
NC_004431:43|Escherichia coli CFT073 chromosome I [3989800-3999949]	0	1	0	1
NC_004431:44|Escherichia coli CFT073 chromosome I [4227950-4234799]	0	1	0	1
NC_004431:45|Escherichia coli CFT073 chromosome I [4271800-4282749]	0	1	0	1
NC_004431:46|Escherichia coli CFT073 chromosome I [4294100-4304299]	0	0	0	0
NC_004431:47|Escherichia coli CFT073 chromosome I [4651600-4657299]	0	1	0	1
NC_004431:48|Escherichia coli CFT073 chromosome I [4932650-4936599]	0	0	1	1
NC_004431:49|Escherichia coli CFT073 chromosome I [4958750-4968249]	0	0	1	1
NC_004431:50|Escherichia coli CFT073 chromosome I [5128800-5138249]	0	0	0	0
NC_004431:8|Escherichia coli CFT073 chromosome I [369350-377999]	0	0	1	1
NC_004431:9|Escherichia coli CFT073 chromosome I [384200-391149]	0	1	0	1
NC_004547:15|Erwinia carotovora SCRI1043 chromosome I [1605200-1635399]	0	1	0	1
NC_004547:18|Erwinia carotovora SCRI1043 chromosome I [1878000-1936849]	0	1	1	1
NC_004547:19|Erwinia carotovora SCRI1043 chromosome I [1973400-1986899]	0	1	0	1
NC_004547:2|Erwinia carotovora SCRI1043 chromosome I [168700-182149]	0	1	0	1
NC_004547:20|Erwinia carotovora SCRI1043 chromosome I [2011700-2018499]	0	0	0	0
NC_004547:29|Erwinia carotovora SCRI1043 chromosome I [3212650-3263049]	0	1	1	1
NC_004547:7|Erwinia carotovora SCRI1043 chromosome I [641800-652449]	0	1	0	1
NC_004567:1|Lactobacillus plantarum WCFS1 chromosome I [348400-355099]	0	0	0	0
NC_004567:10|Lactobacillus plantarum WCFS1 chromosome I [1802150-1812349]	0	0	1	1
NC_004567:12|Lactobacillus plantarum WCFS1 chromosome I [2797150-2802449]	0	0	0	0
NC_004567:13|Lactobacillus plantarum WCFS1 chromosome I [2806350-2810599]	0	0	0	0
NC_004567:14|Lactobacillus plantarum WCFS1 chromosome I [3127950-3135149]	0	0	0	0
NC_004567:15|Lactobacillus plantarum WCFS1 chromosome I [3158000-3163499]	0	0	0	0
NC_004567:16|Lactobacillus plantarum WCFS1 chromosome I [3191350-3201299]	0	0	0	0
NC_004567:2|Lactobacillus plantarum WCFS1 chromosome I [363700-375949]	0	0	0	0
NC_004567:3|Lactobacillus plantarum WCFS1 chromosome I [430700-440899]	0	0	0	0
NC_004567:7|Lactobacillus plantarum WCFS1 chromosome I [1071550-1084449]	0	0	0	0
NC_004567:9|Lactobacillus plantarum WCFS1 chromosome I [1538650-1545249]	0	0	0	0
NC_004578:2|Pseudomonas syringae DC3000 chromosome I [24550-44999]	1	1	1	1
NC_004578:51|Pseudomonas syringae DC3000 chromosome I [5375950-5382149]	0	1	0	1
NC_004578:52|Pseudomonas syringae DC3000 chromosome I [5388650-5404999]	0	0	1	1
NC_004578:61|Pseudomonas syringae DC3000 chromosome I [6339850-6352599]	0	1	0	1
NC_004606:6|Streptococcus pyogenes SSI-1 chromosome I [476450-479749]	0	0	0	0
NC_004606:8|Streptococcus pyogenes SSI-1 chromosome I [651400-658999]	0	0	0	0
NC_004606:9|Streptococcus pyogenes SSI-1 chromosome I [907950-913949]	0	0	0	0
NC_004631:10|Salmonella enterica Ty2 chromosome I [1328750-1356649]	0	1	0	1
NC_004631:11|Salmonella enterica Ty2 chromosome I [1506050-1514549]	0	1	0	1
NC_004631:12|Salmonella enterica Ty2 chromosome I [1522550-1528149]	0	0	0	0
NC_004631:13|Salmonella enterica Ty2 chromosome I [1588250-1596249]	0	0	0	0
NC_004631:15|Salmonella enterica Ty2 chromosome I [1629000-1637799]	0	0	0	0
NC_004631:17|Salmonella enterica Ty2 chromosome I [1845000-1853899]	0	1	0	1
NC_004631:18|Salmonella enterica Ty2 chromosome I [1887650-1896699]	0	0	1	1
NC_004631:2|Salmonella enterica Ty2 chromosome I [535650-544949]	0	0	0	0
NC_004631:20|Salmonella enterica Ty2 chromosome I [2209850-2222349]	0	1	0	1
NC_004631:21|Salmonella enterica Ty2 chromosome I [2370400-2377849]	0	1	0	1
NC_004631:23|Salmonella enterica Ty2 chromosome I [2588500-2598149]	0	1	0	1
NC_004631:24|Salmonella enterica Ty2 chromosome I [2618900-2627249]	0	1	0	1
NC_004631:25|Salmonella enterica Ty2 chromosome I [2625700-2633699]	0	1	0	1
NC_004631:26|Salmonella enterica Ty2 chromosome I [2633550-2640299]	0	1	0	1
NC_004631:27|Salmonella enterica Ty2 chromosome I [2762350-2769799]	0	0	0	0
NC_004631:28|Salmonella enterica Ty2 chromosome I [2847050-2866799]	0	1	0	1
NC_004631:29|Salmonella enterica Ty2 chromosome I [2877500-2884999]	0	0	0	0
NC_004631:32|Salmonella enterica Ty2 chromosome I [3115950-3127799]	0	1	0	1
NC_004631:36|Salmonella enterica Ty2 chromosome I [3914150-3925849]	0	1	0	1
NC_004631:38|Salmonella enterica Ty2 chromosome I [4304900-4314999]	0	1	0	1
NC_004631:39|Salmonella enterica Ty2 chromosome I [4323700-4332449]	0	0	0	0
NC_004631:4|Salmonella enterica Ty2 chromosome I [803700-815149]	0	1	0	1

NC_004631:40|Salmonella enterica Ty2 chromosome I [4385150-4393599]	0	1	1	1
NC_004631:42|Salmonella enterica Ty2 chromosome I [4441900-4447849]	1	1	0	1
NC_004631:43|Salmonella enterica Ty2 chromosome I [4478900-4483699]	0	0	0	0
NC_004631:44|Salmonella enterica Ty2 chromosome I [4501350-4517799]	0	1	1	1
NC_004631:46|Salmonella enterica Ty2 chromosome I [4676100-4685499]	0	0	0	0
NC_004631:47|Salmonella enterica Ty2 chromosome I [4696650-4707749]	0	0	0	0
NC_004631:5|Salmonella enterica Ty2 chromosome I [865850-887849]	0	1	0	1
NC_004631:6|Salmonella enterica Ty2 chromosome I [952500-978749]	0	1	0	1
NC_004631:7|Salmonella enterica Ty2 chromosome I [1093300-1108849]	0	1	0	1
NC_004631:8|Salmonella enterica Ty2 chromosome I [1186900-1204199]	0	1	1	1
NC_004631:9|Salmonella enterica Ty2 chromosome I [1273600-1281999]	0	0	0	0
NC_004663:1|Bacteroides thetaiotaomicron VPI-5482 chromosome I [472550-480149]	0	0	0	0
NC_004663:2|Bacteroides thetaiotaomicron VPI-5482 chromosome I [813200-817599]	0	1	0	1
NC_004663:3|Bacteroides thetaiotaomicron VPI-5482 chromosome I [1142500-1160799]	0	1	1	1
NC_004663:4|Bacteroides thetaiotaomicron VPI-5482 chromosome I [1668300-1679999]	0	0	0	0
NC_004663:5|Bacteroides thetaiotaomicron VPI-5482 chromosome I [5641250-5646199]	0	0	0	0
NC_004668:1|Enterococcus faecalis V583 chromosome I [131150-134949]	0	0	0	0
NC_004668:2|Enterococcus faecalis V583 chromosome I [483950-490149]	0	1	0	1
NC_004668:3|Enterococcus faecalis V583 chromosome I [496650-502649]	0	0	0	0
NC_004668:4|Enterococcus faecalis V583 chromosome I [514350-521249]	0	0	0	0
NC_004668:5|Enterococcus faecalis V583 chromosome I [562500-566649]	0	1	0	1
NC_004668:6|Enterococcus faecalis V583 chromosome I [566500-571749]	0	0	0	0
NC_004722:1|Bacillus cereus ATCC14579 chromosome I [4987050-4991049]	0	0	0	0
NC_004741:1|Shigella flexneri 2a-2457T chromosome I [312750-329499]	0	1	1	1
NC_004741:10|Shigella flexneri 2a-2457T chromosome I [1794150-1802699]	0	0	0	0
NC_004741:12|Shigella flexneri 2a-2457T chromosome I [1981650-1988849]	0	0	0	0
NC_004741:13|Shigella flexneri 2a-2457T chromosome I [2010550-2023449]	0	1	1	1
NC_004741:15|Shigella flexneri 2a-2457T chromosome I [2085750-2100949]	0	1	0	1
NC_004741:16|Shigella flexneri 2a-2457T chromosome I [2172050-2179499]	0	0	0	0
NC_004741:17|Shigella flexneri 2a-2457T chromosome I [2448000-2454399]	0	0	0	0
NC_004741:18|Shigella flexneri 2a-2457T chromosome I [2458750-2476499]	0	1	0	1
NC_004741:2|Shigella flexneri 2a-2457T chromosome I [371100-378499]	0	1	0	1
NC_004741:23|Shigella flexneri 2a-2457T chromosome I [3069050-3077249]	0	0	0	0
NC_004741:25|Shigella flexneri 2a-2457T chromosome I [3245600-3252149]	0	0	0	0
NC_004741:26|Shigella flexneri 2a-2457T chromosome I [3854550-3862049]	0	0	0	0
NC_004741:30|Shigella flexneri 2a-2457T chromosome I [3960900-3968949]	0	0	0	0
NC_004741:31|Shigella flexneri 2a-2457T chromosome I [3998650-4007949]	0	1	0	1
NC_004741:32|Shigella flexneri 2a-2457T chromosome I [4135000-4145199]	0	1	1	1
NC_004741:33|Shigella flexneri 2a-2457T chromosome I [4160550-4177499]	0	1	0	1
NC_004741:34|Shigella flexneri 2a-2457T chromosome I [4368600-4380049]	0	1	0	1
NC_004741:6|Shigella flexneri 2a-2457T chromosome I [1079000-1085349]	0	0	0	0
NC_004741:7|Shigella flexneri 2a-2457T chromosome I [1198750-1205699]	0	0	0	0
NC_004757:11|Nitrosomonas europaea ATCC19718 chromosome I [2457200-2464849]	0	0	0	0
NC_004757:7|Nitrosomonas europaea ATCC19718 chromosome I [1094100-1105699]	0	0	0	0
NC_004829:1|Mycoplasma gallisepticum R chromosome I [365950-368999]	0	0	0	0
NC_005027:1|Rhodopirellula baltica SH-1 chromosome I [653600-657049]	0	0	0	0
NC_005027:13|Rhodopirellula baltica SH-1 chromosome I [2823750-2837449]	0	0	0	0
NC_005027:19|Rhodopirellula baltica SH-1 chromosome I [4649450-4653699]	0	0	0	0
NC_005027:20|Rhodopirellula baltica SH-1 chromosome I [4933700-4941649]	0	0	0	0
NC_005027:21|Rhodopirellula baltica SH-1 chromosome I [5330650-5338299]	0	0	1	1
NC_005027:24|Rhodopirellula baltica SH-1 chromosome I [5833500-5844799]	0	0	0	0
NC_005027:25|Rhodopirellula baltica SH-1 chromosome I [6083900-6087649]	0	0	0	0
NC_005027:3|Rhodopirellula baltica SH-1 chromosome I [1299400-1318849]	0	0	1	1
NC_005027:8|Rhodopirellula baltica SH-1 chromosome I [2104050-2116349]	0	1	1	1
NC_005027:9|Rhodopirellula baltica SH-1 chromosome I [2181500-2191499]	0	0	0	0
NC_005071:1|Prochlorococcus marinus MIT9313 chromosome I [91800-98599]	0	1	0	1
NC_005071:10|Prochlorococcus marinus MIT9313 chromosome I [528950-536549]	0	0	0	0
NC_005071:11|Prochlorococcus marinus MIT9313 chromosome I [540550-549549]	0	0	0	0
NC_005071:12|Prochlorococcus marinus MIT9313 chromosome I [678350-687399]	0	0	0	0
NC_005071:13|Prochlorococcus marinus MIT9313 chromosome I [709000-717749]	0	0	0	0
NC_005071:14|Prochlorococcus marinus MIT9313 chromosome I [790450-803049]	0	1	0	1
NC_005071:16|Prochlorococcus marinus MIT9313 chromosome I [870900-877649]	0	0	0	0
NC_005071:17|Prochlorococcus marinus MIT9313 chromosome I [917450-939349]	0	0	0	0
NC_005071:2|Prochlorococcus marinus MIT9313 chromosome I [97750-125249]	1	1	0	1
NC_005071:20|Prochlorococcus marinus MIT9313 chromosome I [1115950-1125499]	0	0	0	0
NC_005071:21|Prochlorococcus marinus MIT9313 chromosome I [1648450-1655749]	0	0	0	0
NC_005071:22|Prochlorococcus marinus MIT9313 chromosome I [2183550-2191999]	0	0	0	0
NC_005071:4|Prochlorococcus marinus MIT9313 chromosome I [177000-189199]	0	0	0	0
NC_005071:6|Prochlorococcus marinus MIT9313 chromosome I [296150-303449]	0	0	0	0
NC_005071:7|Prochlorococcus marinus MIT9313 chromosome I [314500-321549]	0	1	0	1
NC_005071:8|Prochlorococcus marinus MIT9313 chromosome I [328500-338599]	0	1	0	1
NC_005071:9|Prochlorococcus marinus MIT9313 chromosome I [340250-346399]	0	1	0	1
NC_005085:2|Chromobacterium violaceum ATCC12472 chromosome I [1302150-1313149]	0	1	0	1
NC_005085:5|Chromobacterium violaceum ATCC12472 chromosome I [4343850-4356999]	0	1	0	1
NC_005126:1|Photorhabdus luminescens TTO1 chromosome I [1071500-1080149]	0	0	0	0
NC_005126:2|Photorhabdus luminescens TTO1 chromosome I [2825800-2833049]	0	0	0	0
NC_005126:3|Photorhabdus luminescens TTO1 chromosome I [2909900-2914749]	0	0	0	0
NC_005126:4|Photorhabdus luminescens TTO1 chromosome I [4453750-4461949]	0	0	0	0
NC_005126:5|Photorhabdus luminescens TTO1 chromosome I [4710700-4717099]	0	0	0	0
NC_005126:6|Photorhabdus luminescens TTO1 chromosome I [5591200-5596749]	0	0	0	0
NC_005363:11|Bdellovibrio bacteriovorus HD100 chromosome I [2586650-2605799]	0	1	0	1
NC_005363:14|Bdellovibrio bacteriovorus HD100 chromosome I [3566400-3583049]	0	1	0	1
NC_005363:8|Bdellovibrio bacteriovorus HD100 chromosome I [1608100-1637849]	0	1	0	1
NC_005773:27|Pseudomonas syringae 1448A chromosome I [4290750-4314699]	1	1	1	1
NC_005773:30|Pseudomonas syringae 1448A chromosome I [4801350-4820399]	1	1	1	1
NC_005773:34|Pseudomonas syringae 1448A chromosome I [5170400-5197999]	1	1	1	1
NC_005877:1|Picrophilus torridus DSM9790 chromosome I [25300-35849]	0	0	0	0
NC_005877:2|Picrophilus torridus DSM9790 chromosome I [49150-54099]	0	0	0	0
NC_005877:3|Picrophilus torridus DSM9790 chromosome I [82900-94649]	0	0	0	0

NC_005877:4|Picrophilus torridus DSM9790 chromosome I [335700-348599]	0	0	0	0
NC_005955:1|Bartonella quintana Toulouse chromosome I [782650-785749]	0	0	0	0
NC_005956:1|Bartonella henselae Houston-1 chromosome I [1053450-1056699]	0	0	0	0
NC_005966:1|Acinetobacter sp ADP1 chromosome I [66550-70549]	0	0	0	0
NC_005966:4|Acinetobacter sp ADP1 chromosome I [2094850-2102299]	0	0	0	0
NC_005966:5|Acinetobacter sp ADP1 chromosome I [2114000-2123199]	0	1	0	1
NC_005966:6|Acinetobacter sp ADP1 chromosome I [2657750-2668249]	0	1	0	1
NC_005966:8|Acinetobacter sp ADP1 chromosome I [2723000-2731749]	0	0	1	1
NC_006085:3|Propionibacterium acnes KPA171202 chromosome I [861000-869649]	0	0	0	0
NC_006138:1|Desulfotalea psychrophila LSv54 chromosome I [34500-40799]	0	0	0	0
NC_006138:10|Desulfotalea psychrophila LSv54 chromosome I [2320550-2328949]	0	1	0	1
NC_006138:12|Desulfotalea psychrophila LSv54 chromosome I [2633900-2642799]	0	0	0	0
NC_006138:3|Desulfotalea psychrophila LSv54 chromosome I [438500-443899]	0	0	1	1
NC_006138:4|Desulfotalea psychrophila LSv54 chromosome I [704000-710199]	0	0	0	0
NC_006138:5|Desulfotalea psychrophila LSv54 chromosome I [1205550-1215199]	0	0	0	0
NC_006138:7|Desulfotalea psychrophila LSv54 chromosome I [1577900-1585149]	0	0	0	0
NC_006138:9|Desulfotalea psychrophila LSv54 chromosome I [2291700-2301149]	0	1	0	1
NC_006270:1|Bacillus licheniformis ATCC14580 chromosome I [207950-216899]	0	0	0	0
NC_006270:12|Bacillus licheniformis ATCC14580 chromosome I [1965900-1971149]	0	0	0	0
NC_006270:13|Bacillus licheniformis ATCC14580 chromosome I [1998400-2002399]	0	0	0	0
NC_006270:14|Bacillus licheniformis ATCC14580 chromosome I [2172400-2178099]	0	0	0	0
NC_006270:15|Bacillus licheniformis ATCC14580 chromosome I [2180300-2188149]	0	0	0	0
NC_006270:16|Bacillus licheniformis ATCC14580 chromosome I [2595350-2604449]	0	0	0	0
NC_006270:17|Bacillus licheniformis ATCC14580 chromosome I [2661550-2670799]	0	0	1	1
NC_006270:18|Bacillus licheniformis ATCC14580 chromosome I [2677250-2689399]	0	0	0	0
NC_006270:19|Bacillus licheniformis ATCC14580 chromosome I [2855400-2863599]	0	0	0	0
NC_006270:2|Bacillus licheniformis ATCC14580 chromosome I [571100-585649]	0	0	0	0
NC_006270:21|Bacillus licheniformis ATCC14580 chromosome I [3203100-3210449]	0	0	1	1
NC_006270:22|Bacillus licheniformis ATCC14580 chromosome I [3420850-3431849]	0	0	1	1
NC_006270:23|Bacillus licheniformis ATCC14580 chromosome I [3452150-3458249]	0	0	0	0
NC_006270:24|Bacillus licheniformis ATCC14580 chromosome I [3460650-3467699]	0	0	1	1
NC_006270:25|Bacillus licheniformis ATCC14580 chromosome I [3632250-3642299]	0	0	0	0
NC_006270:28|Bacillus licheniformis ATCC14580 chromosome I [4153050-4175999]	0	0	0	0
NC_006270:3|Bacillus licheniformis ATCC14580 chromosome I [721950-730299]	0	0	0	0
NC_006270:4|Bacillus licheniformis ATCC14580 chromosome I [747550-758099]	0	1	0	1
NC_006270:5|Bacillus licheniformis ATCC14580 chromosome I [926800-936549]	0	0	0	0
NC_006270:6|Bacillus licheniformis ATCC14580 chromosome I [1120650-1141749]	0	0	0	0
NC_006270:7|Bacillus licheniformis ATCC14580 chromosome I [1419800-1430349]	0	0	1	1
NC_006270:8|Bacillus licheniformis ATCC14580 chromosome I [1456850-1462699]	0	0	0	0
NC_006270:9|Bacillus licheniformis ATCC14580 chromosome I [1542000-1548799]	0	0	1	1
NC_006300:1|Mannheimia succiniciproducens MBEL55E chromosome I [65250-73549]	0	1	0	1
NC_006300:4|Mannheimia succiniciproducens MBEL55E chromosome I [485900-491099]	0	0	0	0
NC_006300:5|Mannheimia succiniciproducens MBEL55E chromosome I [604400-618999]	0	0	0	0
NC_006300:7|Mannheimia succiniciproducens MBEL55E chromosome I [1053600-1059999]	0	0	0	0
NC_006300:8|Mannheimia succiniciproducens MBEL55E chromosome I [1145450-1154949]	0	0	0	0
NC_006322:10|Bacillus licheniformis ATCC14580 (DSM13) chromosome I [1542900-1549649]	0	0	1	1
NC_006322:12|Bacillus licheniformis ATCC14580 (DSM13) chromosome I [1947250-1959549]	0	1	0	1
NC_006322:13|Bacillus licheniformis ATCC14580 (DSM13) chromosome I [1966750-1972349]	0	0	0	0
NC_006322:14|Bacillus licheniformis ATCC14580 (DSM13) chromosome I [2000400-2003249]	0	0	0	0
NC_006322:15|Bacillus licheniformis ATCC14580 (DSM13) chromosome I [2174100-2178399]	0	0	0	0
NC_006322:16|Bacillus licheniformis ATCC14580 (DSM13) chromosome I [2181400-2190649]	0	0	0	0
NC_006322:17|Bacillus licheniformis ATCC14580 (DSM13) chromosome I [2596200-2605549]	0	0	0	0
NC_006322:18|Bacillus licheniformis ATCC14580 (DSM13) chromosome I [2662950-2671699]	0	0	1	1
NC_006322:19|Bacillus licheniformis ATCC14580 (DSM13) chromosome I [2678300-2690249]	0	0	0	0
NC_006322:2|Bacillus licheniformis ATCC14580 (DSM13) chromosome I [570950-585349]	0	0	0	0
NC_006322:20|Bacillus licheniformis ATCC14580 (DSM13) chromosome I [2855600-2863799]	0	0	0	0
NC_006322:22|Bacillus licheniformis ATCC14580 (DSM13) chromosome I [3203150-3210649]	0	0	1	1
NC_006322:23|Bacillus licheniformis ATCC14580 (DSM13) chromosome I [3421050-3431749]	0	0	1	1
NC_006322:25|Bacillus licheniformis ATCC14580 (DSM13) chromosome I [3452400-3458799]	0	0	1	1
NC_006322:26|Bacillus licheniformis ATCC14580 (DSM13) chromosome I [3460950-3467549]	0	0	0	0
NC_006322:27|Bacillus licheniformis ATCC14580 (DSM13) chromosome I [3632200-3642799]	0	0	0	0
NC_006322:30|Bacillus licheniformis ATCC14580 (DSM13) chromosome I [4153200-4177149]	0	1	1	1
NC_006322:4|Bacillus licheniformis ATCC14580 (DSM13) chromosome I [721700-730049]	0	0	0	0
NC_006322:5|Bacillus licheniformis ATCC14580 (DSM13) chromosome I [747850-759449]	0	1	0	1
NC_006322:6|Bacillus licheniformis ATCC14580 (DSM13) chromosome I [927800-937399]	0	0	1	1
NC_006322:7|Bacillus licheniformis ATCC14580 (DSM13) chromosome I [1121500-1142599]	0	0	0	0
NC_006322:8|Bacillus licheniformis ATCC14580 (DSM13) chromosome I [1419900-1430399]	0	0	0	0
NC_006322:9|Bacillus licheniformis ATCC14580 (DSM13) chromosome I [1456750-1463549]	0	0	0	0
NC_006347:1|Bacteroides fragilis YCH46 chromosome I [1317950-1328649]	0	0	0	0
NC_006347:2|Bacteroides fragilis YCH46 chromosome I [1677950-1685399]	0	1	0	1
NC_006347:4|Bacteroides fragilis YCH46 chromosome I [2954250-2964999]	0	1	0	1
NC_006347:5|Bacteroides fragilis YCH46 chromosome I [4166100-4175699]	0	0	0	0
NC_006370:1|Photobacterium profundum SS9 chromosome I [245700-250199]	0	0	0	0
NC_006370:2|Photobacterium profundum SS9 chromosome I [1484800-1490549]	0	0	0	0
NC_006370:3|Photobacterium profundum SS9 chromosome I [1704900-1710849]	0	0	0	0
NC_006370:5|Photobacterium profundum SS9 chromosome I [2532100-2537899]	0	0	0	0
NC_006370:6|Photobacterium profundum SS9 chromosome I [3103750-3117849]	0	1	0	1
NC_006370:7|Photobacterium profundum SS9 chromosome I [3131650-3138599]	0	1	0	1
NC_006394:1|Haloarcula marismortui ATCC 43049 plasmid pNG600 [1000-6849]	0	0	0	0
NC_006396:9|Haloarcula marismortui ATCC 43049 chromosome I [2063350-2074899]	0	0	1	1
NC_006510:11|Geobacillus kaustophilus HTA426 chromosome I [1403400-1419549]	0	1	0	1
NC_006510:12|Geobacillus kaustophilus HTA426 chromosome I [1451750-1461999]	0	1	0	1
NC_006510:13|Geobacillus kaustophilus HTA426 chromosome I [1505950-1519399]	0	1	0	1
NC_006510:14|Geobacillus kaustophilus HTA426 chromosome I [1735800-1749399]	0	1	1	1
NC_006510:15|Geobacillus kaustophilus HTA426 chromosome I [1905500-1911499]	0	0	0	0
NC_006510:16|Geobacillus kaustophilus HTA426 chromosome I [1930600-1938199]	0	0	0	0
NC_006510:17|Geobacillus kaustophilus HTA426 chromosome I [1946400-1954299]	0	0	0	0
NC_006510:18|Geobacillus kaustophilus HTA426 chromosome I [2002700-2014449]	0	1	0	1
NC_006510:19|Geobacillus kaustophilus HTA426 chromosome I [2048500-2066099]	0	1	1	1

NC_006510:2|Geobacillus kaustophilus HTA426 chromosome I [314650-338999]	0	1	1	1
NC_006510:20|Geobacillus kaustophilus HTA426 chromosome I [2115500-2131949]	0	1	1	1
NC_006510:21|Geobacillus kaustophilus HTA426 chromosome I [2782700-2789799]	0	1	0	1
NC_006510:22|Geobacillus kaustophilus HTA426 chromosome I [2916550-2924599]	1	1	0	1
NC_006510:23|Geobacillus kaustophilus HTA426 chromosome I [2928150-2939249]	0	1	0	1
NC_006510:24|Geobacillus kaustophilus HTA426 chromosome I [3138500-3155699]	0	1	1	1
NC_006510:25|Geobacillus kaustophilus HTA426 chromosome I [3213750-3220899]	0	0	0	0
NC_006510:26|Geobacillus kaustophilus HTA426 chromosome I [3327000-3344949]	0	1	0	1
NC_006510:27|Geobacillus kaustophilus HTA426 chromosome I [3347900-3367099]	0	1	0	1
NC_006510:3|Geobacillus kaustophilus HTA426 chromosome I [376500-392049]	0	0	0	0
NC_006510:4|Geobacillus kaustophilus HTA426 chromosome I [535950-578449]	1	1	1	1
NC_006510:5|Geobacillus kaustophilus HTA426 chromosome I [594700-630099]	1	1	1	1
NC_006510:6|Geobacillus kaustophilus HTA426 chromosome I [751950-764549]	0	1	0	1
NC_006510:7|Geobacillus kaustophilus HTA426 chromosome I [889550-908299]	0	1	1	1
NC_006510:8|Geobacillus kaustophilus HTA426 chromosome I [912550-927499]	0	1	1	1
NC_006510:9|Geobacillus kaustophilus HTA426 chromosome I [1017550-1039749]	0	1	0	1
NC_006511:1|Salmonella enterica ATCC9150 chromosome I [31300-40949]	0	0	0	0
NC_006511:10|Salmonella enterica ATCC9150 chromosome I [1509200-1535549]	0	1	0	1
NC_006511:12|Salmonella enterica ATCC9150 chromosome I [1658650-1675199]	0	1	0	1
NC_006511:13|Salmonella enterica ATCC9150 chromosome I [1772950-1781949]	0	1	0	1
NC_006511:14|Salmonella enterica ATCC9150 chromosome I [1815950-1823349]	0	0	0	0
NC_006511:15|Salmonella enterica ATCC9150 chromosome I [2094050-2108099]	0	1	0	1
NC_006511:16|Salmonella enterica ATCC9150 chromosome I [2255250-2263249]	0	1	0	1
NC_006511:17|Salmonella enterica ATCC9150 chromosome I [2471800-2480549]	0	1	1	1
NC_006511:18|Salmonella enterica ATCC9150 chromosome I [2483550-2494299]	0	1	0	1
NC_006511:19|Salmonella enterica ATCC9150 chromosome I [2515150-2524799]	0	1	0	1
NC_006511:2|Salmonella enterica ATCC9150 chromosome I [533700-542099]	0	0	0	0
NC_006511:21|Salmonella enterica ATCC9150 chromosome I [2550500-2558849]	0	1	0	1
NC_006511:22|Salmonella enterica ATCC9150 chromosome I [2558250-2567099]	0	1	0	1
NC_006511:27|Salmonella enterica ATCC9150 chromosome I [2738050-2746799]	0	0	0	0
NC_006511:28|Salmonella enterica ATCC9150 chromosome I [2823900-2843499]	0	1	0	1
NC_006511:29|Salmonella enterica ATCC9150 chromosome I [2854200-2861649]	0	0	0	0
NC_006511:32|Salmonella enterica ATCC9150 chromosome I [3083350-3095899]	0	1	0	1
NC_006511:34|Salmonella enterica ATCC9150 chromosome I [3694300-3706049]	0	1	0	1
NC_006511:37|Salmonella enterica ATCC9150 chromosome I [4229100-4239849]	0	1	0	1
NC_006511:38|Salmonella enterica ATCC9150 chromosome I [4245500-4254599]	0	0	0	0
NC_006511:39|Salmonella enterica ATCC9150 chromosome I [4307300-4315399]	0	1	0	1
NC_006511:4|Salmonella enterica ATCC9150 chromosome I [796700-807949]	0	1	0	1
NC_006511:40|Salmonella enterica ATCC9150 chromosome I [4468950-4477999]	0	1	0	1
NC_006511:41|Salmonella enterica ATCC9150 chromosome I [4488500-4500699]	0	0	1	1
NC_006511:5|Salmonella enterica ATCC9150 chromosome I [858550-886299]	0	1	0	1
NC_006511:6|Salmonella enterica ATCC9150 chromosome I [1067750-1090049]	0	1	0	1
NC_006511:8|Salmonella enterica ATCC9150 chromosome I [1278350-1284799]	0	1	0	1
NC_006511:9|Salmonella enterica ATCC9150 chromosome I [1383550-1388399]	0	1	0	1
NC_006512:10|Idiomarina loihiensis L2TR chromosome I [2793000-2809599]	0	1	0	1
NC_006512:2|Idiomarina loihiensis L2TR chromosome I [568200-593949]	0	1	0	1
NC_006513:1|Azoarcus sp EbN1 chromosome I [4283050-4287149]	0	0	0	0
NC_006526:4|Zymomonas mobilis ZM4 chromosome I [1979950-2009049]	0	1	1	1
NC_006582:11|Bacillus clausii KSM-K16 chromosome I [1143600-1157799]	0	1	1	1
NC_006582:12|Bacillus clausii KSM-K16 chromosome I [1302300-1309299]	0	1	0	1
NC_006582:13|Bacillus clausii KSM-K16 chromosome I [1449850-1458749]	0	0	0	0
NC_006582:14|Bacillus clausii KSM-K16 chromosome I [1474100-1484249]	0	0	0	0
NC_006582:17|Bacillus clausii KSM-K16 chromosome I [1532350-1540799]	0	0	0	0
NC_006582:18|Bacillus clausii KSM-K16 chromosome I [2208100-2217899]	0	0	0	0
NC_006582:19|Bacillus clausii KSM-K16 chromosome I [3277050-3283749]	0	0	0	0
NC_006582:2|Bacillus clausii KSM-K16 chromosome I [223550-233799]	0	0	0	0
NC_006582:20|Bacillus clausii KSM-K16 chromosome I [3839000-3853549]	0	1	1	1
NC_006582:21|Bacillus clausii KSM-K16 chromosome I [3952900-3963949]	0	1	1	1
NC_006582:22|Bacillus clausii KSM-K16 chromosome I [4260000-4272849]	0	1	0	1
NC_006582:3|Bacillus clausii KSM-K16 chromosome I [272750-279099]	0	0	0	0
NC_006582:6|Bacillus clausii KSM-K16 chromosome I [658550-666049]	0	0	0	0
NC_006582:7|Bacillus clausii KSM-K16 chromosome I [890900-914349]	0	1	0	1
NC_006582:8|Bacillus clausii KSM-K16 chromosome I [1016000-1031599]	0	0	0	0
NC_006814:1|Lactobacillus acidophilus NCFM chromosome I [1748450-1752549]	0	0	0	0
NC_006856:1|Salmonella enterica subsp. enterica serovar Choleraesuis str. virus [101000-110299]	0	0	0	0
NC_006905:13|Salmonella enterica SC-B67 chromosome I [1152450-1160549]	0	0	0	0
NC_006905:15|Salmonella enterica SC-B67 chromosome I [1347150-1368499]	1	1	1	1
NC_006905:16|Salmonella enterica SC-B67 chromosome I [1511150-1539999]	0	1	0	1
NC_006905:18|Salmonella enterica SC-B67 chromosome I [1729850-1742749]	0	1	0	1
NC_006905:19|Salmonella enterica SC-B67 chromosome I [1772150-1780249]	0	1	0	1
NC_006905:2|Salmonella enterica SC-B67 chromosome I [330300-340099]	0	1	0	1
NC_006905:20|Salmonella enterica SC-B67 chromosome I [1892800-1899749]	0	0	0	0
NC_006905:21|Salmonella enterica SC-B67 chromosome I [1964750-1988749]	0	1	1	1
NC_006905:23|Salmonella enterica SC-B67 chromosome I [2240400-2255349]	0	1	1	1
NC_006905:28|Salmonella enterica SC-B67 chromosome I [2878200-2885649]	0	0	0	0
NC_006905:29|Salmonella enterica SC-B67 chromosome I [2962700-2982449]	0	1	0	1
NC_006905:3|Salmonella enterica SC-B67 chromosome I [376750-396899]	0	0	0	0
NC_006905:30|Salmonella enterica SC-B67 chromosome I [2993450-3001349]	0	0	0	0
NC_006905:33|Salmonella enterica SC-B67 chromosome I [3849350-3860149]	0	1	0	1
NC_006905:35|Salmonella enterica SC-B67 chromosome I [4409750-4419349]	0	1	0	1
NC_006905:36|Salmonella enterica SC-B67 chromosome I [4428150-4437249]	0	0	0	0
NC_006905:37|Salmonella enterica SC-B67 chromosome I [4490950-4500149]	0	1	1	1
NC_006905:38|Salmonella enterica SC-B67 chromosome I [4642450-4668499]	0	1	1	1
NC_006905:5|Salmonella enterica SC-B67 chromosome I [425300-436099]	0	1	0	1
NC_006905:7|Salmonella enterica SC-B67 chromosome I [654500-667999]	0	1	0	1
NC_006905:9|Salmonella enterica SC-B67 chromosome I [949700-960499]	0	1	1	1
NC_006932:2|Brucella abortus biovar1 9-941 chromosome I [266900-280049]	0	0	0	0
NC_006932:3|Brucella abortus biovar1 9-941 chromosome I [287250-294899]	0	1	0	1
NC_006932:4|Brucella abortus biovar1 9-941 chromosome I [534700-557149]	0	1	1	1

NC_006932:5|Brucella abortus biovar1 9-941 chromosome I [734700-741099]	0	0	0	0
NC_006932:6|Brucella abortus biovar1 9-941 chromosome I [794150-801199]	0	1	0	1
NC_006932:7|Brucella abortus biovar1 9-941 chromosome I [954400-960799]	0	0	1	1
NC_006932:8|Brucella abortus biovar1 9-941 chromosome I [961400-971899]	0	1	0	1
NC_006932:9|Brucella abortus biovar1 9-941 chromosome I [1064150-1071299]	0	1	0	1
NC_006933:4|Brucella abortus biovar1 9-941 chromosome II [606000-613999]	0	0	0	0
NC_006933:5|Brucella abortus biovar1 9-941 chromosome II [671900-680399]	0	0	0	0
NC_006933:6|Brucella abortus biovar1 9-941 chromosome II [765900-770249]	0	0	0	0
NC_006933:7|Brucella abortus biovar1 9-941 chromosome II [842600-851549]	0	0	0	0
NC_007005:1|Pseudomonas syringae B728a chromosome I [103500-124349]	1	1	0	1
NC_007005:26|Pseudomonas syringae B728a chromosome I [4520500-4533649]	0	1	1	1
NC_007204:1|Psychrobacter arcticus 273-4 chromosome I [758800-765599]	0	1	0	1
NC_007204:3|Psychrobacter arcticus 273-4 chromosome I [1035050-1042449]	0	0	0	0
NC_007204:5|Psychrobacter arcticus 273-4 chromosome I [1658000-1676249]	0	1	0	1
NC_007298:4|Dechloromonas aromatica RCB chromosome I [781150-794499]	0	1	0	1
NC_007355:1|Methanosarcina barkeri Fusaro chromosome I [48750-54699]	0	0	1	1
NC_007355:2|Methanosarcina barkeri Fusaro chromosome I [1628400-1634399]	0	1	0	1
NC_007355:3|Methanosarcina barkeri Fusaro chromosome I [3650850-3655999]	0	0	0	0
NC_007356:1|Dehalococcoides sp CBDB1 chromosome I [57350-69799]	0	1	0	1
NC_007356:2|Dehalococcoides sp CBDB1 chromosome I [79950-86849]	1	1	1	1
NC_007356:4|Dehalococcoides sp CBDB1 chromosome I [209600-213549]	0	0	0	0
NC_007356:5|Dehalococcoides sp CBDB1 chromosome I [1170650-1181599]	1	1	0	1
NC_007356:6|Dehalococcoides sp CBDB1 chromosome I [1190300-1199799]	0	0	1	1
NC_007356:7|Dehalococcoides sp CBDB1 chromosome I [1224550-1235899]	0	0	0	0
NC_007356:8|Dehalococcoides sp CBDB1 chromosome I [1247000-1253949]	1	0	0	1
NC_007384:1|Shigella sonnei Ss046 chromosome I [19350-27999]	0	1	0	1
NC_007384:12|Shigella sonnei Ss046 chromosome I [1323900-1335999]	0	0	1	1
NC_007384:13|Shigella sonnei Ss046 chromosome I [1360050-1368749]	0	1	1	1
NC_007384:15|Shigella sonnei Ss046 chromosome I [1699650-1713449]	0	1	1	1
NC_007384:16|Shigella sonnei Ss046 chromosome I [1741850-1749899]	0	0	0	0
NC_007384:18|Shigella sonnei Ss046 chromosome I [2007200-2025999]	0	1	1	1
NC_007384:20|Shigella sonnei Ss046 chromosome I [2122550-2128549]	0	1	0	1
NC_007384:21|Shigella sonnei Ss046 chromosome I [2257400-2264799]	0	1	1	1
NC_007384:24|Shigella sonnei Ss046 chromosome I [2591700-2609249]	0	1	0	1
NC_007384:28|Shigella sonnei Ss046 chromosome I [3068000-3072349]	0	0	0	0
NC_007384:29|Shigella sonnei Ss046 chromosome I [3154100-3168049]	0	1	1	1
NC_007384:3|Shigella sonnei Ss046 chromosome I [250150-256599]	0	1	1	1
NC_007384:30|Shigella sonnei Ss046 chromosome I [3336950-3346599]	0	0	0	0
NC_007384:32|Shigella sonnei Ss046 chromosome I [3743650-3755499]	0	1	1	1
NC_007384:33|Shigella sonnei Ss046 chromosome I [3899850-3907299]	0	0	0	0
NC_007384:34|Shigella sonnei Ss046 chromosome I [3923750-3928299]	0	0	0	0
NC_007384:35|Shigella sonnei Ss046 chromosome I [3961300-3970049]	0	1	0	1
NC_007384:39|Shigella sonnei Ss046 chromosome I [4210900-4216849]	0	0	0	0
NC_007384:8|Shigella sonnei Ss046 chromosome I [1089550-1103349]	0	0	0	0
NC_007384:9|Shigella sonnei Ss046 chromosome I [1127600-1131649]	0	1	0	1
NC_007413:1|Anabaena variabilis ATCC29413 chromosome I [1263450-1266299]	0	0	0	0
NC_007413:2|Anabaena variabilis ATCC29413 chromosome I [1590200-1594449]	0	0	0	0
NC_007413:3|Anabaena variabilis ATCC29413 chromosome I [2972650-2975999]	0	0	0	0
NC_007426:2|Natronomonas pharaonis DSM2160 chromosome I [1869050-1881799]	0	1	0	1
NC_007426:3|Natronomonas pharaonis DSM2160 chromosome I [1892500-1900899]	0	1	0	1
NC_007481:1|Pseudoalteromonas haloplanktis TAC125 chromosome I [1872350-1887199]	0	0	0	0
NC_007498:13|Pelobacter carbinolicus DSM2380 chromosome I [1783000-1790649]	0	0	0	0
NC_007498:14|Pelobacter carbinolicus DSM2380 chromosome I [1791300-1801349]	0	0	0	0
NC_007498:17|Pelobacter carbinolicus DSM2380 chromosome I [2092800-2115349]	0	1	0	1
NC_007498:18|Pelobacter carbinolicus DSM2380 chromosome I [2481600-2500249]	0	0	0	0
NC_007503:1|Carboxydothermus hydrogenoformans Z-2901 chromosome I [862900-872049]	0	0	0	0
NC_007503:2|Carboxydothermus hydrogenoformans Z-2901 chromosome I [924650-932249]	0	0	0	0
NC_007503:3|Carboxydothermus hydrogenoformans Z-2901 chromosome I [1152850-1167199]	0	0	0	0
NC_007503:4|Carboxydothermus hydrogenoformans Z-2901 chromosome I [1168400-1174099]	0	0	0	0
NC_007503:5|Carboxydothermus hydrogenoformans Z-2901 chromosome I [1513850-1517149]	0	0	0	0
NC_007503:6|Carboxydothermus hydrogenoformans Z-2901 chromosome I [1537200-1542549]	0	0	0	0
NC_007503:7|Carboxydothermus hydrogenoformans Z-2901 chromosome I [1685400-1699049]	0	0	0	0
NC_007503:8|Carboxydothermus hydrogenoformans Z-2901 chromosome I [1938500-1942299]	0	0	0	0
NC_007503:9|Carboxydothermus hydrogenoformans Z-2901 chromosome I [2359100-2365849]	0	0	0	0
NC_007512:15|Pelodictyon luteolum DSM273 chromosome I [2036350-2085899]	0	1	1	1
NC_007512:5|Pelodictyon luteolum DSM273 chromosome I [799900-807449]	0	0	0	0
NC_007512:6|Pelodictyon luteolum DSM273 chromosome I [871800-889549]	0	0	0	0
NC_007514:1|Chlorobium chlorochromatii CaD3 chromosome I [338500-347749]	0	0	0	0
NC_007514:10|Chlorobium chlorochromatii CaD3 chromosome I [1314850-1322749]	0	0	0	0
NC_007514:11|Chlorobium chlorochromatii CaD3 chromosome I [1481350-1488699]	0	0	0	0
NC_007514:12|Chlorobium chlorochromatii CaD3 chromosome I [1823550-1829949]	0	0	0	0
NC_007514:13|Chlorobium chlorochromatii CaD3 chromosome I [1849300-1855249]	0	0	0	0
NC_007514:14|Chlorobium chlorochromatii CaD3 chromosome I [1859900-1865799]	0	0	0	0
NC_007514:15|Chlorobium chlorochromatii CaD3 chromosome I [1937150-1950549]	0	0	0	0
NC_007514:16|Chlorobium chlorochromatii CaD3 chromosome I [2100500-2106299]	0	0	0	0
NC_007514:3|Chlorobium chlorochromatii CaD3 chromosome I [589300-592949]	0	0	0	0
NC_007514:4|Chlorobium chlorochromatii CaD3 chromosome I [698000-703399]	0	0	0	0
NC_007514:5|Chlorobium chlorochromatii CaD3 chromosome I [914300-950899]	0	0	0	0
NC_007514:6|Chlorobium chlorochromatii CaD3 chromosome I [1050150-1057399]	0	0	0	0
NC_007514:7|Chlorobium chlorochromatii CaD3 chromosome I [1066100-1071749]	0	0	0	0
NC_007514:8|Chlorobium chlorochromatii CaD3 chromosome I [1241000-1246499]	0	0	0	0
NC_007514:9|Chlorobium chlorochromatii CaD3 chromosome I [1282750-1287549]	0	0	0	0
NC_007517:13|Geobacter metallireducens GS-15 chromosome I [2275450-2289649]	0	0	1	1
NC_007517:16|Geobacter metallireducens GS-15 chromosome I [2525100-2551149]	0	0	1	1
NC_007517:5|Geobacter metallireducens GS-15 chromosome I [1474150-1493599]	0	0	1	1
NC_007517:7|Geobacter metallireducens GS-15 chromosome I [1683550-1696499]	0	0	0	0
NC_007575:1|Thiomicrospira denitrificans ATCC 33889 chromosome I [154900-159749]	0	0	0	0
NC_007576:1|Lactobacillus sakei subsp. sakei 23K chromosome I [137900-145149]	0	1	0	1
NC_007576:2|Lactobacillus sakei subsp. sakei 23K chromosome I [574200-579899]	0	0	0	0

NC_007576:3|Lactobacillus sakei subsp. sakei 23K chromosome I [1475050-1486549]	0	1	0	1
NC_007576:4|Lactobacillus sakei subsp. sakei 23K chromosome I [1551050-1562449]	0	0	0	0
NC_007576:5|Lactobacillus sakei subsp. sakei 23K chromosome I [1701900-1706949]	0	0	0	0
NC_007606:1|Shigella dysenteriae Sd197 chromosome I [234500-240999]	0	0	0	0
NC_007606:16|Shigella dysenteriae Sd197 chromosome I [1517350-1529249]	0	1	1	1
NC_007606:17|Shigella dysenteriae Sd197 chromosome I [1603750-1611049]	0	0	0	0
NC_007606:19|Shigella dysenteriae Sd197 chromosome I [2003850-2017049]	0	1	0	1
NC_007606:24|Shigella dysenteriae Sd197 chromosome I [2353950-2372699]	0	1	1	1
NC_007606:26|Shigella dysenteriae Sd197 chromosome I [3068200-3075249]	0	0	0	0
NC_007606:31|Shigella dysenteriae Sd197 chromosome I [3787650-3797299]	0	1	0	1
NC_007613:13|Shigella boydii Sb227 chromosome I [1536950-1551399]	0	1	0	1
NC_007613:18|Shigella boydii Sb227 chromosome I [2379400-2397449]	0	1	0	1
NC_007613:20|Shigella boydii Sb227 chromosome I [2703450-2710349]	0	1	0	1
NC_007613:21|Shigella boydii Sb227 chromosome I [2900750-2905549]	0	0	0	0
NC_007613:22|Shigella boydii Sb227 chromosome I [2985850-2992049]	0	1	0	1
NC_007613:23|Shigella boydii Sb227 chromosome I [3472400-3480399]	0	0	0	0
NC_007613:24|Shigella boydii Sb227 chromosome I [3494400-3504049]	0	1	0	1
NC_007613:25|Shigella boydii Sb227 chromosome I [3605000-3609849]	0	0	0	0
NC_007613:26|Shigella boydii Sb227 chromosome I [3640750-3651949]	0	1	0	1
NC_007613:28|Shigella boydii Sb227 chromosome I [4119350-4126099]	0	1	0	1
NC_007613:32|Shigella boydii Sb227 chromosome I [4239000-4248249]	0	0	0	0
NC_007613:34|Shigella boydii Sb227 chromosome I [4407300-4419149]	0	1	0	1
NC_007613:7|Shigella boydii Sb227 chromosome I [1044600-1057549]	0	1	0	1
NC_007613:8|Shigella boydii Sb227 chromosome I [1165700-1172149]	0	0	0	0
NC_007618:2|Brucella melitensis biovar Abortus 2308 chromosome I [263350-276399]	0	0	0	0
NC_007618:3|Brucella melitensis biovar Abortus 2308 chromosome I [283550-290899]	0	1	0	1
NC_007618:4|Brucella melitensis biovar Abortus 2308 chromosome I [531600-553449]	0	1	1	1
NC_007618:5|Brucella melitensis biovar Abortus 2308 chromosome I [728750-738799]	0	1	0	1
NC_007618:6|Brucella melitensis biovar Abortus 2308 chromosome I [789500-797449]	0	0	0	0
NC_007618:7|Brucella melitensis biovar Abortus 2308 chromosome I [951600-957499]	0	1	1	1
NC_007618:8|Brucella melitensis biovar Abortus 2308 chromosome I [957200-968799]	0	0	0	0
NC_007618:9|Brucella melitensis biovar Abortus 2308 chromosome I [1061300-1069249]	0	1	0	1
NC_007624:1|Brucella melitensis biovar Abortus 2308 chromosome II [128000-133099]	0	0	0	0
NC_007624:4|Brucella melitensis biovar Abortus 2308 chromosome II [606350-613399]	0	0	0	0
NC_007624:5|Brucella melitensis biovar Abortus 2308 chromosome II [672200-680349]	0	0	1	1
NC_007624:7|Brucella melitensis biovar Abortus 2308 chromosome II [843750-852249]	0	0	0	0
NC_007644:1|Moorella thermoacetica ATCC 39073 chromosome I [10050-15699]	0	0	0	0
NC_007644:10|Moorella thermoacetica ATCC 39073 chromosome I [651200-659549]	0	0	0	0
NC_007644:11|Moorella thermoacetica ATCC 39073 chromosome I [690050-706799]	0	0	0	0
NC_007644:13|Moorella thermoacetica ATCC 39073 chromosome I [785650-795899]	0	0	0	0
NC_007644:15|Moorella thermoacetica ATCC 39073 chromosome I [1610950-1617499]	0	0	0	0
NC_007644:16|Moorella thermoacetica ATCC 39073 chromosome I [1643250-1659349]	0	0	1	1
NC_007644:17|Moorella thermoacetica ATCC 39073 chromosome I [1697100-1707799]	0	0	0	0
NC_007644:2|Moorella thermoacetica ATCC 39073 chromosome I [166250-180299]	0	0	0	0
NC_007644:20|Moorella thermoacetica ATCC 39073 chromosome I [2211400-2222749]	0	0	0	0
NC_007644:22|Moorella thermoacetica ATCC 39073 chromosome I [2461050-2471349]	0	0	1	1
NC_007644:3|Moorella thermoacetica ATCC 39073 chromosome I [284450-306899]	0	0	1	1
NC_007644:4|Moorella thermoacetica ATCC 39073 chromosome I [317100-331099]	0	0	0	0
NC_007644:5|Moorella thermoacetica ATCC 39073 chromosome I [365800-374199]	0	0	1	1
NC_007644:6|Moorella thermoacetica ATCC 39073 chromosome I [374750-402249]	0	0	0	0
NC_007644:7|Moorella thermoacetica ATCC 39073 chromosome I [421100-434849]	0	0	1	1
NC_007644:8|Moorella thermoacetica ATCC 39073 chromosome I [474750-482099]	0	0	0	0
NC_007644:9|Moorella thermoacetica ATCC 39073 chromosome I [634050-640999]	0	0	0	0
NC_007645:16|Hahella chejuensis KCTC2396 chromosome I [2297650-2309249]	0	1	0	1
NC_007645:18|Hahella chejuensis KCTC2396 chromosome I [2413400-2424199]	0	1	1	1
NC_007645:19|Hahella chejuensis KCTC2396 chromosome I [2430200-2468149]	0	1	0	1
NC_007645:2|Hahella chejuensis KCTC2396 chromosome I [609250-616399]	0	1	1	1
NC_007645:21|Hahella chejuensis KCTC2396 chromosome I [2783450-2822399]	0	1	0	1
NC_007645:4|Hahella chejuensis KCTC2396 chromosome I [1039050-1058649]	0	1	0	1
NC_007645:48|Hahella chejuensis KCTC2396 chromosome I [5402150-5423649]	0	1	0	1
NC_007645:60|Hahella chejuensis KCTC2396 chromosome I [6650400-6667399]	0	1	0	1
NC_007712:11|Sodalis glossinidius morsitans chromosome I [1606000-1623399]	0	1	0	1
NC_007712:15|Sodalis glossinidius morsitans chromosome I [1908700-1918799]	0	0	0	0
NC_007712:16|Sodalis glossinidius morsitans chromosome I [1960200-1981549]	0	1	1	1
NC_007712:19|Sodalis glossinidius morsitans chromosome I [2050800-2071899]	0	1	1	1
NC_007712:23|Sodalis glossinidius morsitans chromosome I [2527850-2542799]	0	0	0	0
NC_007712:30|Sodalis glossinidius morsitans chromosome I [3095550-3110649]	0	1	0	1
NC_007712:32|Sodalis glossinidius morsitans chromosome I [3166000-3177349]	0	0	0	0
NC_007712:37|Sodalis glossinidius morsitans chromosome I [3633250-3645849]	0	0	0	0
NC_007712:38|Sodalis glossinidius morsitans chromosome I [3707150-3716649]	0	0	0	0
NC_007712:6|Sodalis glossinidius morsitans chromosome I [1186850-1242349]	0	1	1	1
NC_007712:7|Sodalis glossinidius morsitans chromosome I [1265100-1281999]	0	1	0	1
NC_007712:9|Sodalis glossinidius morsitans chromosome I [1520400-1534949]	0	1	0	1
NC_007761:3|Rhizobium etli CFN 42 chromosome I [803000-811799]	0	1	0	1
NC_007761:6|Rhizobium etli CFN 42 chromosome I [3949650-3957749]	0	0	0	0
NC_007775:2|Synechococcus sp. JA-3-3Ab chromosome I [1007450-1013049]	0	1	0	1
NC_007776:10|Synechococcus sp. JA-2-3B'a(2-13) chromosome I [1801950-1811999]	0	0	0	0
NC_007776:12|Synechococcus sp. JA-2-3B'a(2-13) chromosome I [2101550-2107549]	0	0	0	0
NC_007776:13|Synechococcus sp. JA-2-3B'a(2-13) chromosome I [2151050-2157349]	0	0	0	0
NC_007776:15|Synechococcus sp. JA-2-3B'a(2-13) chromosome I [2466600-2475099]	0	0	0	0
NC_007776:16|Synechococcus sp. JA-2-3B'a(2-13) chromosome I [2637300-2642849]	0	0	0	0
NC_007776:18|Synechococcus sp. JA-2-3B'a(2-13) chromosome I [2834150-2840949]	0	0	0	0
NC_007776:2|Synechococcus sp. JA-2-3B'a(2-13) chromosome I [821800-827999]	0	0	0	0
NC_007776:3|Synechococcus sp. JA-2-3B'a(2-13) chromosome I [866700-871249]	0	0	0	0
NC_007776:5|Synechococcus sp. JA-2-3B'a(2-13) chromosome I [1296700-1302149]	0	0	0	0
NC_007776:6|Synechococcus sp. JA-2-3B'a(2-13) chromosome I [1327350-1333999]	0	0	0	0
NC_007776:8|Synechococcus sp. JA-2-3B'a(2-13) chromosome I [1404100-1408849]	0	0	0	0
NC_007796:10|Methanospirillum hungatei JF-1 chromosome I [3355700-3366249]	0	1	0	1
NC_007796:11|Methanospirillum hungatei JF-1 chromosome I [3389400-3397949]	0	0	1	1

NC_007796:12|Methanospirillum hungatei JF-1 chromosome I [3426950-3440399]	0	1	0	1
NC_007796:2|Methanospirillum hungatei JF-1 chromosome I [423750-437049]	0	1	0	1
NC_007796:4|Methanospirillum hungatei JF-1 chromosome I [1734800-1747999]	0	1	0	1
NC_007796:5|Methanospirillum hungatei JF-1 chromosome I [2291850-2299249]	0	0	0	0
NC_007796:6|Methanospirillum hungatei JF-1 chromosome I [2391500-2409249]	0	1	1	1
NC_007796:7|Methanospirillum hungatei JF-1 chromosome I [2622250-2629299]	0	1	0	1
NC_007796:8|Methanospirillum hungatei JF-1 chromosome I [2972100-2985399]	0	0	0	0
NC_007796:9|Methanospirillum hungatei JF-1 chromosome I [3120900-3130149]	0	1	0	1
NC_007802:1|Jannaschia sp. CCS1 chromosome I [1098800-1106299]	0	0	0	0
NC_007802:2|Jannaschia sp. CCS1 chromosome I [1402000-1405799]	0	0	0	0
NC_007907:12|Desulfitobacterium hafniense Y51 chromosome I [2327500-2337899]	0	1	0	1
NC_007907:14|Desulfitobacterium hafniense Y51 chromosome I [2886100-2896299]	0	1	0	1
NC_007907:15|Desulfitobacterium hafniense Y51 chromosome I [3000550-3019249]	0	1	0	1
NC_007907:16|Desulfitobacterium hafniense Y51 chromosome I [3235150-3249299]	0	1	0	1
NC_007907:17|Desulfitobacterium hafniense Y51 chromosome I [3477000-3483099]	0	0	0	0
NC_007907:18|Desulfitobacterium hafniense Y51 chromosome I [3727750-3751699]	0	1	0	1
NC_007907:19|Desulfitobacterium hafniense Y51 chromosome I [3878250-3887399]	0	1	0	1
NC_007907:2|Desulfitobacterium hafniense Y51 chromosome I [769650-779849]	0	1	0	1
NC_007907:20|Desulfitobacterium hafniense Y51 chromosome I [4505900-4512599]	0	0	0	0
NC_007907:23|Desulfitobacterium hafniense Y51 chromosome I [4930950-4940349]	0	1	0	1
NC_007907:25|Desulfitobacterium hafniense Y51 chromosome I [4961650-4980599]	0	1	0	1
NC_007907:27|Desulfitobacterium hafniense Y51 chromosome I [5071350-5078849]	0	0	0	0
NC_007907:29|Desulfitobacterium hafniense Y51 chromosome I [5108600-5149049]	0	1	1	1
NC_007907:3|Desulfitobacterium hafniense Y51 chromosome I [816050-829449]	0	1	0	1
NC_007907:30|Desulfitobacterium hafniense Y51 chromosome I [5191750-5208449]	0	1	0	1
NC_007907:31|Desulfitobacterium hafniense Y51 chromosome I [5223250-5232199]	0	0	0	0
NC_007907:32|Desulfitobacterium hafniense Y51 chromosome I [5237300-5250899]	0	0	0	0
NC_007907:35|Desulfitobacterium hafniense Y51 chromosome I [5302550-5309149]	0	0	0	0
NC_007907:36|Desulfitobacterium hafniense Y51 chromosome I [5436600-5448549]	0	0	1	1
NC_007907:4|Desulfitobacterium hafniense Y51 chromosome I [832900-839549]	0	1	0	1
NC_007907:6|Desulfitobacterium hafniense Y51 chromosome I [965100-986849]	0	1	1	1
NC_007907:7|Desulfitobacterium hafniense Y51 chromosome I [985950-1027649]	0	1	1	1
NC_007912:1|Saccharophagus degradans 2-40 chromosome I [178200-191349]	0	0	0	0
NC_007912:5|Saccharophagus degradans 2-40 chromosome I [2706550-2721699]	0	0	0	0
NC_007912:7|Saccharophagus degradans 2-40 chromosome I [3836100-3844199]	0	0	0	0
NC_007912:9|Saccharophagus degradans 2-40 chromosome I [4402800-4406649]	0	0	0	0
NC_007946:10|Escherichia coli UTI89 chromosome I [1121700-1131699]	0	1	0	1
NC_007946:11|Escherichia coli UTI89 chromosome I [1245100-1252149]	0	1	0	1
NC_007946:12|Escherichia coli UTI89 chromosome I [1252500-1259599]	0	0	0	0
NC_007946:13|Escherichia coli UTI89 chromosome I [1283000-1299899]	0	1	0	1
NC_007946:15|Escherichia coli UTI89 chromosome I [1527000-1532099]	0	0	0	0
NC_007946:17|Escherichia coli UTI89 chromosome I [1634000-1664299]	0	1	0	1
NC_007946:19|Escherichia coli UTI89 chromosome I [2032450-2037399]	0	0	0	0
NC_007946:20|Escherichia coli UTI89 chromosome I [2058850-2067299]	0	0	0	0
NC_007946:23|Escherichia coli UTI89 chromosome I [2169800-2185999]	0	1	0	1
NC_007946:24|Escherichia coli UTI89 chromosome I [2235250-2249099]	0	1	0	1
NC_007946:25|Escherichia coli UTI89 chromosome I [2302050-2310149]	0	1	0	1
NC_007946:26|Escherichia coli UTI89 chromosome I [2326750-2332999]	0	1	0	1
NC_007946:27|Escherichia coli UTI89 chromosome I [2595300-2606099]	0	1	1	1
NC_007946:28|Escherichia coli UTI89 chromosome I [2640250-2655299]	0	1	0	1
NC_007946:3|Escherichia coli UTI89 chromosome I [262900-271149]	0	1	0	1
NC_007946:32|Escherichia coli UTI89 chromosome I [3131750-3137249]	0	0	0	0
NC_007946:33|Escherichia coli UTI89 chromosome I [3294700-3305699]	0	1	0	1
NC_007946:35|Escherichia coli UTI89 chromosome I [3815050-3825049]	0	1	0	1
NC_007946:36|Escherichia coli UTI89 chromosome I [3858550-3864899]	0	1	0	1
NC_007946:38|Escherichia coli UTI89 chromosome I [4054650-4061499]	0	1	0	1
NC_007946:39|Escherichia coli UTI89 chromosome I [4408900-4414599]	0	1	0	1
NC_007946:4|Escherichia coli UTI89 chromosome I [294250-314199]	0	1	1	1
NC_007946:40|Escherichia coli UTI89 chromosome I [4781000-4792349]	0	1	0	1
NC_007946:41|Escherichia coli UTI89 chromosome I [4793350-4808299]	1	1	0	1
NC_007946:42|Escherichia coli UTI89 chromosome I [4814300-4841999]	0	1	0	1
NC_007946:43|Escherichia coli UTI89 chromosome I [4882400-4889199]	0	0	0	0
NC_007946:44|Escherichia coli UTI89 chromosome I [4893100-4909449]	0	1	1	1
NC_007946:45|Escherichia coli UTI89 chromosome I [4933200-4937549]	0	0	0	0
NC_007946:5|Escherichia coli UTI89 chromosome I [319450-326649]	0	1	0	1
NC_007946:6|Escherichia coli UTI89 chromosome I [345700-349649]	0	0	0	0
NC_007946:8|Escherichia coli UTI89 chromosome I [1074250-1088249]	0	1	1	1
NC_007947:10|Methylobacillus flagellatus KT chromosome I [1342050-1356199]	0	1	0	1
NC_007947:14|Methylobacillus flagellatus KT chromosome I [2142150-2171449]	0	1	0	1
NC_007947:18|Methylobacillus flagellatus KT chromosome I [2451500-2481399]	0	0	0	0
NC_007954:12|Shewanella denitrificans OS217 chromosome I [1828200-1833449]	0	0	1	1
NC_007954:14|Shewanella denitrificans OS217 chromosome I [3177400-3199999]	0	1	0	1
NC_007954:18|Shewanella denitrificans OS217 chromosome I [4288100-4298599]	0	1	1	1
NC_007954:4|Shewanella denitrificans OS217 chromosome I [340850-352649]	0	1	1	1
NC_007954:9|Shewanella denitrificans OS217 chromosome I [1498000-1506699]	0	0	0	0
NC_007955:1|Methanococcoides burtonii DSM 6242 chromosome I [960100-962649]	0	0	0	0
NC_007955:2|Methanococcoides burtonii DSM 6242 chromosome I [2127900-2131399]	0	0	0	0
NC_007955:3|Methanococcoides burtonii DSM 6242 chromosome I [751000-757199]	0	1	0	1
NC_007958:1|Rhodopseudomonas palustris BisB5 [42100-44649]	0	0	0	0
NC_007969:1|Psychrobacter cryohalolentis K5 chromosome I [732400-742449]	0	1	0	1
NC_007969:2|Psychrobacter cryohalolentis K5 chromosome I [1079750-1106399]	0	1	0	1
NC_007969:4|Psychrobacter cryohalolentis K5 chromosome I [1654750-1659299]	0	1	0	1
NC_008009:1|Acidobacteria bacterium Ellin345 chromosome I [447700-460649]	0	1	0	1
NC_008009:10|Acidobacteria bacterium Ellin345 chromosome I [3153800-3157999]	0	0	0	0
NC_008009:11|Acidobacteria bacterium Ellin345 chromosome I [3429700-3432799]	0	0	0	0
NC_008009:18|Acidobacteria bacterium Ellin345 chromosome I [4914750-4920999]	0	0	0	0
NC_008009:19|Acidobacteria bacterium Ellin345 chromosome I [4928700-4931849]	0	0	0	0
NC_008009:3|Acidobacteria bacterium Ellin345 chromosome I [1271600-1288999]	0	1	0	1
NC_008009:6|Acidobacteria bacterium Ellin345 chromosome I [2284900-2288799]	0	0	0	0

NC_008009:7|Acidobacteria bacterium Ellin345 chromosome I [2437550-2445499]	0	1	0	1
NC_008009:8|Acidobacteria bacterium Ellin345 chromosome I [2447450-2455049]	0	1	0	1
NC_008009:9|Acidobacteria bacterium Ellin345 chromosome I [3141250-3148049]	0	1	0	1
NC_008021:5|Streptococcus pyogenes MGAS9429 chromosome I [559050-565949]	0	0	0	0
NC_008022:5|Streptococcus pyogenes MGAS10270 chromosome I [560600-566549]	0	0	0	0
NC_008027:3|Pseudomonas entomophila L48 chromosome I [1567100-1575249]	0	1	0	1
NC_008054:1|Lactobacillus delbrueckii subsp. bulgaricus ATCC 11842 chromosome I [211050-229149]	0	1	0	1
NC_008054:10|Lactobacillus delbrueckii subsp. bulgaricus ATCC 11842 chromosome I [1505600-1517299]	0	1	0	1
NC_008054:11|Lactobacillus delbrueckii subsp. bulgaricus ATCC 11842 chromosome I [1623450-1633449]	0	1	0	1
NC_008054:12|Lactobacillus delbrueckii subsp. bulgaricus ATCC 11842 chromosome I [1652200-1677849]	0	1	0	1
NC_008054:16|Lactobacillus delbrueckii subsp. bulgaricus ATCC 11842 chromosome I [1849250-1854749]	0	0	0	0
NC_008054:2|Lactobacillus delbrueckii subsp. bulgaricus ATCC 11842 chromosome I [252650-259849]	0	0	0	0
NC_008054:3|Lactobacillus delbrueckii subsp. bulgaricus ATCC 11842 chromosome I [275800-283499]	0	0	0	0
NC_008054:5|Lactobacillus delbrueckii subsp. bulgaricus ATCC 11842 chromosome I [933250-949449]	0	1	0	1
NC_008054:6|Lactobacillus delbrueckii subsp. bulgaricus ATCC 11842 chromosome I [1034000-1048499]	0	1	0	1
NC_008054:7|Lactobacillus delbrueckii subsp. bulgaricus ATCC 11842 chromosome I [1121100-1130349]	0	0	0	0
NC_008054:9|Lactobacillus delbrueckii subsp. bulgaricus ATCC 11842 chromosome I [1445700-1451099]	0	0	0	0
NC_008228:1|Pseudoalteromonas atlantica T6c chromosome I [377750-383099]	0	1	0	1
NC_008228:11|Pseudoalteromonas atlantica T6c chromosome I [4918650-4922449]	0	0	0	0
NC_008228:2|Pseudoalteromonas atlantica T6c chromosome I [775350-778699]	0	0	1	1
NC_008228:4|Pseudoalteromonas atlantica T6c chromosome I [1073750-1078599]	0	0	0	0
NC_008228:8|Pseudoalteromonas atlantica T6c chromosome I [3684400-3691999]	0	0	0	0
NC_008228:9|Pseudoalteromonas atlantica T6c chromosome I [4313100-4318549]	0	0	0	0
NC_008253:10|Escherichia coli 536 chromosome I [1081700-1088699]	0	1	0	1
NC_008253:11|Escherichia coli 536 chromosome I [1198450-1204649]	0	0	0	0
NC_008253:12|Escherichia coli 536 chromosome I [1230300-1246599]	0	1	0	1
NC_008253:13|Escherichia coli 536 chromosome I [1429150-1435349]	0	0	0	0
NC_008253:14|Escherichia coli 536 chromosome I [1537450-1548999]	0	1	0	1
NC_008253:15|Escherichia coli 536 chromosome I [1911650-1915549]	0	0	0	0
NC_008253:16|Escherichia coli 536 chromosome I [1937600-1969549]	1	1	1	1
NC_008253:19|Escherichia coli 536 chromosome I [2071900-2085849]	0	1	0	1
NC_008253:20|Escherichia coli 536 chromosome I [2138500-2152299]	0	1	0	1
NC_008253:21|Escherichia coli 536 chromosome I [2201550-2209899]	0	1	0	1
NC_008253:22|Escherichia coli 536 chromosome I [2225550-2233299]	0	1	0	1
NC_008253:23|Escherichia coli 536 chromosome I [2502450-2517099]	0	1	0	1
NC_008253:24|Escherichia coli 536 chromosome I [2832650-2843199]	0	1	1	1
NC_008253:26|Escherichia coli 536 chromosome I [2971300-2977049]	0	1	0	1
NC_008253:27|Escherichia coli 536 chromosome I [3126050-3133699]	1	1	1	1
NC_008253:3|Escherichia coli 536 chromosome I [261900-270399]	0	1	0	1
NC_008253:31|Escherichia coli 536 chromosome I [3182850-3197099]	0	1	0	1
NC_008253:32|Escherichia coli 536 chromosome I [3668600-3678749]	0	1	0	1
NC_008253:33|Escherichia coli 536 chromosome I [3900600-3907549]	0	1	0	1
NC_008253:34|Escherichia coli 536 chromosome I [3945300-3954349]	0	1	1	1
NC_008253:35|Escherichia coli 536 chromosome I [3957750-3967799]	0	1	0	1
NC_008253:37|Escherichia coli 536 chromosome I [3975100-3986749]	1	0	0	1
NC_008253:38|Escherichia coli 536 chromosome I [3989250-4001449]	1	1	0	1
NC_008253:4|Escherichia coli 536 chromosome I [300350-319349]	0	1	0	1
NC_008253:40|Escherichia coli 536 chromosome I [4331000-4336999]	0	1	0	1
NC_008253:41|Escherichia coli 536 chromosome I [4739800-4748749]	0	1	0	1
NC_008253:42|Escherichia coli 536 chromosome I [4750300-4755699]	0	1	0	1
NC_008253:43|Escherichia coli 536 chromosome I [4757000-4780599]	1	0	1	1
NC_008253:44|Escherichia coli 536 chromosome I [4823350-4829699]	0	1	0	1
NC_008253:45|Escherichia coli 536 chromosome I [4833600-4848749]	0	1	1	1
NC_008253:6|Escherichia coli 536 chromosome I [340000-349899]	0	1	0	1
NC_008253:7|Escherichia coli 536 chromosome I [357750-370899]	0	1	0	1
NC_008253:8|Escherichia coli 536 chromosome I [376300-383349]	0	1	0	1
NC_008253:9|Escherichia coli 536 chromosome I [402400-407049]	0	0	0	0
NC_008255:1|Cytophaga hutchinsonii ATCC 33406 chromosome I [47700-61799]	0	1	0	1
NC_008255:2|Cytophaga hutchinsonii ATCC 33406 chromosome I [913750-920099]	0	0	0	0
NC_008255:3|Cytophaga hutchinsonii ATCC 33406 chromosome I [1349500-1362799]	0	1	0	1
NC_008255:4|Cytophaga hutchinsonii ATCC 33406 chromosome I [1719600-1729599]	0	0	0	0
NC_008255:5|Cytophaga hutchinsonii ATCC 33406 chromosome I [2848800-2855349]	0	0	0	0
NC_008255:6|Cytophaga hutchinsonii ATCC 33406 chromosome I [3304900-3311399]	0	0	0	0
NC_008255:7|Cytophaga hutchinsonii ATCC 33406 chromosome I [3365500-3396899]	0	1	1	1
NC_008258:1|Shigella flexneri 5 8401 chromosome I [271450-288149]	0	1	0	1
NC_008258:14|Shigella flexneri 5 8401 chromosome I [1754600-1763649]	0	1	0	1
NC_008258:16|Shigella flexneri 5 8401 chromosome I [1937400-1951499]	0	1	0	1
NC_008258:17|Shigella flexneri 5 8401 chromosome I [2014050-2020199]	0	0	0	0
NC_008258:18|Shigella flexneri 5 8401 chromosome I [2042550-2055199]	0	1	1	1
NC_008258:2|Shigella flexneri 5 8401 chromosome I [406050-417099]	0	1	0	1
NC_008258:21|Shigella flexneri 5 8401 chromosome I [2104900-2119949]	0	1	0	1
NC_008258:22|Shigella flexneri 5 8401 chromosome I [2192850-2200999]	0	0	0	0
NC_008258:23|Shigella flexneri 5 8401 chromosome I [2461750-2468399]	0	0	1	1
NC_008258:24|Shigella flexneri 5 8401 chromosome I [2474350-2492099]	0	1	1	1
NC_008258:28|Shigella flexneri 5 8401 chromosome I [3229500-3235999]	0	1	0	1
NC_008258:29|Shigella flexneri 5 8401 chromosome I [3553150-3571749]	0	1	1	1
NC_008258:30|Shigella flexneri 5 8401 chromosome I [3585700-3598349]	0	1	1	1
NC_008258:33|Shigella flexneri 5 8401 chromosome I [3964400-3972349]	1	1	0	1
NC_008258:34|Shigella flexneri 5 8401 chromosome I [4001200-4011099]	0	1	0	1
NC_008258:35|Shigella flexneri 5 8401 chromosome I [4348750-4358849]	0	1	0	1
NC_008258:9|Shigella flexneri 5 8401 chromosome I [1094400-1100299]	0	0	0	0
NC_008260:3|Alcanivorax borkumensis SK2 chromosome I [233450-248549]	0	0	1	1
NC_008260:9|Alcanivorax borkumensis SK2 chromosome I [1019550-1032049]	0	1	0	1
NC_008321:12|Shewanella sp. MR-4 chromosome I [1640500-1648499]	0	1	0	1
NC_008321:17|Shewanella sp. MR-4 chromosome I [2041300-2046649]	1	0	0	1
NC_008321:2|Shewanella sp. MR-4 chromosome I [462900-471299]	0	0	0	0
NC_008321:24|Shewanella sp. MR-4 chromosome I [2652600-2660049]	0	1	0	1
NC_008321:25|Shewanella sp. MR-4 chromosome I [2662500-2683199]	1	1	1	1
NC_008321:33|Shewanella sp. MR-4 chromosome I [3818200-3845149]	1	1	1	1

NC_008321:38|Shewanella sp. MR-4 chromosome I [4637250-4644449]	0	0	0	0
NC_008321:9|Shewanella sp. MR-4 chromosome I [1530100-1540849]	0	1	0	1
NC_008322:10|Shewanella sp. MR-7 chromosome I [1599100-1618399]	0	1	0	1
NC_008322:12|Shewanella sp. MR-7 chromosome I [1712000-1720199]	0	1	0	1
NC_008322:15|Shewanella sp. MR-7 chromosome I [2080050-2090549]	1	1	1	1
NC_008322:17|Shewanella sp. MR-7 chromosome I [2111200-2122799]	0	1	0	1
NC_008322:22|Shewanella sp. MR-7 chromosome I [2643800-2649199]	0	0	0	0
NC_008322:23|Shewanella sp. MR-7 chromosome I [2719950-2727749]	0	1	0	1
NC_008322:3|Shewanella sp. MR-7 chromosome I [645900-655049]	0	0	0	0
NC_008322:31|Shewanella sp. MR-7 chromosome I [4013600-4024449]	0	1	0	1
NC_008322:4|Shewanella sp. MR-7 chromosome I [726050-734299]	0	0	0	0
NC_008322:8|Shewanella sp. MR-7 chromosome I [1190750-1206249]	1	1	0	1
NC_008344:4|Nitrosomonas eutropha C91-C71 chromosome I [2097850-2103649]	0	0	1	1
NC_008344:6|Nitrosomonas eutropha C91-C71 chromosome I [2434100-2440699]	0	0	0	0
NC_008344:8|Nitrosomonas eutropha C91-C71 chromosome I [2470000-2480149]	0	0	1	1
NC_008344:9|Nitrosomonas eutropha C91-C71 chromosome I [2502850-2510349]	0	0	0	0
NC_008345:1|Shewanella frigidimarina NCIMB 400 chromosome I [1635950-1644199]	0	1	0	1
NC_008345:3|Shewanella frigidimarina NCIMB 400 chromosome I [2477150-2485449]	0	0	0	0
NC_008347:1|Maricaulis maris MCS10 chromosome I [942200-949349]	0	0	0	0
NC_008358:1|Hyphomonas neptunium ATCC 15444 chromosome I [1240700-1245449]	0	0	0	0
NC_008380:2|Rhizobium leguminosarum bv. viciae 3841 chromosome I [862200-876249]	0	1	0	1
NC_008380:3|Rhizobium leguminosarum bv. viciae 3841 chromosome I [882800-895999]	0	0	0	0
NC_008497:2|Lactobacillus brevis ATCC 367 chromosome I [346500-352949]	0	0	0	0
NC_008497:3|Lactobacillus brevis ATCC 367 chromosome I [742150-746849]	0	0	0	0
NC_008497:6|Lactobacillus brevis ATCC 367 chromosome I [1579500-1589499]	0	0	0	0
NC_008497:7|Lactobacillus brevis ATCC 367 chromosome I [1828550-1835699]	0	0	0	0
NC_008497:9|Lactobacillus brevis ATCC 367 chromosome I [2041150-2047149]	0	0	0	0
NC_008508:1|Leptospira borgpetersenii serovar Hardjo-bovis L550 chromosome I [785400-792899]	0	0	0	0
NC_008508:2|Leptospira borgpetersenii serovar Hardjo-bovis L550 chromosome I [1391450-1410299]	0	0	0	0
NC_008508:3|Leptospira borgpetersenii serovar Hardjo-bovis L550 chromosome I [1417450-1422099]	0	0	0	0
NC_008508:4|Leptospira borgpetersenii serovar Hardjo-bovis L550 chromosome I [2574500-2585349]	0	1	0	1
NC_008510:1|Leptospira borgpetersenii serovar Hardjo-bovis JB197 chromosome I [1347300-1367249]	0	0	0	0
NC_008510:2|Leptospira borgpetersenii serovar Hardjo-bovis JB197 chromosome I [1375300-1381299]	0	0	0	0
NC_008510:3|Leptospira borgpetersenii serovar Hardjo-bovis JB197 chromosome I [2607750-2618599]	0	0	0	0
NC_008510:4|Leptospira borgpetersenii serovar Hardjo-bovis JB197 chromosome I [2878250-2883399]	0	0	0	0
NC_008525:1|Pediococcus pentosaceus ATCC 25745 chromosome I [623350-636199]	0	0	0	0
NC_008525:2|Pediococcus pentosaceus ATCC 25745 chromosome I [976750-981899]	0	0	0	0
NC_008526:1|Lactobacillus casei ATCC 334 chromosome I [77650-84549]	0	1	0	1
NC_008526:2|Lactobacillus casei ATCC 334 chromosome I [95150-103949]	0	0	0	0
NC_008526:3|Lactobacillus casei ATCC 334 chromosome I [501000-506949]	0	1	0	1
NC_008526:4|Lactobacillus casei ATCC 334 chromosome I [570700-586099]	1	1	0	1
NC_008526:5|Lactobacillus casei ATCC 334 chromosome I [1942450-1948799]	0	0	0	0
NC_008526:6|Lactobacillus casei ATCC 334 chromosome I [1975850-1989549]	0	0	1	1
NC_008526:7|Lactobacillus casei ATCC 334 chromosome I [2380350-2389999]	0	0	1	1
NC_008528:1|Oenococcus oeni PSU-1 chromosome I [57200-62199]	0	0	0	0
NC_008528:2|Oenococcus oeni PSU-1 chromosome I [91250-95449]	0	0	0	0
NC_008528:3|Oenococcus oeni PSU-1 chromosome I [665900-672549]	0	0	0	0
NC_008528:4|Oenococcus oeni PSU-1 chromosome I [805750-808849]	0	0	0	0
NC_008528:5|Oenococcus oeni PSU-1 chromosome I [1410950-1417049]	0	0	0	0
NC_008529:1|Lactobacillus delbrueckii subsp. bulgaricus ATCC BAA-365 chromosome I [202750-221149]	0	1	0	1
NC_008529:10|Lactobacillus delbrueckii subsp. bulgaricus ATCC BAA-365 chromosome I [1459100-1464099]	0	0	0	0
NC_008529:11|Lactobacillus delbrueckii subsp. bulgaricus ATCC BAA-365 chromosome I [1517550-1534399]	0	1	1	1
NC_008529:12|Lactobacillus delbrueckii subsp. bulgaricus ATCC BAA-365 chromosome I [1638700-1648149]	0	1	0	1
NC_008529:13|Lactobacillus delbrueckii subsp. bulgaricus ATCC BAA-365 chromosome I [1667000-1686649]	0	1	1	1
NC_008529:14|Lactobacillus delbrueckii subsp. bulgaricus ATCC BAA-365 chromosome I [1769650-1781999]	0	1	0	1
NC_008529:15|Lactobacillus delbrueckii subsp. bulgaricus ATCC BAA-365 chromosome I [1842150-1846249]	0	0	0	0
NC_008529:2|Lactobacillus delbrueckii subsp. bulgaricus ATCC BAA-365 chromosome I [245200-252799]	0	0	0	0
NC_008529:4|Lactobacillus delbrueckii subsp. bulgaricus ATCC BAA-365 chromosome I [890600-903449]	0	1	0	1
NC_008529:5|Lactobacillus delbrueckii subsp. bulgaricus ATCC BAA-365 chromosome I [920850-934999]	0	1	0	1
NC_008529:8|Lactobacillus delbrueckii subsp. bulgaricus ATCC BAA-365 chromosome I [1045150-1067199]	0	1	0	1
NC_008529:9|Lactobacillus delbrueckii subsp. bulgaricus ATCC BAA-365 chromosome I [1139050-1149749]	0	0	0	0
NC_008531:1|Leuconostoc mesenteroides subsp. mesenteroides ATCC 8293 chromosome I [1419350-1428349]	0	0	0	0
NC_008536:1|Solibacter usitatus Ellin6076 chromosome I [3611600-3621449]	0	1	0	1
NC_008536:2|Solibacter usitatus Ellin6076 chromosome I [4926300-4933699]	0	0	0	0
NC_008555:1|Listeria welshimeri serovar 6b SLCC5334 chromosome I [1093100-1100449]	0	0	0	0
NC_008563:10|Escherichia coli APEC O1 chromosome I [1189900-1197149]	1	0	0	1
NC_008563:11|Escherichia coli APEC O1 chromosome I [1221000-1236449]	0	1	0	1
NC_008563:14|Escherichia coli APEC O1 chromosome I [1510150-1523449]	0	1	0	1
NC_008563:16|Escherichia coli APEC O1 chromosome I [1622700-1644949]	0	1	0	1
NC_008563:19|Escherichia coli APEC O1 chromosome I [2040100-2052549]	0	1	0	1
NC_008563:21|Escherichia coli APEC O1 chromosome I [2136450-2142749]	0	1	0	1
NC_008563:22|Escherichia coli APEC O1 chromosome I [2186150-2200449]	0	1	0	1
NC_008563:23|Escherichia coli APEC O1 chromosome I [2250700-2257449]	0	1	0	1
NC_008563:25|Escherichia coli APEC O1 chromosome I [2298500-2305749]	0	1	0	1
NC_008563:26|Escherichia coli APEC O1 chromosome I [2567700-2580549]	0	1	0	1
NC_008563:27|Escherichia coli APEC O1 chromosome I [2612800-2628349]	0	1	0	1
NC_008563:32|Escherichia coli APEC O1 chromosome I [3148800-3154049]	0	1	0	1
NC_008563:34|Escherichia coli APEC O1 chromosome I [3333900-3344349]	0	0	0	0
NC_008563:35|Escherichia coli APEC O1 chromosome I [3342950-3350799]	0	1	0	1
NC_008563:36|Escherichia coli APEC O1 chromosome I [3367550-3378649]	0	1	0	1
NC_008563:38|Escherichia coli APEC O1 chromosome I [3847850-3858549]	0	1	0	1
NC_008563:39|Escherichia coli APEC O1 chromosome I [3891000-3897699]	0	1	0	1
NC_008563:41|Escherichia coli APEC O1 chromosome I [4087450-4094499]	0	1	0	1
NC_008563:42|Escherichia coli APEC O1 chromosome I [4440950-4446599]	0	1	0	1
NC_008563:45|Escherichia coli APEC O1 chromosome I [4747000-4755149]	1	1	0	1
NC_008563:46|Escherichia coli APEC O1 chromosome I [4909600-4925449]	0	1	0	1
NC_008563:47|Escherichia coli APEC O1 chromosome I [4949450-4954599]	0	0	0	0
NC_008563:49|Escherichia coli APEC O1 chromosome I [5016800-5023449]	1	0	0	1
NC_008563:5|Escherichia coli APEC O1 chromosome I [321050-328249]	0	1	1	1

NC_008563:50|Escherichia coli APEC O1 chromosome I [5043600-5048749]	0	1	0	1
NC_008563:6|Escherichia coli APEC O1 chromosome I [347300-351249]	0	0	0	0
NC_008563:8|Escherichia coli APEC O1 chromosome I [1073200-1081249]	0	1	0	1
NC_008563:9|Escherichia coli APEC O1 chromosome I [1182500-1189549]	0	1	0	1
NC_008570:11|Aeromonas hydrophila subsp. hydrophila ATCC 7966 chromosome I [3225100-3265749]	0	0	1	1
NC_008577:11|Shewanella sp. ANA-3 chromosome I [1890550-1905799]	0	0	0	0
NC_008577:13|Shewanella sp. ANA-3 chromosome I [2117650-2124349]	0	1	0	1
NC_008577:16|Shewanella sp. ANA-3 chromosome I [2258100-2262499]	0	0	0	0
NC_008577:19|Shewanella sp. ANA-3 chromosome I [2429100-2445299]	1	1	0	1
NC_008577:20|Shewanella sp. ANA-3 chromosome I [2520600-2527149]	0	1	0	1
NC_008577:23|Shewanella sp. ANA-3 chromosome I [2913150-2920849]	0	0	0	0
NC_008577:27|Shewanella sp. ANA-3 chromosome I [3252750-3259499]	0	0	0	0
NC_008577:33|Shewanella sp. ANA-3 chromosome I [4613800-4628849]	0	1	1	1
NC_008577:34|Shewanella sp. ANA-3 chromosome I [4629350-4639999]	0	1	0	1
NC_008577:36|Shewanella sp. ANA-3 chromosome I [4913700-4920149]	0	0	0	0
NC_008577:7|Shewanella sp. ANA-3 chromosome I [1589000-1604599]	0	1	0	1
NC_008577:9|Shewanella sp. ANA-3 chromosome I [1696200-1704449]	0	1	0	1
NC_008578:1|Acidothermus cellulolyticus 11B chromosome I [888650-895049]	0	0	0	0
NC_008609:12|Pelobacter propionicus DSM2379 chromosome I [2000200-2059849]	0	0	1	1
NC_008609:16|Pelobacter propionicus DSM2379 chromosome I [2472550-2483549]	0	0	1	1
NC_008609:18|Pelobacter propionicus DSM2379 chromosome I [2645750-2663299]	0	0	0	0
NC_008609:28|Pelobacter propionicus DSM2379 chromosome I [3737150-3753999]	0	0	0	0
NC_008609:4|Pelobacter propionicus DSM2379 chromosome I [265700-276349]	0	0	1	1
NC_008611:1|Mycobacterium ulcerans Agy99 [2630750-2634199]	0	0	0	0
NC_008618:5|Bifidobacterium adolescentis ATCC15703 chromosome I [1210900-1218199]	0	1	0	1
NC_008618:8|Bifidobacterium adolescentis ATCC15703 chromosome I [1531000-1543149]	0	1	0	1
NC_008639:1|Chlorobium phaeobacteroides DSM266 chromosome I [87600-93399]	0	0	0	0
NC_008639:10|Chlorobium phaeobacteroides DSM266 chromosome I [2566350-2583399]	0	0	0	0
NC_008639:11|Chlorobium phaeobacteroides DSM266 chromosome I [2708000-2717349]	0	0	0	0
NC_008639:13|Chlorobium phaeobacteroides DSM266 chromosome I [2998350-3012449]	0	0	1	1
NC_008639:4|Chlorobium phaeobacteroides DSM266 chromosome I [1469250-1478849]	0	0	0	0
NC_008639:5|Chlorobium phaeobacteroides DSM266 chromosome I [1577850-1586249]	0	0	0	0
NC_008639:6|Chlorobium phaeobacteroides DSM266 chromosome I [2362600-2373699]	0	0	0	0
NC_008639:8|Chlorobium phaeobacteroides DSM266 chromosome I [2467050-2473849]	0	0	0	0
NC_008639:9|Chlorobium phaeobacteroides DSM266 chromosome I [2548600-2558049]	0	0	0	0
NC_008700:18|Shewanella amazonensis SB2B chromosome I [2706350-2729099]	0	1	0	1
NC_008709:2|Psychromonas ingrahamii 37 chromosome I [1988500-1996099]	0	1	0	1
NC_008709:3|Psychromonas ingrahamii 37 chromosome I [2330950-2337199]	0	0	0	0
NC_008709:4|Psychromonas ingrahamii 37 chromosome I [3291650-3296599]	0	0	0	0
NC_008740:21|Marinobacter aquaeolei VT8 chromosome I [1830350-1856099]	0	1	0	1
NC_008740:27|Marinobacter aquaeolei VT8 chromosome I [2929500-2949149]	0	1	0	1
NC_008750:3|Shewanella sp. W3-18-1 chromosome I [386750-390899]	0	1	0	1
NC_008750:6|Shewanella sp. W3-18-1 chromosome I [4569550-4576099]	0	1	1	1
NC_008767:1|Neisseria meningitidis FAM18 chromosome I [24350-33849]	0	0	0	0
NC_008767:10|Neisseria meningitidis FAM18 chromosome I [616500-631849]	0	0	0	0
NC_008767:11|Neisseria meningitidis FAM18 chromosome I [809250-815949]	0	0	0	0
NC_008767:12|Neisseria meningitidis FAM18 chromosome I [881500-892499]	0	0	1	1
NC_008767:14|Neisseria meningitidis FAM18 chromosome I [1326000-1336099]	0	1	1	1
NC_008767:15|Neisseria meningitidis FAM18 chromosome I [1741200-1750499]	0	0	0	0
NC_008767:16|Neisseria meningitidis FAM18 chromosome I [1833350-1844199]	0	0	0	0
NC_008767:17|Neisseria meningitidis FAM18 chromosome I [1890600-1900149]	0	0	0	0
NC_008767:2|Neisseria meningitidis FAM18 chromosome I [56900-68249]	0	0	0	0
NC_008767:20|Neisseria meningitidis FAM18 chromosome I [2131700-2143099]	0	0	0	0
NC_008767:5|Neisseria meningitidis FAM18 chromosome I [277750-285349]	0	0	1	1
NC_008767:7|Neisseria meningitidis FAM18 chromosome I [456800-464099]	0	0	0	0
NC_008767:8|Neisseria meningitidis FAM18 chromosome I [463600-472049]	0	0	0	0
NC_008767:9|Neisseria meningitidis FAM18 chromosome I [550300-557299]	0	0	1	1
NC_008781:6|Polaromonas naphthalenivorans CJ2 chromosome I [4183300-4213449]	0	0	1	1
NC_008817:1|Prochlorococcus marinus MIT 9515 chromosome I [1229400-1234599]	0	0	0	0
NC_008819:1|Prochlorococcus marinus NATL1A chromosome I [795950-808449]	0	1	0	1
NC_008820:1|Prochlorococcus marinus MIT 9303 chromosome I [95500-145549]	0	1	0	1
NC_008820:10|Prochlorococcus marinus MIT 9303 chromosome I [1563000-1566449]	0	0	0	0
NC_008820:11|Prochlorococcus marinus MIT 9303 chromosome I [1664200-1676499]	0	1	0	1
NC_008820:12|Prochlorococcus marinus MIT 9303 chromosome I [1771500-1795699]	0	1	0	1
NC_008820:13|Prochlorococcus marinus MIT 9303 chromosome I [1823600-1836849]	0	1	0	1
NC_008820:16|Prochlorococcus marinus MIT 9303 chromosome I [2600150-2605899]	0	0	0	0
NC_008820:2|Prochlorococcus marinus MIT 9303 chromosome I [214150-223699]	0	0	0	0
NC_008820:5|Prochlorococcus marinus MIT 9303 chromosome I [1146100-1152949]	0	0	0	0
NC_008820:6|Prochlorococcus marinus MIT 9303 chromosome I [1157750-1165699]	0	0	0	0
NC_008820:7|Prochlorococcus marinus MIT 9303 chromosome I [1362500-1376249]	0	1	0	1
NC_009012:1|Clostridium thermocellum ATCC 27405 chromosome I [1321600-1327999]	0	1	0	1
NC_009012:2|Clostridium thermocellum ATCC 27405 chromosome I [1342850-1350499]	0	0	0	0
NC_009012:3|Clostridium thermocellum ATCC 27405 chromosome I [1374200-1381999]	0	0	1	1
NC_009012:4|Clostridium thermocellum ATCC 27405 chromosome I [1644550-1650549]	0	0	0	0
NC_009012:5|Clostridium thermocellum ATCC 27405 chromosome I [2016050-2039449]	0	0	0	0
NC_009012:6|Clostridium thermocellum ATCC 27405 chromosome I [2051400-2067849]	0	0	0	0
NC_009052:1|Shewanella baltica OS155 chromosome I [73300-79499]	0	1	0	1
NC_009052:11|Shewanella baltica OS155 chromosome I [2518150-2525549]	1	1	0	1
NC_009052:12|Shewanella baltica OS155 chromosome I [2702300-2705099]	0	0	1	1
NC_009052:19|Shewanella baltica OS155 chromosome I [3668250-3676599]	0	1	1	1
NC_009052:21|Shewanella baltica OS155 chromosome I [4174400-4182449]	1	0	0	1
NC_009052:22|Shewanella baltica OS155 chromosome I [4246000-4253399]	0	0	0	0
NC_009052:25|Shewanella baltica OS155 chromosome I [5092550-5101399]	0	1	1	1
NC_009052:6|Shewanella baltica OS155 chromosome I [1837150-1843549]	0	1	0	1
NC_009053:1|Actinobacillus pleuropneumoniae L20 chromosome I [900600-908899]	0	1	0	1
NC_009053:2|Actinobacillus pleuropneumoniae L20 chromosome I [1089900-1102999]	0	1	1	1
NC_009053:3|Actinobacillus pleuropneumoniae L20 chromosome I [1673150-1683299]	0	0	0	0
NC_009053:4|Actinobacillus pleuropneumoniae L20 chromosome I [1801450-1810699]	0	0	0	0
NC_009092:44|Shewanella loihica PV-4 chromosome I [4546350-4568949]	0	0	1	1

NC_009138:13|Herminiimonas arsenicoxydans chromosome I [1521450-1533199]	0	1	1	1
NC_009138:15|Herminiimonas arsenicoxydans chromosome I [1549600-1566099]	0	1	1	1
NC_009138:16|Herminiimonas arsenicoxydans chromosome I [1579550-1599399]	0	1	0	1
NC_009138:17|Herminiimonas arsenicoxydans chromosome I [1844600-1865349]	0	1	1	1
NC_009138:18|Herminiimonas arsenicoxydans chromosome I [1872550-1882749]	0	1	1	1
NC_009138:23|Herminiimonas arsenicoxydans chromosome I [3158400-3187849]	0	1	1	1
NC_009138:4|Herminiimonas arsenicoxydans chromosome I [468700-489399]	0	1	0	1
NC_009138:5|Herminiimonas arsenicoxydans chromosome I [494800-513899]	0	1	1	1
NC_009142:1|Saccharopolyspora erythraea NRRL 2338 [2821750-2824099]	0	1	0	1
NC_009142:2|Saccharopolyspora erythraea NRRL 2338 [6216700-6222149]	0	0	0	0
**Percentage of confirmed predictions**	**3.83%**	**48.72%**	**16.45%**	**55.35%**
